# Severe and frequent extreme weather events undermine economic adaptation gains of tree-species diversification

**DOI:** 10.1038/s41598-024-52290-2

**Published:** 2024-01-25

**Authors:** Jasper M. Fuchs, Kai Husmann, Jan Schick, Matthias Albert, Jussi Lintunen, Carola Paul

**Affiliations:** 1 Department of Forest Economics and Sustainable Land-Use Planning, University of Göttingen, Büsgenweg 1, 37077 Göttingen, Germany; 2 Department of Forest Growth, Northwest German Forest Research Institute, Grätzelstraße 2, 37079 Göttingen, Germany; 3 Faculty of Forest Sciences and Forest Ecology, University of Göttingen, Büsgenweg 5, 37077 Göttingen, Germany; 4 Natural Resources Institute Finland (Luke), Latokartanonkaari 9, 00790 Helsinki, Finland; 5 Centre of Biodiversity and Sustainable Land Use, University of Göttingen, Büsgenweg 1, 37077 Göttingen, Germany

**Keywords:** Climate-change adaptation, Environmental economics, Climate-change impacts, Natural hazards

## Abstract

Forests and their provision of ecosystem services are endangered by climate change. Tree-species diversification has been identified as a key adaptation strategy to balance economic risks and returns in forest stands. Yet, whether this synergy between ecology and economics persists under large-scale extreme weather events remains unanswered. Our model accounts for both, small-scale disturbances in individual stands and extreme weather events that cause spatio-temporally correlated disturbances in a large number of neighboring stands. It economically optimizes stand-type allocations in a large forest enterprise with multiple planning units. Novel components are: spatially explicit site heterogeneity and a comparison of economic diversification strategies under local and regionally coordinated planning by simplified measures for $\alpha$, $\beta$, and $\gamma$-diversity of stand types. $\alpha$-diversity refers to the number and evenness of stand types in local planning units, $\beta$-diversity to the dissimilarity of the species composition across planning units, and $\gamma$-diversity to the number and evenness of stand types in the entire enterprise. Local planning led to stand-type diversification within planning units ($\alpha$-diversity), while regionally coordinated planning led to diversification across planning units ($\beta$-diversity). We observed a trend towards homogenization of stand-type composition likely selected under economic objectives with increasing extreme weather events. No diversification strategy fully buffered the adverse economic consequences. This led to fatalistic decisions, i.e., selecting stand types with low investment risks but also low resistance to disturbances. The resulting forest structures indicate potential adverse consequences for other ecosystem services. We conclude that high tree-species diversity may not necessarily buffer economic consequences of extreme weather events. Forest policies reducing forest owners’ investment risks are needed to establish stable forests that provide multiple ecosystem services.

## Introduction

Adapting forests to climate change and increasing disturbances^1^ is a pressing challenge for forest management. Suitable adaptation strategies to maintain ecosystem functioning and the provision of ecosystem services are urgently needed.

Spatial diversification of management regimes is discussed as a promising concept for adapting future forests to climate change and establishing more resilient forest ecosystems^2,3^. Tree-species diversification is a key adaptation strategy expected to buffer the negative impacts of climate change on ecosystem functioning and ecosystem service provision^2,4,5^. Mixing species with different traits and responses to climate change and disturbances^6,7^ promises higher resilience^8^ with lower variability in ecosystem service provision^9,10^. To what extent such diversification strategies are also economically efficient is under debate^5,11^.

Decisions of forest owners who follow economic objectives are highly relevant for future forest trajectories as 85 % of European forests are managed and about 50 % are privately owned^12^. Wood production, income generation, and forest stocks as an asset are relevant aspects of forest management decisions in Europe^13–15^. Selling wood is the key source to financing forest management^13^. However, the economic potential of wood production is negatively affected by climate-driven disturbances. For instance, the drought years 2018-2020 caused losses of about 12.7 billion Euros in Germany^16^. Decisions of forest owners facing such economic risks have consequences for future ecosystem functioning and provisioning of ecosystem services. Understanding mechanisms behind likely decisions of forest owners thus fundamentally contributes to scientific scenario development and policy design.

Modern Portfolio Theory^17^ has been proposed to study diversification strategies from an economic perspective by interpreting tree species as assets in a portfolio^18,19^. Combinations of productive high-risk species with more stable low-risk species promise to balance economic risks and returns^20,21^. The ecological advantages of diversification, e.g., stabilization towards disturbances^22,23^, can translate into economic advantages, in particular for risk-averse decision-makers. Hence, tree-level mixtures provide additional positive economic effects beyond product diversification. Simulations suggest that establishing mixed stands with adapted species compositions may mitigate adverse economic consequences of climate change^24^.

Most of the studies cited above have focused on the consequences of long-term changes in average climate variables on tree-species selection. An important, but so far understudied, aspect of climate change is increasing extreme weather events, i.e., sudden, severe weather conditions such as storms that can cause drastic disturbances in forests. Forzieri *et al.*^25^ found climate change to increase the vulnerability of forests to such sudden events. Empirical disturbance maps^26^ illustrate changing disturbance patterns. Severe compound extremes in 2018-2020 in Central Europe^27,28^ evoked the discussion that forest management has reached a tipping point, requiring not only incremental adaptations in management practices to climate change, but also a fundamental transition towards new forest management systems^28^.

Economic studies on extreme weather events mostly focus on short-term consequences of extreme weather events on wood markets^29–31^. Möhring *et al.*^16^ discussed long-term consequences of reduced yield potentials and altered age-class distributions. Incorporating longer-term consequences in a stand-level simulation model, Knoke *et al.*^32^ illustrated the relevance of extremes for managing spruce stands. However, larger spatial scales might be relevant as decision-makers with small and spatially clustered properties face a high probability that large parts of the enterprise are simultaneously affected. In contrast, decision-makers at the regional level can diversify the species allocation across large spatial scales rather than within a small number of stands^33,34^. The degree of stand-to-regional tree-species diversification best suited to economically buffer extreme weather events remains unclear.

In ecology, diversity at different spatial scales can be described by $\alpha$, $\beta$, and $\gamma$-diversity. Regarding tree-species diversification, Sebald *et al.*^35^ related stand-level species composition to $\alpha$-diversity, species diversification across stands to $\beta$-diversity, and the overall species composition to $\gamma$-diversity. They found high $\beta$-diversity to be at least as promising as high $\alpha$-diversity in adapting forests to amplifying disturbance events with regard to biomass stocks. Transferring this concept to economic tree-species allocation at a regional scale could help to identify promising adaptation strategies against increasing extreme weather events. As an analogous economic effect, one may expect that tree-species diversification across a larger area with heterogeneous site conditions ($\beta$-diversity) would provide more flexibility than diversification strategies aiming for a high local $\alpha$-diversity. This spatial flexibility could avoid economic losses caused by allocating low-productive species to highly productive sites when increasing species diversity to reduce economic risks. A higher economic performance of adapting not only local tree-species composition but also species allocation across a region would demonstrate an additional economic buffering capacity of large-scale diversification strategies. To the best of our knowledge, neither the economic buffering capacity of tree-species allocation at regional scales nor its specific relevance for buffering the economic consequences of enhanced extreme-event patterns have yet been assessed. Taking this regional perspective is highly relevant for both public forest management planning and understanding likely behavior of large private forest enterprises. For example, 29 % of German forests are owned by the federal states and managed by public forest enterprises responsible for about 240,000 ha of forest land on average^36^. Regional analyses may also allow for testing the efficiency and effectiveness of regionally coordinated management, e.g., spatial targeting of land-use policy, compared to independent local decision-making. For instance, Huber *et al.*^37^ showed for grasslands that regionally coordinated land-use decisions increased ecosystem service provision as compared to decision-making at the individual farm level. Such approaches are so far missing in forestry.

**Figure 1:**
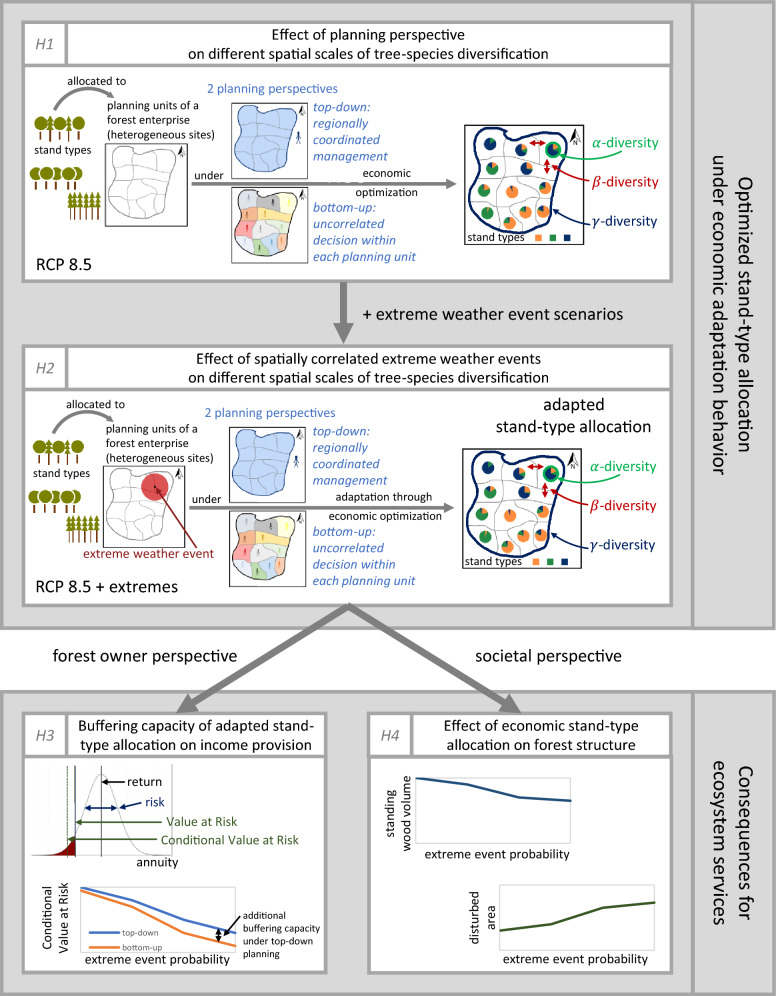
Study design and assignment of model outputs to hypotheses *H1*–*H4*. RCP 8.5 refers to the climate change scenario (see Sect. *Model and data*).

*The objective of our study is to better understand the opportunities and limitations of spatial diversification strategies in order to buffer economic consequences of extreme weather events.* We develop a new simulation-optimization approach, which extends earlier portfolio approaches by spatially explicit allocations of tree species to local planning units of a large forest enterprise (Fig. 1, *H1*). We introduce spatially explicit components to account for multiple planning units, heterogeneous site conditions, and spatially correlated extreme weather events (Fig. 1, *H2*). This allows us to test at a regional scale whether the synergies of increasing tree-species diversity regarding ecological and economic objectives are stable across scales and under increasing extreme weather events. We apply simplified indicators for different diversification strategies analogous to $\alpha$, $\beta$, and $\gamma$-diversity of dominant tree species at a regional scale. We compare bottom-up diversification of stand types by individual decision-makers in the planning units ($\alpha$-diversity) with spatially coordinated top-down planning in the forest enterprise, assuming one decision-maker who accounts for correlations across planning units ($\alpha$, $\beta$, and $\gamma$-diversity) to expand the understanding of how (1) these planning perspectives and (2) spatial patterns of extreme weather events likely affect future forests when decision-makers pursue economic objectives.

We apply this approach to a stylized forest enterprise, representing a large forest decision unit with multiple planning units. Realistic levels of spatial heterogeneity and spatial clustering of forest land are derived from a public forest enterprise in Central Germany (about 320,000 ha). This perspective illustrates the consequences of decision-making in typical public forest agencies in Germany or large private forest enterprises with forest properties scattered across a region, and represents spatial scales relevant for regional policy-makers. We optimize the allocation of area shares to 8 different stand types, monocultures or mixed stands of common tree species in the study region, in each planning unit of the forest enterprise, i.e., we optimize the planning units’ stand-type composition or portfolio. We compare the allocation of stand types to planning units under bottom-up and top-down planning. All decision-makers seek to balance economic risks and returns, expressed in a risk-averse objective function (Conditional Value at Risk, see Sect. *Model and data*). We assume that they aim to adapt the stand-type allocation in order to maintain the economic objective function under increased extreme weather events. In contrast to trend-adaptive or proactive concepts described in Yousefpour *et al.*^38^, we assume that decision-makers have perfect knowledge of future disturbance probabilities. Their expectations on future risks and returns of the stand types differ only due to differences in site conditions between planning units. Return distributions are derived from a stochastic Monte-Carlo simulation with independent, climate-driven stand-level disturbances, i.e., stand-replacing events affecting only one stand, and additional, spatially correlated extreme weather events, which likely affect neighboring stands simultaneously. Input information on stand growth and management^39,40^, planting costs^41^, wood revenues and harvest costs^42^, and stand-level disturbance probabilities^22^ are representative for the region. It is important to note, however, that the results are sensitive to these input variables and assumptions, such as planting costs or the stabilizing effect of species mixture, which we account for by applying extensive sensitivity analyses (see *Sect. Model and data*). Our baseline stand-level disturbance probabilities refer to the projected climate in the planning units under the RCP 8.5 scenario (2061-2080) (see *Sect. Model and data*). Our study focuses on different scenarios of additional, spatially correlated extreme weather events. We assume this fixed baseline climate-change scenario, i.e., we do not account for deep climate uncertainty^43^. We performed *ceteris-paribus* analyses on the model’s selection of diversification strategies under different extreme-event scenarios. These extreme-event scenarios are defined by disturbance intensities, i.e., probabilities that a stand is affected by an event, the expected number of events, and their size. We calculate 7 indicators to describe potential diversification strategies chosen by the model. At the scale of the planning units, we interpret the number and evenness of stand types as measures for the local compositional diversity, reflecting $\alpha$-diversity. Dissimilarity metrics between the stand-type composition of the planning units indicate the spatial heterogeneity of the stand-type allocation in the enterprise, reflecting $\beta$-diversity. Number of stand types and evenness of the stand-type composition at the enterprise scale reflect $\gamma$-diversity. The share of mixed stands and deciduous species are simplified proxies for structural and functional diversity. While our study focuses on the provisioning service income from wood production (Fig. 1, *H3*), we additionally discuss potential consequences for forest attributes under economic decision-making. These forest attributes, such as average standing wood volume, mean diameter of trees, species composition, or annually disturbed area, provide a qualitative indication of likely consequences for the provision of other ecosystem services^44^, such as carbon sequestration and habitat provision (Fig. 1, *H4*).

Within this framework, we test the following hypotheses on economically optimal adaptation behavior in terms of adapting the stand-type allocation to buffer adverse consequences of additional extreme weather events on the risk-averse economic objective function (Fig. 1):

1. *Economically optimal stand-type diversification is higher under bottom-up planning than under top-down planning when excluding extreme weather events.*
2. *With an increasing intensity of extreme weather events, the economically optimal degree of stand-type diversification increases under both planning perspectives.*
3. *Economically optimal stand-type allocation across planning units buffers the adverse economic consequences of extreme weather events.*
4. *Economically optimal stand-type allocation under additional extreme weather events accepts an increase in the annually disturbed area compared to optimal stand-type allocations under stand-level disturbances only.*

Our approach is not designed to give spatially explicit management recommendations. We derived the optimal composition of stand types for bare land, i.e., not taking the current management into account, and chose a coarse resolution for spatial heterogeneity. Consequently, we interpret the simulated forests in this region as a large model enterprise and seek to explore general diversification patterns of purely economic decision-making under different scenarios. These results contribute to the understanding of likely decisions of forest owners who follow economic objectives dependent on both size of forest property and future extreme-event patterns.

## Model and data

We developed a simulation-optimization model for a large model forest enterprise. The enterprise consists of 24 internally homogeneous *planning units* (forest districts with 5,500 to 19,000 

ha), which differ in their site productivity and climate conditions. The model solves the economic planning problem of allocating different *stand types* defined by species compositions, either monocultures of different species or mixed stands of two species, to these planning units. The allocation is optimized for a risk-averse decision-maker, whose objective is to generate income from wood production but who also accounts for economic risks. We distinguished between the two planning perspectives (Fig. 1), *bottom-up planning* (multiple local planners with the same objective function) and *top-down planning* (one regional planner). For the bottom-up perspective, we optimized the area shares of the stand types (stand-type portfolio) for each planning unit individually. In contrast, the top-down perspective assumed regionally coordinated decision-making at the enterprise level, optimizing the area shares of the stand types for all planning units simultaneously in one objective function. The top-down decision-maker can benefit from site heterogeneity and consider risk correlations across planning units, thus suggesting different management under the two planning perspectives.

We built on earlier simulation-optimization models for economic tree-species selection^18,24,45^ based on Modern Portfolio Theory^17^. Markowitz’s portfolio framework is based on fluctuating returns of assets. In this context, we interpret a stand type’s expected annual net returns as economic return and its standard deviation as economic risk. Combining stands whose returns are not perfectly correlated in a portfolio reduces the forest owner’s economic risk. Portfolio optimization models identify portfolio compositions that balance the economic risks and returns. We extended these existing stand-level models with novel, spatially explicit components, allowing for the simulation of forest enterprises with multiple planning units, heterogeneous site conditions, and a spatial representation of extreme weather events. The extended model accounts for three reasons for tree mortality: regular harvests, stand-level disturbances, and extreme weather events. Regular harvest operations, i.e., thinning and final harvests at the end of the rotation, are covered by regional yield tables. Stand-level disturbances and extreme weather events are stochastic model components that refer to different spatial patterns of stand-replacing disturbances with subsequent salvage harvests (flow chart in Supplementary Fig. A3).

**Figure 2:**
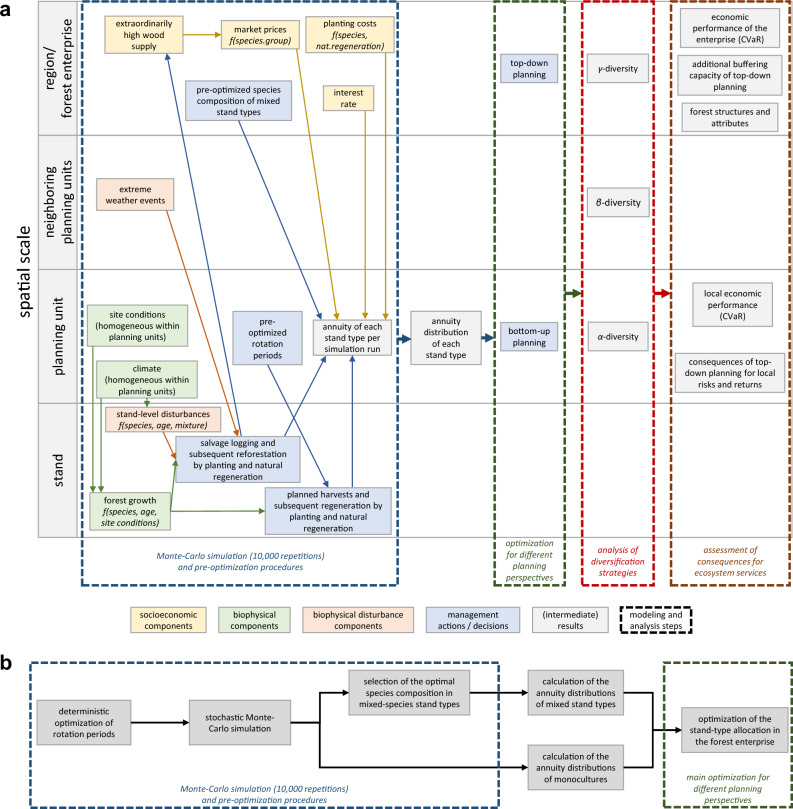
Overview of the methodological approach. Simplified overview of model components, their spatial scales, and the different steps of the simulation and analysis (**a**) and flow chart of the simulation and optimization steps in the model (**b**). The input variables for the pre-optimizations are not shown, but are explained in the text.

The model consists of several simulation and (pre-)optimization procedures. The pre-optimization of the tree species’ rotation periods and the species compositions in mixed stand types ensured that we compare only economically optimal stand management. In the main optimization, these pre-optimized stand-types were then offered to the spatial allocation model, which optimizes stand-type compositions of each planning unit of the forest enterprise. This multi-stage approach (described below) strongly reduced the computational burden. This enabled us to account for the relevant interdependencies between market risks, biophysical risks, and extreme weather events at different spatial scales of the non-linear model. A simultaneous and endogenous optimization of stand management within the main optimization would have impeded the consideration of these complex interdependencies. We carefully discuss potential implications of our approach in the Discussion section (section on *H3*). It is also important to note that the stand types, selected according to this optimization procedure, do not necessarily reflect the most common silvicultural management in the study region, but do reflect possible management options. We perform the following optimizations (Fig. 2b):

**1. Pre-optimization of rotation periods:** The model first determines the rotation periods in 10-year age steps maximizing the expected annuity for the simulated tree species. This optimization accounts for stand-level disturbance risks by applying a deterministic approach that weights the economic return of the different possible salvage-harvest ages and a planned final harvest with their occurrence probabilities, following the approaches by Möllmann and Möhring^46^ and Staupendahl and Möhring^47^. Rotation periods are optimized separately for monocultures and for all possible species mixtures, i.e., accounting for potential changes in optimal rotation periods if species mixtures reduce the disturbance risk^22^ (for details see Supplementary Methods A1.1.1).

**2. Pre-optimization of species shares in mixed stands:** The second step is based on a Monte-Carlo simulation. The model simulates for each planning unit the forest growth and economic performance of the monocultures of all 5 species, as well as all possible mixed stands (2 species, 10%-steps of area shares), and applies the optimized rotation periods. The simulation accounts for stochastic stand-level disturbances, extreme weather events, and fluctuations of prices on the wood markets (for an overview of the model components and spatial scales, see Fig. 2a). For the mixed-species stand types, the model selects the species composition that maximizes the stand-level Conditional Value at Risk based on the Monte-Carlo simulation (for details see Supplementary Methods A1.1.1).

**3. Main optimization of stand-type compositions in the planning units:** The following main optimization allocates area shares of the pre-optimized stand types (defined by species composition) to the planning units of the forest enterprise aiming to maximize the Conditional Value at Risk based on the Monte-Carlo simulation (for details see below).

### Optimization of the stand-type composition in the planning units

The optimization allocates continuous shares of stand types to each planning unit in order to maximize the economic objective function Conditional Value at Risk ($CV\!aR$). $CV\!aR$ is based on the annuity distribution (land rent) derived by 10,000 Monte-Carlo repetitions of the growth and management simulation with stochastic disturbances and market fluctuations. For bottom-up planning, we evaluated the objective function separately for each planning unit *p*. For top-down planning, we evaluated the objective function for all $P = 24$ planning units in one optimization.

#### Objective function

We modeled risk-averse decision-making aiming at wood-production income by maximizing the $CV\!aR$ of the annual economic return or annuity, *R*. Value at Risk ($V\!aR$) and $CV\!aR$ are measures for downside risks and are often applied for forest economic planning problems to mimic risk-averse decision-making^45,48^. We chose $CV\!aR$ because it better accounts for potential changes in the shape of the annuity distribution, $F_R$, such as an increasing left skew due to seldom very poor outcomes under additional extreme weather events. $CV\!aR$ is defined as the expected value of all realizations of the annuity lower than a given quantile of the distribution of the joint annuity of all planning units in the forest enterprise,

1 $$\begin{aligned} \begin{aligned} CV\!aR_{\alpha }&= {\mathbb {E}}\left[ R \, \bigg | R< F_{R}^{-1}\left( \alpha \right) \right] \\&\quad = {\mathbb {E}}\left[ R \, \bigg | R < V\!aR_{\alpha }\right] . \end{aligned} \end{aligned}$$

The quantile, $F_{R}^{-1}\left( \alpha \right)$, which equals the $V\!aR_{\alpha }$, is defined by the probability $\alpha$, which expresses the decision-maker’s degree of risk aversion. We thus consider a decision-maker who seeks to maximize the expected economic outcome in the worst cases defined by the $\alpha$-quantile of the joint annuity distribution.

The optimization searches for the optimal allocation of area shares, $w_{p,s}$, of the *S* stand types $s = 1, 2, \dots , S$ to the *P* planning units $p = 1, 2, \dots , P$ by maximizing the simulated $CV\!aR$ dependent on the allocation array, *w*, which indicates the stand-type composition in each planning unit,

2 $$\begin{aligned} \begin{aligned} \max _{w \in \left[ 0, 1\right] ^{P \times S}} CV\!aR_{\alpha }\left( w\right) . \end{aligned} \end{aligned}$$

The simulated $CV\!aR(w)$ is based on the empirical joint annuity distribution, $F_{R\left( w\right) }$, consisting of $n=$10,000 realizations calculated based on the Monte-Carlo simulation of the different stand types in the planning units with $i = 1, 2, \dots , n$ repetitions. The joint annuity distribution accounts for the consequences of spatiotemporal correlations in the disturbance and market simulation on the realizations of the enterprise’s annuity. A realization *i* of this annuity [€$\,{{\text{ha}}^{-1}\,{\text{a}}^{-1}}$] is

3 $$\begin{aligned} R_i\left( w\right) = \sum _{p = 1}^P \frac{A_p}{A} \sum _{s = 1}^S w_{p,s} R_{p,s, i}, \end{aligned}$$

with the area of planning unit *p*, $A_p$[ha], and the area of the enterprise, $A=\sum _p A_p$ [ha].

The simulated $CV\!aR(w)$ can now be defined as

4 $$\begin{aligned} CV\!aR_{\alpha }\left( w\right) := \frac{1}{\alpha \, n} \sum _{i\in I_{\alpha }(w)} R_i(w), \end{aligned}$$

where the set of realizations included into $CV\!aR(w)$ is

5 $$\begin{aligned} I_{\alpha }(w):=\{ i | R_i(w) \le {V\!aR}_{\alpha }(w)\}. \end{aligned}$$

Again, the $V\!aR$ is equal to the empirical quantile $F_{R(w)}^{-1}\left( \alpha \right)$. Since all the land in a planning unit *p* needs to be allocated to some stand type, the optimization problem is constrained by

6 $$\begin{aligned} \sum _{s = 1}^S w_{p,s} = 1, \forall p. \end{aligned}$$

The annuity is the land rent calculated from the Land Expectation Value *LEV *[€ ${{\text{ha}}^{-1}}]$^49^ as

7 $$\begin{aligned} R_{p,s,i} = r \, LEV_{p,s,i}, \end{aligned}$$

with the interest rate *r*. The *LEV* is the net present value of the bare land that can be obtained by perpetual forest management. In contrast to the original formulation according to Faustmann^49^ as the net present value of an eternal periodical rent, we approximated it by the net present value of the cash flows of the simulated 500 years. We assumed a positive time preference quantified by an interest rate of $r = {1.5}\,\%$^50^; net returns in the future thus have a lower contribution to the decision-maker’s objective function than today’s net returns. In the Monte-Carlo simulation, we calculated the *LEV* of stand type *s* in planning unit *p* and Monte-Carlo run *i* as

8 $$\begin{aligned} LEV_{p,s,i} = \sum _{t = 0}^{500} \left( -c_{pl,p,s,i,t} + v_{th,p,s,i,t} - c_{th,s,i,t} + v_{ha,p,s,i,t} - c_{ha,p,s,i,t}\right) \frac{1}{\left( 1 + r\right) ^t}, \end{aligned}$$

with the simulation time $t = 0, 10, 20, \dots , 500$, the planting costs $c_{pl} [$€ ${{\text{ha}}^{-1}}]$, the revenues and costs of thinnings $v_{th} [$€ ${{\text{ha}}^{-1}}]$ and $c_{th} [$€ ${{\text{ha}}^{-1}}]$, respectively, and the revenues and costs of final harvests $v_{ha} [$€ ${{\text{ha}}^{-1}}]$ and $c_{ha} [$€ ${{\text{ha}}^{-1}}]$, respectively. The costs and revenues get value zero if there are no related activities in the forest stand at period *t*.

A moderate risk aversion of a decision-maker is usually represented by $\alpha = 5\,\%$ in studies applying the $V\!aR$^45^. We chose $\alpha = {10}\,\%$ for our model, as the $CV\!aR$ is by construction more conservative than the $V\!aR$.

#### Optimization algorithm

With 192 decision variables, box constraints, 24 equality constraints, and an empirical distribution of unknown properties, the optimization problem was highly demanding. The difficulties arise from the fact that the decision variable, *w*, affects the set of realizations $I_{\alpha }(w)$, defined in Equation (5), that are included in the calculation of the objective function (Eq. (4)). In a risk-neutral case ($\alpha = 1$), all realizations would be included in the expected value calculation and the problem would be a large linear program. Additionally, in the risk-averse case, the determination of the set, $I_{\alpha }(w)$, increases computational burden as it requires finding a specific quantile, ${V\!aR}_{\alpha }(w)$, from the empirical distribution of 10,000 elements.

We tested a set of simulated annealing and evolutionary methods and chose the Global Differential Evolution approach, which has often been applied to portfolio problems^51,52^ and is implemented in the R-package DEoptim^53^. It best reproduced solutions for simpler examples solved by enumerations and provided plausible and reproducible results for our large-scale optimizations.

### Stand types, spatial heterogeneity and forest growth

We applied the model to search for compositions of 8 stand types in the planning units. The planning units differ in their site conditions (climate, soil), which implement site heterogeneity in our model enterprise. These site conditions are relevant for forest growth and disturbance probabilities. We built the simulated model enterprise based on public forest service data of Lower Saxony, Germany (about 320,000 ha forests). This allowed us to consider realistic levels of spatial heterogeneity, but also the spatial arrangements and sizes of planning units (5500 to 19,000 ha).

#### Stand types

We simulated 4 common Central European tree species: European beech (*Fagus sylvatica* L.), pedunculate resp. sessile oak (*Quercus robur* L., *Qu. petraea* (Matt.) Liebl.), Scots pine (*Pinus sylvestris* L.), Norway spruce (*Picea abies* (L.) H. Karst), and, as an option for the introduction of a non-native conifer, Douglas-fir (*Pseudotsuga menziesii* (Mirbel) Franco). We offered the model to choose between monocultures of the 5 species as well as selected mixed stands. Mixed stands are tree-by-tree mixtures of the species which biophysically interact in terms of stabilization towards disturbances. The degree of stabilization depends on the species identity and the species shares in the stand. We only simulated combinations of a maximum of two species that were considered to be more resistant to disturbances when established in close mixture, i.e., mixed stands of broadleaved with softwood species (indicating different functional traits), and where the empirical survival time models of Brandl *et al.*^22^ suggest a stabilizing effect of mixture for at least one of the respective species. All other theoretically possible mixed stands can be represented by a mixture of monocultures within the optimized stand-type portfolio and were thus not explicitly simulated in the Monte-Carlo simulation. This resulted in 7 stand types that are common in the study region (see Supplementary Tab.A1– A6 for scenario-specific species compositions and rotation periods):

1. beech,
2. Douglas-fir,
3. oak,
4. pine,
5. spruce,
6. mixture of Douglas-fir and beech,
7. mixture of spruce and beech;

and, additionally, a mixture of Douglas-fir and oak (as alternative deciduous species), which is, however, not common silvicultural practice.

#### Climate

Climate conditions affect forest growth and survival probabilities in our model. The survival models of Brandl *et al.*^22^ consider a set of bioclimatic temperature and precipitation variables. We derived average climate conditions per planning unit (summarized in Supplementary Tab. A7 and Fig. A2) based on the 50 m to 50 m grid used for growth predictions. The bioclimatic variables were extracted from the WorldClim 1.4 data set^54^ for the period 2061-2080, assuming the RCP 8.5 scenario [MPI-ESM-LR].

#### Site productivity and forest growth

The forest growth and management simulation is based on yield tables developed for the study region^39^. These provide developments of volume and quadratic mean diameter over age for both standing and thinned tree compartments. Site productivity is indicated by site indices – here, the height of the mean quadratic diameter tree ($H_g$) at age 100. We predicted site indices for a 50 m to 50 m grid over the real-world forest enterprise and used the mean of all predicted site indices in a planning unit as its site productivity. This resulted in a remarkable site heterogeneity across the planning units (Supplementary Fig. A2).

We predicted site indices applying models for $H_g$ development over age under changing environmental influences^40^. They are fitted as Generalized Additive Models (GAM) separately for each species. All models are based on a modification of the Korf function, such that the predicted longitudinal patterns are always biologically sound. As predictors, the models include atmospheric influences, i.e., temperature, precipitation, and nitrogen deposition. Temperature and precipitation were available in a daily resolution and were first summed for the vegetation period, dynamically determined for each year. For nitrogen deposition, an annual sum was directly available. Then, said sums were averaged over stand life^40^. The influence of these sums on stand growth was included into the respective models in the form of spline effects. Thus, atmospheric changes over stand life can be accurately accounted for in $H_g$ predictions. Moreover, the model includes soil characteristics in the form of categorical effects for water budget index and nutrient index, provided by the public forest enterprise. Lastly, the geographic location is considered in the form of a coarse spatial smoother^40^. For future climate conditions required for the site index model (temperature sum and precipitation sum), we used the mean of three regionalization models from the ReKiEs-De core ensemble^55^ for RCP 8.5 (COSMO-CLM, WETTREG 2013, WRF), coupled with the global circulation model chosen for the bioclimatic data. Modeling of the nitrogen deposition (2061-2080) was based on data from the German Environment Agency (Umweltbundesamt)^56^.

### Biophysical disturbances

#### Stand-level disturbances

By stand-level disturbances, we refer to a stochastic disturbance event that affects only one stand – without any influence on or correlations with the neighboring stands. Similar to earlier economic simulation models accounting for forest disturbances^45,46^, we assumed that disturbances affect the entire stand, which is salvage harvested afterwards. For stand-level disturbances, we derived hazard rates^47,57^, i.e., probabilities of a stand damage in the next 10-year simulation period, based on Accelerated Failure Time survival models^22^ (Supplementary Methods A1.4.1 for details and limitations). Douglas-fir and spruce were considered to have lower hazard rates in mixed stands^22^; this stabilizing effect depended on the species shares (Supplementary Fig. A4, A5). The age-dependent and climate-dependent hazard rates were applied as the probability of stand failure in each time step of the stochastic Monte-Carlo simulation^45^. For several combinations of planning unit and species, the projected climate conditions were outside of the 99 % quantile of the observed climate conditions corresponding to the European-wide ICP Forests Level I and II data used to fit the survival models^22^. Inspired by the concept of species distribution models^58,59^, we assumed that these species hardly survive in this climate. However, we assumed that it is still possible to establish them in young stands, but with high disturbance risks. This is reflected in our model by rescaled survival probabilities until the age of 100 of only 1 % for monocultures. After disturbances, we assumed a salvage harvest with reduced wood quality and wood revenues.

#### Extreme weather events

By extreme weather events, we refer to a drastic increase of disturbance probabilities, e.g., due to a large storm event, for all stands located in a spatially explicit area that is randomly chosen in the simulation. We simulated these spatially correlated extreme weather events in addition to the stand-level disturbances.

Patterns of these rather simplified representations of extreme weather events in our model are defined by an expected number of events per 10-year period in the study region and a radius around the event’s center coordinate defining the affected area. We simulated these events by drawing a random number of events with random center coordinates evenly distributed in the study region. We scaled the damage probability to the area share of each planning unit affected by this event. In affected planning units, we assumed that stand-level hazard rates are increased as compared to those for stand-level disturbances. This increase in hazard rates is based on a rescaling of the stand-level hazard rates (see Supplementary Methods A1.4.3, Supplementary Fig. A7). The resulting extreme-event hazard rates also account for differences in stand stability depending on species, age, and species mixture. The resulting probabilities of disturbance for stands within the area affected by an extreme weather event range between 0.7 and 0.99 (see Supplementary Methods A1.4.3, Supplementary Fig. A8), which leads to a high probability of spatially clustered disturbances in the case of extreme weather events.

The number of extreme weather events per period was Poisson distributed,

9 $$\begin{aligned} P\left( N = n\right) = \frac{\lambda ^n}{n!}\exp \left( -\lambda \right) , \end{aligned}$$

with the random number of events *N* in a time period and area, its possible realizations *n*, and the parameter $\lambda$ as the expected number of extreme weather events in the study region per 10-year period (Supplementary Fig. A6).

#### Scenarios of future extreme weather events

We analyzed four main scenarios: 1) *none*, accounting for the occurrence of stand-level disturbances (assuming RCP 8.5 climate conditions) but not for extreme weather events, 2) *baseline*, representing currently observed extreme-event patterns, and scenarios with 3) *intensified* and 4) *intensive* extreme weather events (Tab. 1). The scenarios refer to the probability of stand damage due to an extreme weather event within a 10-year simulation period. Since the damage probability depends not only on the assumed patterns of the extreme weather events but also on stand characteristics, we express this probability for a spruce monoculture in an average age over the rotation period and for the average climate conditions in the region.

This damage probability due to extreme weather events $p_{dam, extr}$ can be calculated based on the probability that an extreme weather event occurs at the stand’s location (defined by $\lambda$ and the events’ influence radius $a_{extr} [km]$) and the average conditional hazard rate given for an extreme weather event at the stand’s location ${\bar{p}}_{haz, extr}$

10 $$\begin{aligned} p_{dam, extr} = 1 - \left( 1 - \frac{\pi a_{extr}^2}{A_{region}} \cdot {\bar{p}}_{haz, extr}\right) ^{\lambda }, \end{aligned}$$

with the size of the study area $A_{region}$, including a buffer zone of the extreme-event radius to avoid edge effects ($A_{region} \approx {100,000}\,km^{2}$ for the baseline scenario). Our *baseline* scenario with a 2 % damage probability within a 10-year period is in line with the assumptions of Knoke *et al.*^32^ based on past observations in Germany. In the *intensified* and *intensive* scenarios, it is increased to 4 % or 6 %, respectively.

Given one of these probability scenarios, we simulated different extreme-event patterns. We tested both a constant number of events and, thus, an increase in the probability of stand damage due to larger events, as well as an increase in only the expected number of events.

**Table 1:** Scenarios implementing different assumptions on the damage probability of a spruce stand due to an extreme weather event and patterns of those events.

Scenario		Extreme-event pattern		
		Damage probability	$\lambda$ (events per 10 years)	Radius (km)
*None*		0 %	–	–
*Baseline*		2 %	1	28.9
*Intensified:*	*Larger events*	4 %	1	40.9
	*More events*		2	28.9
*Intensive:*	*Larger events*	6 %	1	50.1
	*More events*		3.1	28.9

### Economic valuation and market risks

We conducted the growth and disturbance simulation over a period of 500 years and repeated the simulation 10,000 times per stand. The resulting harvest volumes and diameters were translated into economic cash flows. We simulated 1 stand per 1000 ha planning-unit area. We used the mean annuity of the simulated stands in one Monte-Carlo run as realization of this stand type in the planning unit. This accounted for the fact that several stands of the same type might be highly correlated but already have a lower joint economic risk than single stands. The number of simulated stands was a compromise between accounting for this effect and the computational burden in the simulation. It already reduced the distributions’ standard deviations considerably. Finally, we ran a market simulation, which considered external market fluctuations and an endogenous influence of extraordinarily high harvest volumes on the market prices.

#### Planting costs

For the planting costs (Supplementary Tab. A8), we assumed for all species except Douglas-fir that establishing a stand of the respective tree species can be achieved by a mixture of natural regeneration of existing stands in the forest enterprise and planting. We assumed full planting for the first establishment of Douglas-fir, given that it is a newly introduced species.

If a young stand ($<{100}\,a$) was disturbed, we assumed reforestation combined with an increasing share of natural regeneration over age. At ages ($\ge {100}\,a$), we assumed a species-specific mixture of natural regeneration and planting according to the German NFI following the approach suggested by Möhring *et al.*^41^.

#### Wood valuation

For the monetary valuation of thinning and harvest volumes, we applied the R package woodValuationDE^42^. It provides net revenues that depend on the tree species and quadratic mean diameter of the harvested trees. It was calibrated using Central German assortment tables^60^ and wood revenue and harvest costs functions^61^. We assumed a combination of manual and highly mechanized logging and that all stands are of moderate wood quality and easily accessible for logging operations (Supplementary Fig. A9).

#### Market risks and disturbances

External market risks, i.e., trans-regional fluctuations of market prices for wood, are implemented in the Monte-Carlo simulation by bootstrapping relative prices out of a historical price series^62^. We thus considered historic correlations in wood prices between tree species (Supplementary Tab. A9). We used a 10-year floating average (Supplementary Fig. A10) to account for the fact that we did not simulate annual fluctuations of a single stand, but rather price fluctuations for a forest enterprise. We assumed that the forest enterprise sells wood annually and collectively from different stands, resulting in lower price fluctuations for the 10-year simulation periods.

We assumed that the large forest enterprise modeled here influences the regional wood markets, as it covers a substantial share of forests in the region^31^. We used the salvage-harvest area in relation to the expected final-harvest area without any disturbances as an indicator for extraordinarily high supply due to calamities. Based on the results of Fuchs *et al.*^31^, we assumed a linear decrease in relative market prices $b_{market,rel}$ with the increasing salvage-harvest area relative to the planned final-harvest area $A_{salv,rel}$ for 10-year periods. We assumed distinct markets for softwood and deciduous species. Extraordinarily high harvest volumes of softwood species did not decrease the wood prices for deciduous species and vice versa. For softwood species *softw*, we used

11 $$\begin{aligned} b_{market,rel,softw} = 1 - A_{salv,rel,softw} \cdot 0.05, \end{aligned}$$

and for the deciduous species *dec* that were assumed to be more stable in market prices

12 $$\begin{aligned} b_{market,rel,dec} = 1 - A_{salv,rel,dec} \cdot 0.03. \end{aligned}$$

We assumed an increase of 10 % in the logging costs for $A_{salv,rel} \ge 2$ to implement higher costs due to a shortage in logging capacities.

While the market price fluctuations affected all wood sold in the region, additional logging costs and reductions in wood quality and thus revenues were applied for salvage harvests. The additional reduction in revenues due to quality losses was 15 % for deciduous and 5 % for softwood species^31^. We assumed an additional increase in harvest costs for salvage logging by 15 % for all species^31^.

### Diversity indicators

We quantified $\alpha$-diversity by two indicators at the level of individual planning units: First, the absolute number of stand types and, second, the evenness $W_p$ of the stand-type composition based on the Shannon index

13 $$\begin{aligned} W_p = -\frac{\sum _{s = 1}^{S} w_{p,s} \cdot \ln w_{p, s}}{\ln S}. \end{aligned}$$

We quantified $\beta$-diversity by the Bray Curtis dissimilarity index *B* between the stand-type composition of pairs of planning units

14 $$\begin{aligned} B_{p_1,p_2} = \frac{\sum _{s = 1}^{S} |w_{p_1,s} - w_{p_2,s} |}{2}, \end{aligned}$$

where $p_1$ and $p_2$ are two planning units. $B = 0$ indicated full similarity and $B = 1$ maximum dissimilarity. We quantified $\gamma$-diversity similar to $\alpha$-diversity, but for the overall stand-type composition of the forest enterprise as the area-weighted sum of the planning units. Additionally, we analyzed the enterprise-scale share of mixed-species stand types and the share of deciduous species as combined, simplified proxies for functional (maximum diversity at 0.5) and structural (maximum diversity at 1.0) diversity, respectively.

### Sensitivity analyses and forest structures

In addition to the extreme-event scenarios, we tested the sensitivity of our results to changes in the key input variables: Minimum and maximum hazard rate due to extreme weather events, planting costs, interest rate, internal supply effect on wood prices, reduction in wood revenues due to quality losses, quantile of the $CV\!aR$, and our assumption on the survival probabilities when the climate conditions lead to an extrapolation of the survival functions.

In order to estimate the consequences of economic adaptation of stand-type allocations to extreme weather events, we estimated the expected age structure, disturbed area, standing volume, mean tree diameter, and harvest volume for the optimal solutions and compared them between the extreme-event scenarios and, where data was available, with the real forests in the region according to the German NFI 2012^36^. To estimate these measures for our model output, we derived age-dependent probabilities of final harvest and salvage harvest due to stand-level disturbances or extreme weather events for each species and stand type based on the 10,000 Monte-Carlo runs of stand development over 500 years. This provided the probability that a stand would be harvested at a certain age. We derived the expectations for the mentioned measures analogously to deterministic estimates of the expected annuity as a mean weighted by the disturbance probability^46,47^. These estimations can be interpreted as long-term mean values in the forest enterprise under optimized management.

## Results

### Economically optimal stand-type diversification was higher under bottom-up planning (*H1*)

**Figure 3:**
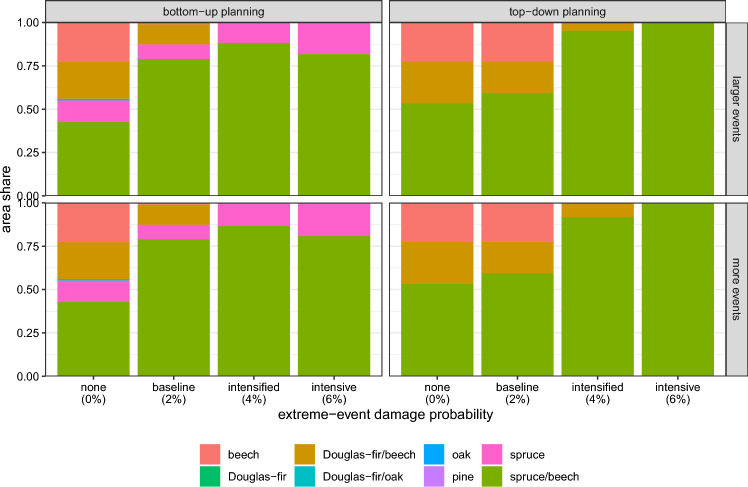
Optimized stand-type composition of the forest enterprise, as sum of all planning units, reflecting $\gamma$-diversity. Optimal compositions were derived under top-down and bottom-up planning (panel columns) and different extreme-event scenarios, defined by a probability of stand damage within 10 years (horizontal axis) and an increase in the size or number of events (panel rows).

**Figure 4:**
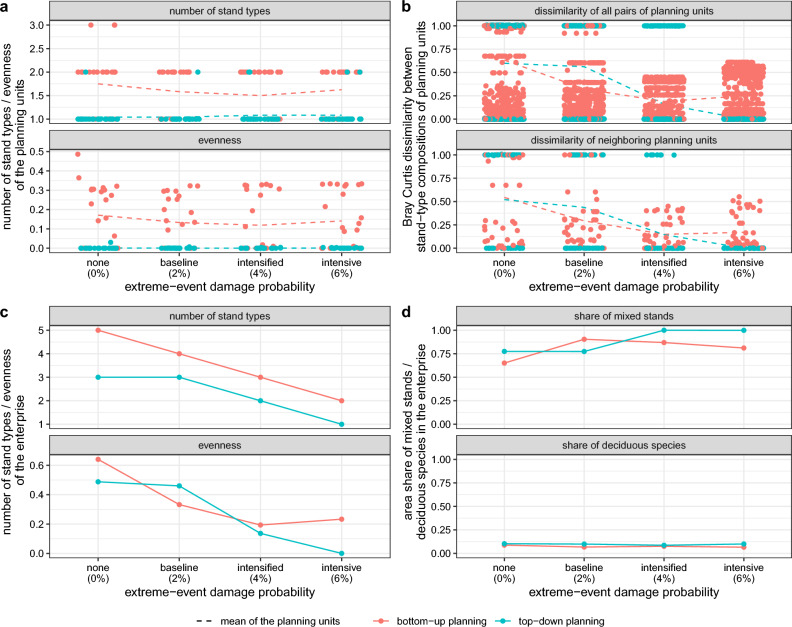
Diversification strategies under economically optimal stand-type allocation. Indicators for $\alpha$ (**a**) $\beta$ (**b**) $\gamma$ (**c**), structural and functional diversity (**d**) of the optimal stand-type allocation, comparing bottom-up and top-down planning under extreme-event scenarios defined by a damage probability (horizontal axis). We show only the results for increasing damage probabilities due to more extreme weather events. The results for larger events were similar (Supplementary Fig. A11). Each point in (**a**) represents one of the 24 planning units, and in (**b**), one pair of planning units. (**c**,**d**) refer to the entire enterprise as aggregated planning units. Bray Curtis dissimilarity index (**b**) describes $\beta$-diversity; a value of 0 indicates that the stand-type composition of two planning units is identical, while a value close to 1 indicates high dissimilarity.

The diversity of the optimal stand-type composition of the enterprise and the individual planning units was higher under bottom-up planning than under top-down planning when excluding extreme weather events. The most abundant stand type in the optimal portfolio of the enterprise, aggregating all planning units, was the spruce-beech mixture with 43 % under bottom-up planning and 53 % under top-down planning (scenario *none 0 %* in Fig. 3). Apart from this mixture, beech monocultures and Douglas-fir-beech mixtures were the most abundant stand types. Under bottom-up planning, spruce monocultures and a small area share of Douglas-fir-oak mixtures were additionally selected. This resulted in 5 stand types under bottom-up planning as compared to 3 stand types under top-down planning as indicator for $\gamma$-diversity (scenario *none 0 %* in Fig. 4c). Regarding the spatial diversification strategies, we found that under top-down planning, the model diversified only across the planning units ($\beta$-diversity, Fig. 4b). This means that only one stand type was selected in 23 of 24 planning units (turquoise points in Fig. 4a) leading to very low $\alpha$-diversity. In contrast, bottom-up planning diversified the stand-type composition within the planning units ($\alpha$-diversity, red points in Fig. 4a). As a result, stand-type composition evenness in the planning units was higher under bottom-up than top-down planning (Fig. 4a). However, at enterprise level, the structural diversity in terms of the share of mixed stands and the functional diversity in terms of the share of deciduous species (maximum diversity at 50 %) were higher under top-down planning (Fig. 4d). The aggregated stand-type composition of bottom-up planning differed from the top-down optimum as different diversification strategies were optimal.

Site conditions appeared to explain differences in the stand-type composition between the planning units ($\beta$-diversity), but their influence did not considerably differ between the planning perspectives. Beech monocultures and mixed stands of Douglas-fir with beech were more likely selected on colder and wetter sites (Supplementary Fig. A15, A16), while gradients of the site productivity and latitude (Supplementary Fig. A13, A14) had no clear influence under our purely economic objective function. Site heterogeneity in climate variables, which influenced survival probabilities and growth, was thus a key driver of $\beta$-diversity.

### Extreme weather events homogenized the stand-type composition (*H2*)

Our results do not support *H2* that diversifying the stand-type allocation is optimal when the intensity of extreme weather events increases. Rather, we observed a trend towards homogenization for both planning perspectives. The model focused on spruce-beech mixtures under increasing extreme weather events (scenarios *baseline*, *intensified*, and *intensive* in Fig. 3). Under top-down planning, the model additionally selected decreasing shares of beech monocultures and mixtures of Douglas-fir with beech. In contrast, the model introduced increasing shares of spruce monocultures under bottom-up planning. Regarding the spatial diversification strategies, we found a general trend towards homogenization for $\beta$ and $\gamma$-diversity (Fig. 4b,c) when extreme weather events were introduced to the model. The general patterns of homogenization and the dominance of spruce-dominated stand types were considerably stable, independent of whether extreme-event damage probability increased due to an increase in the number or in the size of events (Fig. 3).

We observed an influence of the site conditions on the bottom-up allocation of stand types to planning units under all extreme-event scenarios. Spruce monocultures, the stand type with the lowest planting costs in our study, were most likely introduced at sites with unfavorable climate and a low site index of beech (Supplementary Fig. A13, A15, A16). On sites with more favorable climate conditions and site productivity, the model selected spruce-beech mixtures. This indicates that under increasing extreme weather events, investments in more stable stands by introducing 10 % beech in spruce stands were more reasonable on sites with comparably low biophysical risks and high productivity of beech.

### Economically optimized stand-type allocation did not buffer economic consequences of extreme weather events (*H3*)

**Figure 5:**
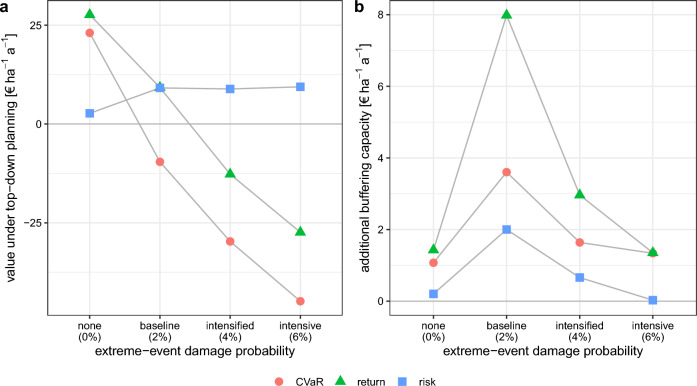
Economic performance of the forest enterprise under the optimized stand-type allocations from the top-down perspective (**a**) and additional buffering capacity of top-down planning, i.e., top-down value minus bottom-up value (**b**). $CV\!aR$, the Conditional Value at Risk (10 %-quantile) is the maximized objective function; return refers to the expected annuity (annual economic return) and risk to the standard deviation of the annuity in a Monte-Carlo simulation with 10,000 repetitions. The performance is shown for different extreme-event scenarios defined by a damage probability (horizontal axis) assuming an increase in the number of events. Results for an increase in the size of the events were similar, see Supplementary Figure A17.

In our simulations, adapting the stand-type allocation in the forest enterprise did not fully compensate for the adverse economic consequences of the simulated extreme weather events, irrespective of the planning perspective. When comparing the economic performance of the forest enterprise under the top-down optimized stand-type allocation, the $CV\!aR$ decreased from 23 to -45 € ${{\text{ha}}^{-1}{\text{a}}^{-1}}$ in the intensive extreme-event scenario (Fig. 5a). The return, as expected annuity, decreased from 28 to -27 € ${{{}}}{{\text{ha}}^{-1}{\text{a}}^{-1}}$. The risk, as standard deviation of the annuity, increased from 3 to 9 € ${\text{ha}}^{-1}{\text{a}}^{-1}$. $CV\!aR$ was negative in all extreme-event scenarios and return was negative under intensified and intensive extreme weather events (Fig. 5a), despite economically adapted stand-type allocation. This explains the finding that the model chose increasing shares of spruce-dominated stand types, as the expected return did not compensate for the risks of high investment costs for other species (see planting costs in Supplementary Tab. A8).

Differences in the economic performance of the enterprise between the planning perspectives were small. The additional buffering capacity of the more flexible top-down planning across planning units was at maximum about 3.6 € ${{\text{ha}}^{-1}{\text{a}}^{-1}}$ in $CV\!aR$ and 8 € ${{\text{ha}}^{-1}{\text{a}}^{-1}}$ in return (Fig. 5b). The higher return and $CV\!aR$ at the enterprise level under top-down planning were partly realized at the cost of higher risks of individual planning units (Supplementary Fig. A18). Diversification across the region allowed for accepting higher risks. However, this effect was limited due to a common focus on stand types with low investment costs. In turn, the chance for better-performing simulations in terms of annual return increased under top-down as compared to bottom-up planning (Supplementary Fig. A19).

### Economic stand-type allocations transformed future forest structures under extreme weather events (*H4*)

Here, we restricted our perspective to economically rational decision-making, focusing on wood production income as a key ecosystem service. Even though we considered economic risks in decision-making, we disregarded potential consequences on the resulting forest structures (e.g., age structure, standing volume, and disturbed area). To illustrate the order of magnitude of these consequences, we show the respective model results and the corresponding information on the real-world forests in this region according to the last German forest inventory (NFI 2012^36^). It is important to note that we refrained from including the current species distribution and forest structures in our approach and focused on a purely economic objective. Differences between stylized optimization results and NFI are, thus, hypothetical.

**Figure 6:**
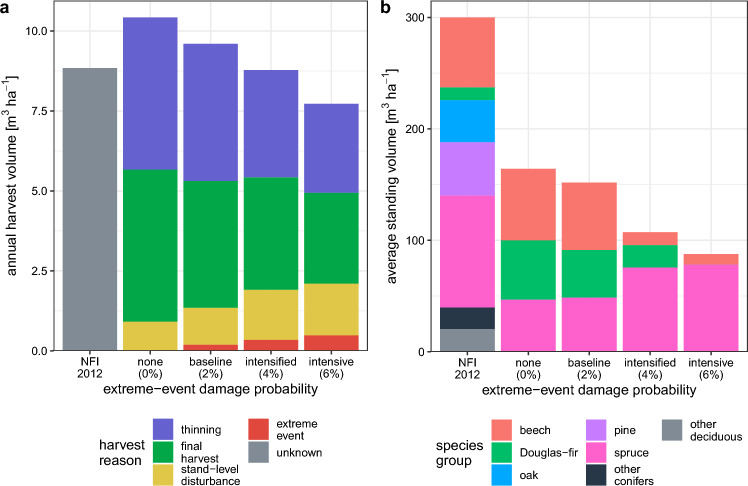
Consequences of the likely stand-type allocation under the risk-averse economic objective function for the region’s forests. Comparison of the average of the simulated annual harvest volume (**a**) and standing volume (**b**) to the German NFI 2012^36^ (left bars). The values refer to top-down planning and are shown for increasing damage probabilities due to an increase in the number of extreme weather events (horizontal axis). Results for bottom-up planning and an increase in the size of the events are shown in Supplementary Figures A21–A23.

The economically optimal stand-type allocation led to an increase in the annually disturbed area of the forest enterprise. In the scenario with only stand-level disturbances, stands were harvested each year at about 1.3 % of the enterprise’s area. About 59 % of the harvested area were salvage fellings due to disturbances (Supplementary Fig. A26). Under intensive extreme weather events, the annually harvested area increased to about 2 %, with 80 % thereof as salvage harvests. The corresponding annual harvest volume in the scenario without extreme weather events was higher than the actual volume estimated in the NFI for the study region (Fig. 6a). However, the modeled harvest volume decreased with increasing extreme weather events and the share of salvage-harvest volume increased from 9 to 27 %, despite risk-averse economic adaptation. Since disturbances often occurred also in young stands under the extreme-event scenarios, the share of salvage harvest volume was lower than the area share of salvage harvests. Harvest volumes from thinnings and the higher volumes in the case that a stand reached the planned rotation age compensated for parts of the losses as compared to the pure area shares.

Under stand-level disturbances and top-down planning, the average standing volume was 187 $\text{m}^{3}\,\text{ha}^{-1}$, which is about 63 % of the volume observed in the NFI (Fig. 6b). With an increasing number of extreme weather events, the standing volume of the simulated forests further decreased to 88 $\text{m}^{3}\,\text{ha}^{-1}$. The standing volume of beech decreased disproportionately, as the average age of beech decreased under extreme weather events from 57 to 37 years (Supplementary Fig. A24). We found a considerable loss of older forests (age $>70$), particularly as compared to the current forests in the region (Supplementary Fig. A25). This also led to a reduction in the quadratic mean diameter (Supplementary Fig. A27). The changes in forest structures and attributes as compared to the NFI suggest that the economic decision-making behavior simulated here may also impact other ecosystem services in addition to wood production.

Regarding species diversity, we compared the simulations with NFI data using tree-species groups (the five species in our study plus other conifers and other deciduous). In contrast to the region’s actual species composition, our optimization selected only 2-4 of the 5 species included in our model with considerably lower evenness (Supplementary Fig. A29). These results transferred well to individual planning units. Most of the planning units were composed of 2 species groups in our simulations, while the median in the NFI was 6 (Supplementary Fig. A30). Our results show that, although explicitly accounting for economic risks, optimizing the stand-type allocation did not result in biophysically stable forests, nor in diverse forests with old age classes and high standing volumes. This trend was substantially enhanced by extreme weather events, leading to a remarkable transition of forest structures.

### Planting costs were a key driver of economic stand-type allocations (*sensitivity analysis*)

**Figure 7:**
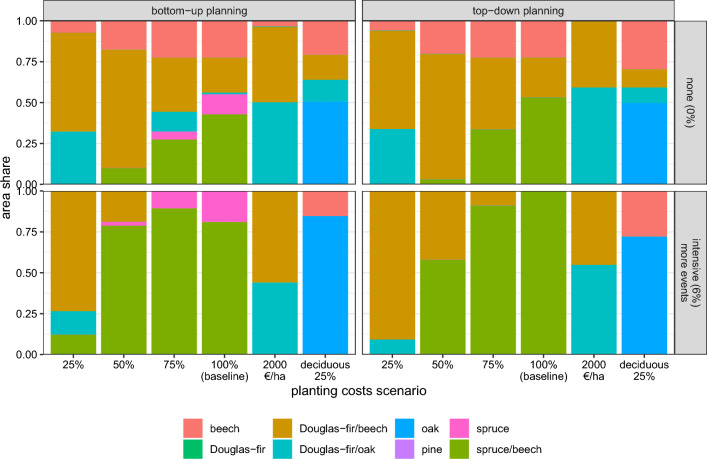
Sensitivity of the enterprise’s optimal stand-type composition to changes in the planting costs, simulated under different planning perspectives and extreme-event probabilities. The planting costs were reduced to 25 %, 50 % and 75 % as compared to the baseline scenario (Tab. A8); in addition, we tested equal planting costs of 2000 € ${{\text{ha}}^{-1}}$ for all species; in *deciduous: 25 %*, we reduced only the planting costs of deciduous species, reflecting a typical type of funding policy in the study region.

The focus on spruce-dominated stand types was largely driven by low planting costs and early returns of spruce. In the sensitivity analysis of planting costs, mixed stands with Douglas-fir dominated the stand-type portfolio in the absence of extreme weather events if the absolute additional costs for establishing Douglas-fir were lower, i.e., when planting costs for all species were reduced to $\le {50}\,\%$ or set to 2000 € ${{\text{ha}}^{-1}}$ (Fig. 7, upper panels). The general trend of $\alpha$-, and also (less pronounced) $\gamma$-diversity, being higher under bottom-up than top-down planning was only weakly affected by altered planting costs (Fig. 7, Supplementary A38). Under extreme weather events, the dominance of spruce-dominated stand types persisted when we reduced the planting costs (Fig. 7, lower panels). However, stand types with the more expensive, but also more stable, Douglas-fir became dominant when Douglas-fir planting costs were considerably reduced (scenarios *25 %* and *2000* *€* $\it {{\text{ha}}^{-1}}$), while reducing only the planting costs of deciduous species favored beech and the most stable species, oak (scenario *deciduous 25 %*). Lowering the planting costs was able to compensate for large parts of the reduction in $CV\!aR$ under the intensive extreme-event scenario as compared to the stand-level disturbance scenario (Supplementary Fig. A39). The sensitivity analysis thus underpinned that economic species selection and its buffering capacity depend on planting costs. Notably, the general pattern of homogenization under extreme weather events was remarkably stable.

A lower interest rate, i.e., time preference, had similar consequences as reducing planting costs. It favored Douglas-fir and oak, while a higher interest rate (3 %) favored spruce monocultures, i.e., the stand type with the lowest planting costs (Supplementary Fig. A40). If the reduction of market prices due to high salvage supplies was stronger, the model tended to prefer the more stable spruce-beech mixture, while lower reductions increased the share of Douglas-fir and spruce monocultures (Supplementary Fig. A43) – but, again, the trend towards homogenization of the stand-type composition under extreme weather events was only weakly affected by these changes in model parameters.

The general patterns of homogenization of the economically optimal stand-type portfolio and a decline in $CV\!aR$, despite economic adaptation of the stand-type allocation, were only weakly affected by a wide range of scenarios for extreme-event patterns and related model parameters tested here (Figs. 3, 5, Supplementary Fig. A31, A33, A34, A36, A52, A54).

## Discussion

### Stand-type diversification under different planning perspectives and spatial heterogeneity (*H1*)

Referring back to *H1*, stand-type allocation was more diverse under bottom-up than top-down planning. Although the diversification strategies ($\alpha$ vs. $\beta$-diversity) differed between the planning perspectives, the model selected mainly stand types with spruce and beech for both cases, leading to large similarities in the overall species shares.

Our findings support the common hypothesis that diversifying tree-species or stand-type composition is economically reasonable under climate-driven disturbances^4,24,63^. Our study extends earlier studies focusing on compositional diversity to diversification strategies at larger spatial scales. We showed that regionally coordinated top-down allocation of stand types is advantageous in terms of spatial flexibility. Economic top-down adaptation focused on diversification across ($\beta$-diversity) rather than within planning units ($\alpha$-diversity). In our example, the corresponding additional buffering capacity as compared to bottom-up planning was limited, as spatially homogeneous socio-economic drivers, e.g., the assumed establishment costs, were more relevant than heterogeneous site conditions. Our results, therefore, only show a limited potential of top-down policies for regional coordination of stand-type allocation to reduce adverse economic consequences of climate-driven disturbances.

Site heterogeneity clearly influenced the stand-type allocation to specific sites. However, its general influence was limited in our model. First, this is due to the purely economic objective function. For example, oak as more drought-resistant species^64^ might be chosen by forest managers for hot and dry sites, but was omitted by the model due to its high assumed establishment costs and long production periods. Second, we used a relatively coarse resolution of site heterogeneity with homogeneous site indices and climate conditions in each planning unit. While our study covered considerable gradients, we did not account for rarer sites, such as moist sites, where, e.g., alder would be a suitable option. We would expect a stronger influence of site heterogeneity on tree-species allocation if the disturbance models accounted not only for climate variables but also, e.g., the consequences of exposition for storm risks^65^. While more detailed models exist for individual disturbance agents^66,67^, they have rarely been combined in one general disturbance model as our approach would require. Third, we did not account for spatial heterogeneity in socio-economic variables, which may have a stronger influence than site heterogeneity^37^. Local differences in the demand for ecosystem services or different expectations and risk attitudes of decision-makers would likely increase $\beta$-diversity in our model. While out of the scope of our study, heterogeneous decision-makers have already been integrated in robust multi-criteria optimizations^68,69^. This approach, which is less data-demanding than agent-based models, could be a future extension of our model.

Accordingly, stand-type richness was low in our economic optimization, which did not consider guidelines for site-specific tree-species selection based on ecograms^70^, as commonly applied in the study region. The model predominantly selected stand types with spruce and beech, which are the two most common species in public forests of the study region (25.3 % spruce and 20.5 % beech^36^). Pine (18.4 %) and oak (12.9 %^36^) had no notable share in the optimized portfolio. In line with previous economic studies^24,45,71^, the model preferred spruce and Douglas-fir as highly productive conifers and combined them with a stabilizing deciduous species. In contrast, studies aiming for multiple ecosystem services suggest more diverse stand-type compositions^5^. A multi-criteria objective function would likely also lead to higher differences between locally and regionally coordinated solutions. This is because the additional spatial flexibility of regional top-down planning would likely allow for better balancing of the different objectives. However, the small economic disadvantages of top-down compared to bottom-up planning from the local forest owner’s perspective in our study would probably increase under regionally coordinated multi-objective planning. In contrast to our purely economic result, a multiple-objective approach may thus raise a need for spatially targeted incentives. Our study provides an efficient spatial heuristic, which could be combined with robust multi-objective optimization^72,73^ to estimate efficient incentives for maintaining multifunctional forests under increasing disturbances.

To summarize, our new methodological advances in scaling up of tree-species portfolio models allowed us to show that the regionally coordinated optimum differs from the sum of the optimal solutions for the local planning units. Even if the effects were limited for our data example, spatial heterogeneity under regional-level planning seems to promote diversification beyond a pure portfolio effect. This suggests that commonly applied models for stand-level portfolio optimization tend to promote local compositional diversity, while diversification across a region may lead to better economic performances.

### Stand-type diversification under extreme weather events (*H2*)

We could not confirm *H2*. Instead of higher stand-type diversity within the local planning units, increasing extreme weather events led to homogenization of stand types within and between the planning units. We designed the model to reflect adaptation of stand-type allocations to extreme-event scenarios in order to maintain an economic objective function. However, we observed a homogenization with a clear dominance of vulnerable and highly debated spruce-dominated stand types under our extreme-event scenarios and model assumptions. This better reflects decisions expected from non-adaptive, rather than trend-adaptive or proactive, decision-makers^38^. By selecting stand types with low planting costs, the model focused on reducing investment risks rather than aiming for high expected returns. If all options lead to negative returns and high risks, the species with the lowest planting costs and early positive net revenues is chosen, reflecting a fatalistic behavior. It implemented a strategy focusing on minimizing today’s costs rather than maintaining a high expected economic performance in the future. In our example, these conditions were best met by spruce. This explains the counter-intuitive result of increasing shares of this species despite its low survival probability under climate change. The often selected mixture with a small share of beech, however, reflects an investment in slightly reduced biophysical risks. The sensitivity analysis confirmed planting costs as a key driver in our model, which is in line with findings by Neuner and Knoke^71^ and Paul *et al.*^45^. Homogenization of the stand-type portfolio was particularly pronounced, as the $CV\!aR$ focuses on the worst outcomes, i.e., disturbances in several young stands simultaneously. Such situations were more likely under the newly introduced spatiotemporal correlations due to extreme weather events. The economic approach starting from bare land may also slightly exacerbate the negative consequences of extreme weather events occurring early in the simulation. Future studies modeling a dynamic transition to adapted stand-type allocations could test the influence of initial age-class distributions. The observed effect was stronger under bottom-up planning, which resulted in admixtures of spruce monocultures instead of beech monocultures or Douglas-fir-beech mixtures. Top-down planning invested in higher shares of these more stable, but also more expensive, stand types. Losses in one planning unit could be compensated by returns in other planning units that were not affected by the same event. Although these findings were enhanced by risk-averse decision behavior, we found the general patterns to be stable under different degrees of risk aversion. The trend towards homogenization focusing on spruce-beech mixtures occurred even under a risk-neutral perspective (Supplementary Fig. A12).

In a real forest enterprise, such fatalistic decision behavior would probably still lead to more diverse stand-type compositions than in our simulations. Other low-investment options for stand establishment would be uncontrolled natural regeneration of the previous stand, likely not adapted to altered disturbance patterns, combined with spontaneous regeneration of other species. In particular, low investment options with natural regeneration of pioneer species, such as silver birch (*Betula pendula* Roth)^74^, are attractive with regard to the juvenile growth rates^75^ and low costs of establishment if seed trees are available in the neighborhood^76^. Further developing regeneration, growth, disturbance, and economic models for such situations is a promising field for future research.

The fatalistic behavior of the model is furthermore driven by the rather low probabilities of tree survival under extreme weather events, assumed here as compared to previous studies^24,45^. When focusing only on stand-level disturbances according to Brandl *et al.*^22^, we found a more diverse stand-type composition than under additional extreme weather events. As the applied survival functions cover all disturbance intensities, historic extremes are part of the average survival probabilities. However, the data refers only to a short period from 2010 to 2017. Large hot-drought events, such as those observed in 2018-2020^27,28^, are not included, but are expected to increase under climate change^77^. It is thus likely that adding extreme weather events to these functions does not overestimate the disturbance risk. Species distribution models even suggest that under the predicted climate conditions in the region, spruce, in particular, will be outside the climatic niche in the period 2070-2100^59^. If it should no longer be possible to establish these tree species, even at higher costs, the situation could be even more pessimistic than in our approach with artificially reduced survival probabilities under such climate conditions. However, we observed a reduction in stand-type and species richness under low as well as high extreme-event probabilities, suggesting that the general pattern of stand-type homogenization under increasing extreme weather events only weakly depends on detailed assumptions on future extreme-event patterns.

We thus conclude that extreme weather events likely jeopardize provisioning services and the willingness of production-oriented forest enterprises to invest in resistant stand types, e.g., oak or pine. Consequently, species richness would likely be narrowed to low-investment options and fast-growing species under increasing extreme weather events.

### Economic buffering capacity of adapted stand-type allocation (*H3*)

Referring back to *H3*, adapting the stand-type allocation did not fully compensate for the economic consequences of climate change in our model. The additional spatial scale of diversification under top-down planning provided only a limited additional buffering capacity regarding the economic consequences of extreme weather events.

In line with stand-level models by Paul *et al.*^45^ or Friedrich *et al.*^11^ (excluding Douglas-fir) and in contrast to Fuchs *et al.*^24^ (including Douglas-fir), the $CV\!aR$ as well as the expected return decreased with increasing disturbances even though forest management was adapted. This finding was stable in the sensitivity analyses. Providing an economic analogy to the ecological concept of $\alpha$ and $\beta$-diversity in the context of tree-species allocation^35^, our results show that the additional spatial scale of diversification across planning units provides a higher buffering capacity towards consequences of disturbances. However, in our economic example and at this coarse spatial resolution, the effect was smaller than expected. The reduction in $CV\!aR$ in bottom-up as compared to top-down planning was limited under extreme weather events and only gained relevance at high levels of risk aversion (1 %-quantile). The difference would likely increase if spatial heterogeneity was more pronounced or more ecosystem services were considered.

As a novel component, we introduced an endogenous market effect of a regional wood oversupply in the case of extreme weather events^31^. The introduced spatiotemporal correlations of salvage harvests could be even stronger if, for instance, the spread of forest pests such as bark beetles were explicitly simulated. Infestations would directly affect the neighboring stands. This spatial connectivity between stands, disregarded here, could be simulated, e.g., by cellular automata^78^ and would allow for assessing adapted stand-type allocation as a pest-management strategy. This would, however, drastically increase computational complexity, thus restricting the number of scenarios, planning units, and Monte-Carlo repetitions.

Our methodological approach may slightly underestimate the economic adaptation potential of forest management to extreme weather events. To reduce the computational complexity, we used a rather static approach, where the decision-maker can only choose among pre-defined stand types under a constant climate scenario. A more dynamic representation, e.g., by adjusting species composition and rotation periods simultaneously with the allocation of stand types to planning units may increase the ability to adapt to changing expectations on disturbance regimes and market prices. However, the differences to a more dynamic decision-making are likely small. We pre-optimized the stand-types’ management regimes, i.e., rotation periods and species shares in mixed stands were constant in the main optimization step. In general, rotations that are optimized for each stand separately are optimal in a larger regional context, if the price is exogenous. Equivalently, if there is a linear utility function, the stands can be optimized separately^79^. Stochastic disturbance risks also lead to a fixed optimal rotation that can be solved in a pre-optimization step^80^. The same applies to the optimization of the species composition in mixed-species stands. However, randomly fluctuating prices could justify endogenous harvest decisions that react to price realizations. Choosing different compositions of species in mixed stands across planning units would benefit from a slightly higher resistance in mixed stands as compared to adjusting the species shares by including the monoculture stand type of one of the species. Including monocultures, however, seldom occurred in our top-down optimization and the effect on the objective function would be rather limited. Using pre-optimized management regimes to reduce computational burden should, thus, not notably affect the optimal stand-type allocation in the later stage optimization. It will, however, underestimate the profitability of forestry as, for example, the decision-makers cannot utilize high-price realizations for harvesting and low-price realizations for not harvesting. Future studies could account for possible advantages of a more time-dynamic decision-making, for example, based on dynamic programming^81,82^ or Bayesian updating of beliefs^83^. However, the positive effect of dynamic decision-making on profitability would depend on the decision-maker’s ability to accurately assess the expected future disturbance regimes and wood prices. The resulting increase in profitability would most likely not compensate for the severe economic losses found in our study.

Our objective function balanced long-term economic risks and returns. Even under the long-term perspective, planting costs were a key driver of the simulated decisions. This resembles practical decision-making in the context of large disturbances, as short-term liquidity and financing reforestation have been found to be a relevant concern of forest managers after large disturbances^13^. However, $CV\!aR$ falls short in capturing the full importance of liquidity after large disturbances, as the underlying *LEV* assumes a perfect capital market. Considering liquidity and explicitly addressing the financing problem in our model would likely amplify the observed trend to avoid investments in planting costs and the associated economic risks. Knoke *et al.*^84^ suggested that continuous-cover forestry, i.e., age-class or vertical diversity at the stand-level, provides a faster economic recovery after disturbances, which mitigates the economic short-term consequences. The positive effect of continuous-cover forestry, however, would need to be very pronounced to reverse the trend towards homogenization under spatially correlated extreme weather events.

Our spatially explicit modeling approach allowed us to test diversification strategies under spatially correlated extreme weather events. From the results, we conclude that the economic buffering capacity of diversification across a forest region is not sufficient to compensate for the economic consequences of climate-driven increases in extreme weather events. The limited economic buffering capacity makes regime shifts in terms of species compositions and stand structures more likely in future forest management.

### Consequences for other ecosystem services (*H4*)

Under extreme weather events and avoidance of investment risks, the forests were less diverse and the model accepted larger disturbance areas. This led to changes in the forest structures and attributes as compared to the forests in the region according to the last NFI. These changes likely impact the forests’ provision of other ecosystem services^44^. For instance, high-yield conifers are in general beneficial for carbon sequestration^85^, but standing wood biomass and harvest volumes (wood products and substitution) decreased under our extreme-event scenarios. From a biodiversity perspective, not only were the species portfolios homogenized, but also the disturbance area in production-oriented forests increased. While a limited number of disturbances may be generally positive from an ecological perspective, the subsequent salvage harvests may offset positive effects for biodiversity^86^.

To conclude, a society seeking diverse, resilient forests with species of different functional and response traits that provide a range of ecosystem services cannot assume that forest enterprises invest in such stands solely driven by internal economic rationales related to wood production. Our results point towards a need for incentives for establishing climate-resilient stands. Reducing investment risk by subsidizing planting costs appears to be a promising approach.

## Conclusion

We scaled up economic tree-species portfolio optimization to a forest region with spatially explicit site heterogeneity and multiple planning units. These methodological advances allowed us to assess the consequences of spatially correlated extreme weather events compared to independent stand-level disturbances for future forest structures under different planning perspectives. Although site heterogeneity and planning perspectives had a limited influence in our data example, we captured new mechanisms not included in current stand-level models addressing climate adaptation of forests. The results suggest that stand-level studies tend to underestimate the economic potential of diversification strategies at larger spatial scales that are relevant for large private or public forest enterprises and forest policy design. Future studies should account for spatial and temporal correlations between biophysical stand development and socio-economic consequences at a regional scale. Promising aspects for future research extending our approach are 1) applying more site-sensitive disturbance models and a higher spatial resolution, 2) integrating spatial heterogeneity in socio-economic variables, including demand for multiple ecosystem functions, 3) coupling our model with more elaborate market simulations, 4) accounting for economic short- and mid-term consequences of disturbances, 5) comparing economic adaptation potentials of species and structural diversity.

Three main effects of extreme weather events on stand-type allocations were particularly relevant for forest management and policy: 1) Increasing extreme weather events resulted in a homogenization at different scales of stand-type diversification ($\alpha$-, $\beta$-, $\gamma$-diversity) under economic adaptation. 2) High investment risks fostered fatalistic decisions: Investment costs and early revenues became more important for economic decision-making under extreme weather events and outweighed the avoidance of stand failure. Stand failure was accepted to avoid higher investment risks required to establish more stable stand types. 3) The positive economic portfolio effect of tree-species diversification was reduced under extreme weather events, even when considering risk-averse decision-makers and a long-term planning perspective. These general findings were consistent across different scenarios and sensitivity analyses, suggesting that they are transferable to other regions with similar biological and socio-economic forest production systems.

Our results suggest that the common hypothesis stating that high species diversity at all spatial scales enhances both ecological and economic stability needs to be carefully reconsidered. A high ecological stability may not necessarily buffer economic consequences of extreme weather events. For society and policy, this result is highly relevant for designing efficient incentives that foster the establishment of stable forests that provide multiple ecosystem services.

### Supplementary Information

Supplementary Information.

## Data Availability

The model’s input data are available in the Supplement. The underlying geometries, the site information, and the time series of wood prices are operational data of the public forest enterprises and cannot be made publicly available. The model code is available from the corresponding author upon reasonable request.
