# Supplementary material for: Severe and frequent extreme weather events undermine economic adaptation gains of tree-species diversification
Source: Sci Rep. 2024 Jan 25;14:2140. doi: 10.1038/s41598-024-52290-2 (PMC10810831; doi:10.1038/s41598-024-52290-2)
Supplement: Supplementary file 1 — Supplementary Information. [file 41598_2024_52290_MOESM1_ESM.pdf]

Electronic Supplementary Material

Electronic supplementary material to
Severe and frequent extreme weather events undermine economic adaptation gains of tree-species diversification by
Jasper M. Fuchs, Kai Husmann, Jan Schick, Matthias Albert, Jussi Lintunen, and Carola Paul

A1 Supplementary methods

A1.1 Stand types

A1.1.1 Pre-optimization of management regimes

Pre-optimization of rotation periods: We pre-optimized the rotation periods of the species accounting for the growth and climate conditions in the different planning units. In the case of mixed stands, we considered the stabilizing effect of mixture against biophysical disturbances (see Section *Stand-level disturbances*[1] in the main text). Applying a deterministic approach, the model searches for the rotation period T in 10-year age steps that maximizes the expected annual return (annuity) $\mathbb{E}[R]$,

$$\max_{T_{p,s,w_s}\in[10,20,\dots,190,200]} \mathbb{E}\left[R_{T_{p,s,w_s},p,s,w_s}\right], \tag{A1}$$

with the planning unit of the forest enterprise p, the species s, and the area share of the species in the stand type $w_s = 0.1, 0.2, \dots, 0.9, 1$. $\mathbb{E}[R]$ is calculated as the average of the annuities of all possible scenarios of stand-level disturbances or a planned final harvest weighted by their occurrence probabilities[2;3],

$$\mathbb{E}\left[R_{T_{p,s,w_s},p,s,w_s}\right] = \sum_{t=0,10,\dots,T_{p,s,w_s}} H_{p,s,w_s}(t)\,R_{p,s,w_s}(t, salvage) + S_{p,s,w_s}(T_{p,s,w_s})\,R_{p,s,w_s}(T_{p,s,w_s}, planned.harvest), \tag{A2}$$

with the unconditional probability of a disturbance at age t, $H_{p,s,w_s}(t) = S_{p,s,w_s}(t-10) - S_{p,s,w_s}(t)$ (see survival model in Section A1.4.1), the annuity in case of a salvage harvest at age t, $R_{p,s,w_s}(t, salvage)$, the probability to reach the rotation period, $S_{p,s,w_s}(T_{p,s,w_s})$, and the annuity in the case of a rotation with a planned final harvest without disturbance, $R_{p,s,w_s}(T_{p,s,w_s}, planned.harvest)$. For each species and share in a potential mixed stand, the rotation period with the highest expected annuity was applied in the following Monte-Carlo simulation. We are aware that the objective function chosen here, the expected annuity, deviates from the objective function used in the optimization problems of later stages, i.e., the Conditional Value at Risk *CVaR*. This allowed us to apply the deterministic approach, which considerably reduced the computational burden in our model. Pre-optimizing the rotation periods guaranteed that we compare optimal management regimes for the different stand types. However, changes in the rotation period have a limited impact on *CVaR*, as *CVaR* is the expected value of the $\alpha = 10\%$ worst outcomes, i.e., simulations where the stand was disturbed far before the planned rotation age.

Pre-optimization of species shares in mixed stands: Based on the results of the Monte-Carlo simulation of all monocultures and possible species mixtures (in 10 %-steps of area shares), the model searches for the species compositions in mixed stands that maximize the economic objective function *CVaR* under the consideration of the scenario's disturbance regime and market fluctuations. The species composition of a stand type is the same for all planning units $p = 1, 2, \dots, P$ of the forest enterprise. We simulated mixtures of a conifer species s_c with a deciduous species s_d and the area shares of these species in the stand $w_{s_c} \in [0.1, 0.2, \dots, 0.8, 0.9]$ and $w_{s_d} = 1 - w_{s_c}$, respectively. We thus maximized the *CVaR* dependent on the area share of the conifer,

$$\max_{w_{s_c}} CVaR_\alpha(w_{s_c}). \tag{A3}$$

The simulated $CVaR_\alpha(w_{s_c})$ is based on the empirical joint annuity distribution, $F_{R(w_{s_c}, w_{s_d})}$, consisting of $n = 10000$ realizations of the Monte-Carlo simulation with $i = 1, 2, \dots, n$ repetitions. The joint annuity distribution accounts for correlations in the annuity of the two species due to disturbances or market fluctuations. A realization i of this annuity $\left[€\,\mathrm{ha}^{-1}\,\mathrm{a}^{-1}\right]$ is

$$R_i(w_{s_c}) = \sum_{p=1}^{P} \frac{A_p}{A}\left(w_{s_c} R_{p,s_c,w_{s_c},i} + (1-w_{s_c})\,R_{p,s_d,1-w_{s_c},i}\right), \tag{A4}$$

with the area of the planning unit p, A_p, the area of the entire enterprise, $A = \sum_p A_p$, the realization of the annuity of the conifer, $R_{p,s_c,w_{s_c},i}$, and that of the deciduous species, $R_{p,s_c,1-w_{s_d},i}$. The calculation of the *CVaR* based on the joint annuity distribution is analogous to the main optimization in the main text (method section *Optimization of the stand-type composition in the planning units*).

Tables A1 to A6 show the resulting management regimes (rotation periods and species compositions) of the stand types. The main optimization (see main text) allocated area shares of the planning units to these stand types.

Table A1. Simulated stand types with the economically optimized tree-species composition (area shares) under the *none (0 %)* scenario. The economically optimized rotation periods vary between the planning units within the indicated ranges.

Stand type	Species	Optimal species composition	Optimal rotation period (min, median, max) [a]
bee	beech	100 %	90, 90, 150
dou	Douglas-fir	100 %	70, 80, 100
oak	oak	100 %	170, 180, 180
pin	pine	100 %	70, 80, 120
spr	spruce	100 %	70, 80, 80
dou_bee	Douglas-fir	60 %	80, 80, 110
	beech	40 %	90, 90, 150
spr_bee	spruce	90 %	70, 80, 90
	beech	10 %	90, 90, 150
dou_oak	Douglas-fir	90 %	70, 80, 100
	oak	10 %	170, 180, 180

Table A2. Simulated stand types with the economically optimized tree-species composition (area shares) under the *baseline (2 %)* scenario. The optimal rotation ages vary between the planning units within the indicated ranges.

Stand type	Species	Optimal species composition	Optimal rotation period (min, median, max) [a]
bee	beech	100 %	90, 90, 150
dou	Douglas-fir	100 %	70, 80, 100
oak	oak	100 %	170, 180, 180
pin	pine	100 %	70, 80, 120
spr	spruce	100 %	70, 80, 80
dou_bee	Douglas-fir	60 %	80, 80, 110
	beech	40 %	90, 90, 150
spr_bee	spruce	90 %	70, 80, 90
	beech	10 %	90, 90, 150
dou_oak	Douglas-fir	90 %	70, 80, 100
	oak	10 %	170, 180, 180

Table A3. Simulated stand types with the economically optimized tree-species composition (area shares) under the *intensified (4 %, more events)* scenario. The economically optimized rotation periods vary between the planning units within the indicated ranges.

Stand type	Species	Optimal species composition	Optimal rotation period (min, median, max) [a]
bee	beech	100 %	90, 90, 150
dou	Douglas-fir	100 %	70, 80, 100
oak	oak	100 %	170, 180, 180
pin	pine	100 %	70, 80, 120
spr	spruce	100 %	70, 80, 80
dou_bee	Douglas-fir	70 %	80, 80, 110
	beech	30 %	90, 90, 150
spr_bee	spruce	90 %	70, 80, 90
	beech	10 %	90, 90, 150
dou_oak	Douglas-fir	90 %	70, 80, 100
	oak	10 %	170, 180, 180

Table A4. Simulated stand types with the economically optimized tree-species composition (area shares) under the *intensified (4 %, larger events)* scenario. The economically optimized rotation periods vary between the planning units within the indicated ranges.

Stand type	Species	Optimal species composition	Optimal rotation period (min, median, max) [a]
bee	beech	100 %	90, 90, 150
dou	Douglas-fir	100 %	70, 80, 100
oak	oak	100 %	170, 180, 180
pin	pine	100 %	70, 80, 120
spr	spruce	100 %	70, 80, 80
dou_bee	Douglas-fir	70 %	80, 80, 110
	beech	30 %	90, 90, 150
spr_bee	spruce	90 %	70, 80, 90
	beech	10 %	90, 90, 150
dou_oak	Douglas-fir	90 %	70, 80, 100
	oak	10 %	170, 180, 180

Table A5. Simulated stand types with the economically optimized tree-species composition (area shares) under the *intensive (6 %, more events)* scenario. The economically optimized rotation periods vary between the planning units within the indicated ranges.

Stand type	Species	Optimal species composition	Optimal rotation period (min, median, max) [a]
bee	beech	100 %	90, 90, 150
dou	Douglas-fir	100 %	70, 80, 100
oak	oak	100 %	170, 180, 180
pin	pine	100 %	70, 80, 120
spr	spruce	100 %	70, 80, 80
dou_bee	Douglas-fir	70 %	80, 80, 110
	beech	30 %	90, 90, 150
spr_bee	spruce	90 %	70, 80, 90
	beech	10 %	90, 90, 150
dou_oak	Douglas-fir	90 %	70, 80, 100
	oak	10 %	170, 180, 180

Table A6. Simulated stand types with the economically optimized tree-species composition (area shares) under the *intensive (6 %, larger events)* scenario. The economically optimized rotation periods vary between the planning units within the indicated ranges.

Stand type	Species	Optimal species composition	Optimal rotation period (min, median, max) [a]
bee	beech	100 %	90, 90, 150
dou	Douglas-fir	100 %	70, 80, 100
oak	oak	100 %	170, 180, 180
pin	pine	100 %	70, 80, 120
spr	spruce	100 %	70, 80, 80
dou_bee	Douglas-fir	70 %	80, 80, 110
	beech	30 %	90, 90, 150
spr_bee	spruce	90 %	70, 80, 90
	beech	10 %	90, 90, 150
dou_oak	Douglas-fir	90 %	70, 80, 100
	oak	10 %	170, 180, 180

A1.2 Climate

Table A7. Climate conditions in the forest enterprise derived based on the WorldClim 1.4 data set[4] for the period 2060-2080 under the RCP 8.5 scenario [MPI-ESM-LR]. Coefficient of variation (CV), Minimum and Maximum refer to the variation between the planning units.

Variable	Mean	CV	Minimum	Maximum
annual mean temperature [°C]	11.1	0.05	9.6	11.8
maximum temperature warmest month [°C]	25.3	0.03	23.6	26.3
minimum temperature coldest month [°C]	0.6	1.24	-0.9	1.6
annual precipitation [mm]	764	0.14	603	1012
precipitation warmest quarter [mm]	193	0.10	160	235

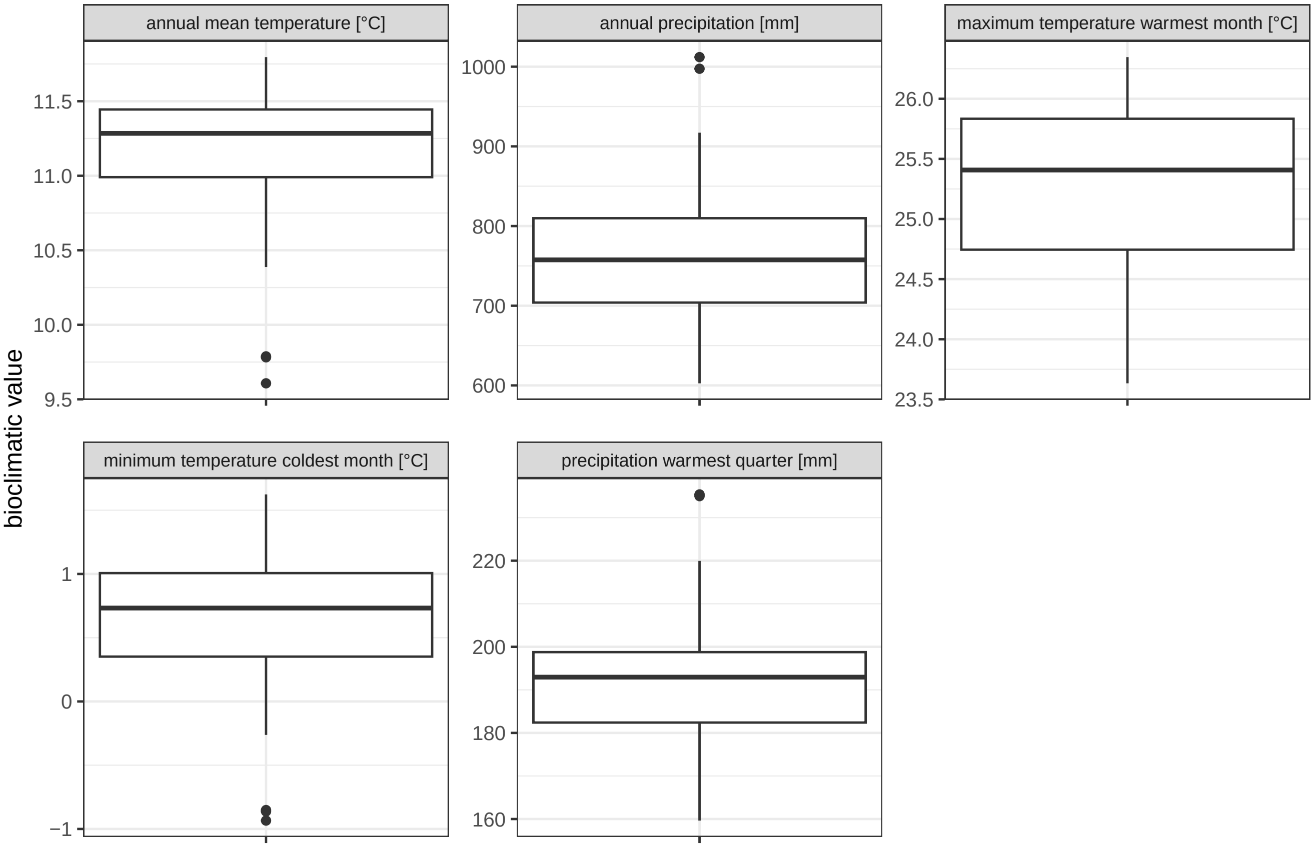

Figure A1. Variability of climate conditions across the 24 planning units derived based on the WorldClim 1.4 data set[4] for the period 2060-2080 under the RCP 8.5 scenario [MPI-ESM-LR]. The climate conditions implemented spatial heterogeneity in terms of growth and survival probabilities in our model.

A1.3 Forest growth

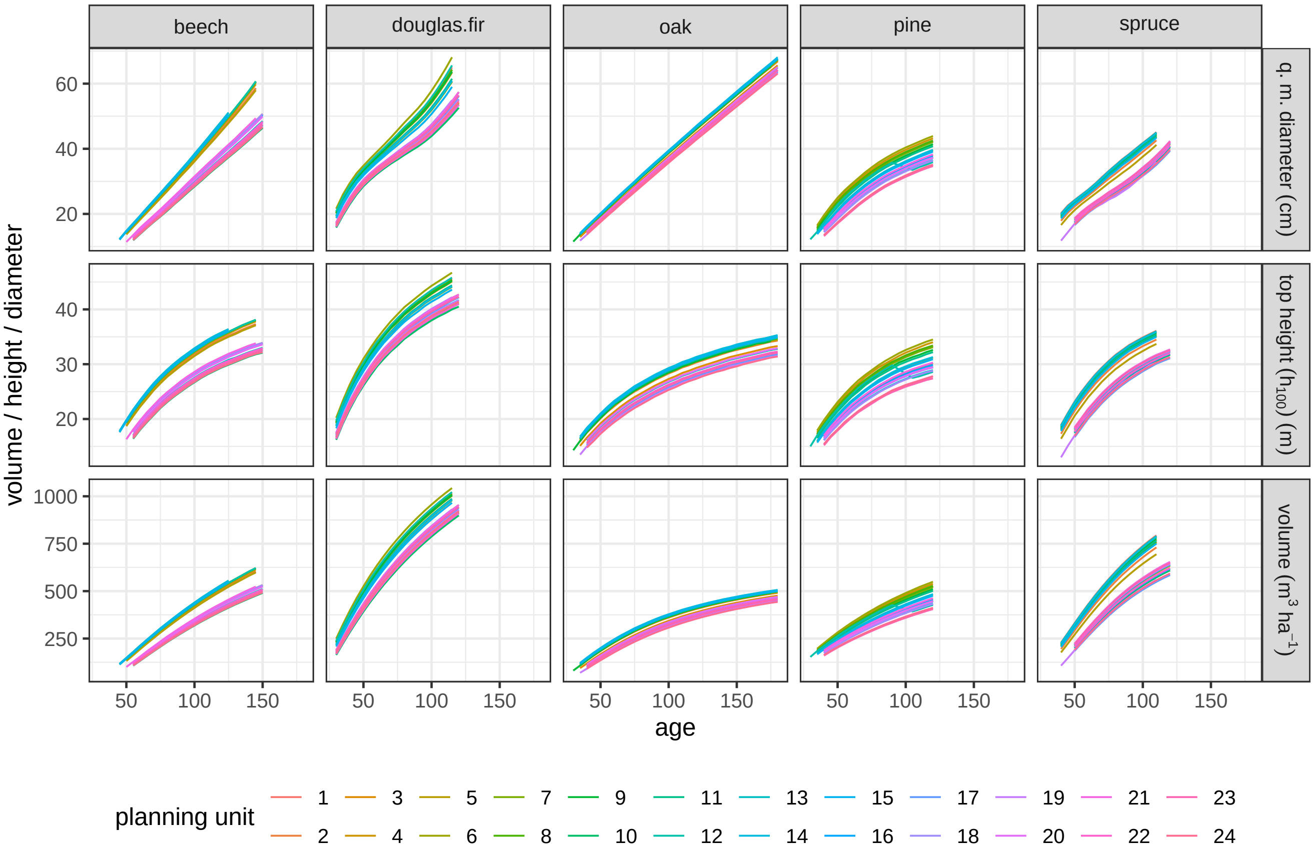

Figure A2. Growth characteristics derived for the 5 species in the 24 planning units illustrating the heterogeneity of forest growth. Three variables are shown, the quadratic mean diameter, the top height of the 100 thickest trees and the standing volume.

A1.4 Disturbance simulation

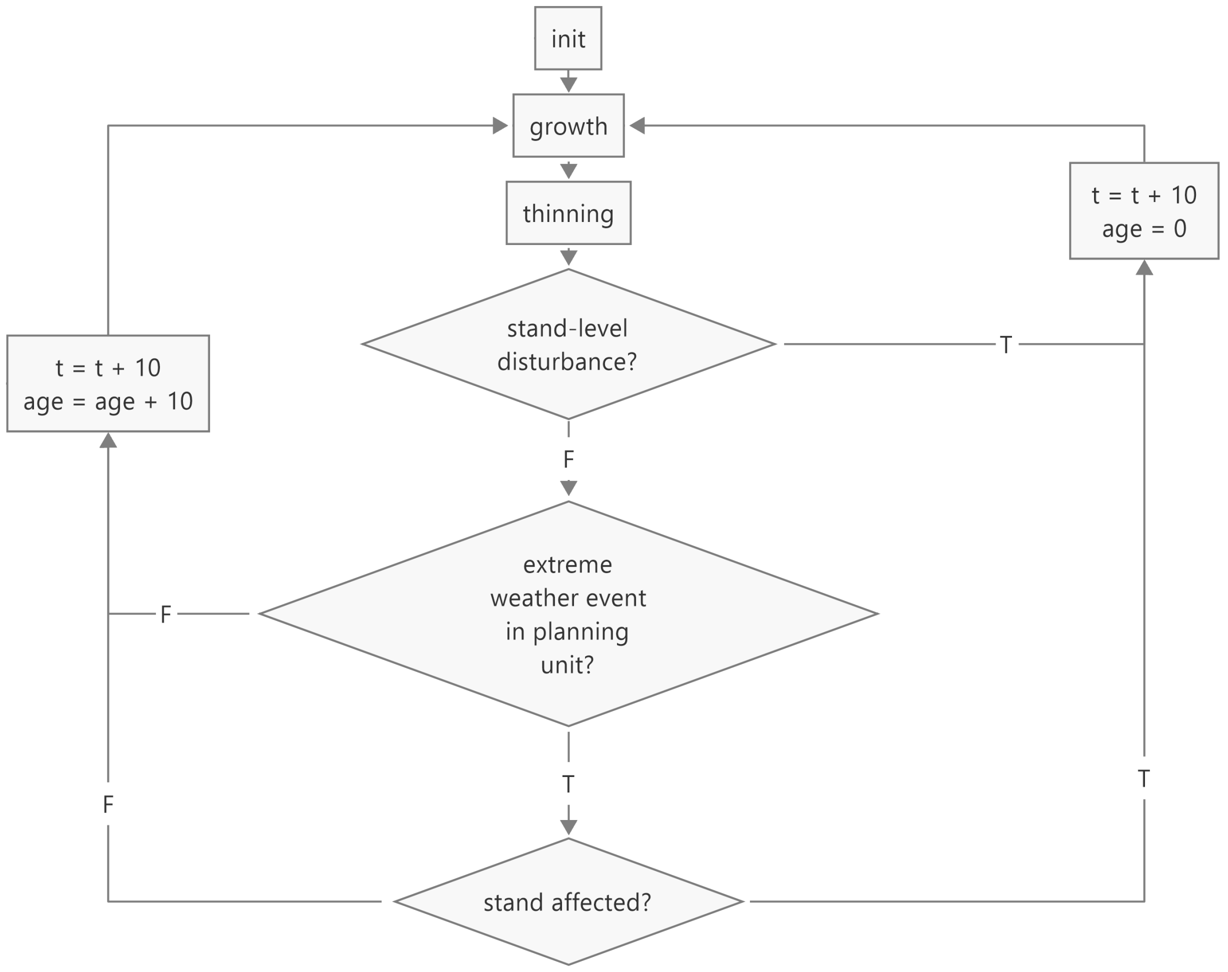

Figure A3. Flowchart of the growth and disturbance simulation with stand-level disturbances and extreme weather events.

A1.4.1 Stand-level disturbances

For the probability of stand-level disturbances, we apply the climate-sensitive survival functions developed by Brandl *et al.* [1] based on the pan-European ICP forests data set (Level I and II). The unconditional probability S that a stand reaches a specific age t is described with a Weibull distribution,

$$S(t) = \exp\left[-\left(\frac{t}{\beta}\right)^{\alpha}\right], \qquad (A5)$$

with a shape parameter α and a scale parameter β. In addition to bioclimatic variables, the parameters for some species are also sensitive to the share of the species itself in the stand, implementing the stabilizing effect of species mixture in our model. Fig. A4 shows the Weibull survival functions for the climate conditions in the planning units of the model forest enterprise.

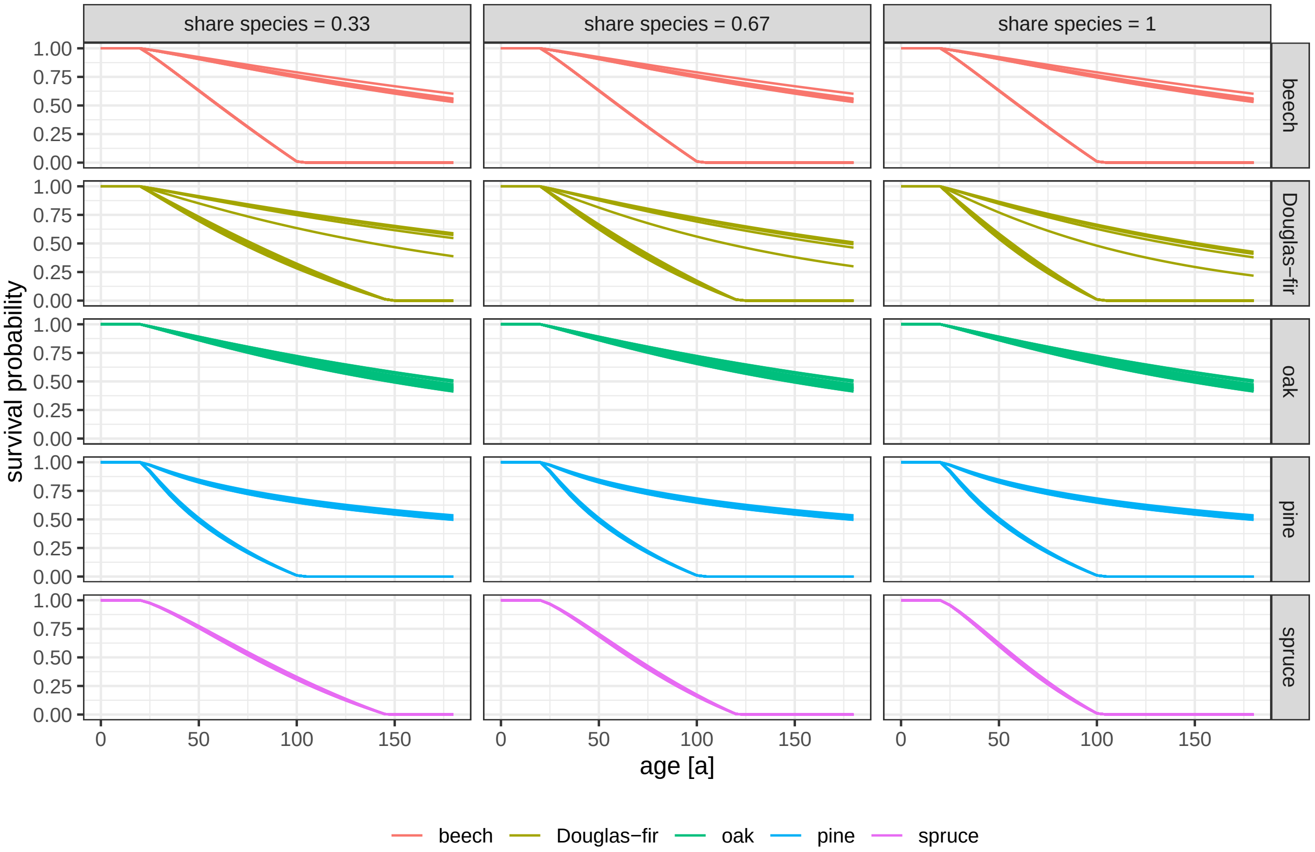

Figure A4. Survival probabilities of the species (panel rows) over age based on Brandl *et al.* [1]. The different shares of the respective species in the stand (panel columns) illustrate the stabilizing effect of mixture in the case of Douglas-fir and spruce [1]. The survival probabilities shown here are based only on the stand-level disturbances and do not include the additional disturbance probabilities due to extreme weather events implemented in our Monte-Carlo simulation. The multiple lines in each panel represent the 24 planning units. Large differences between these lines (e.g., in the case of beech) indicate that in some of the planning units, the projected climate variables were out of the range of these variables in the original European data set used to fit the models [1]. In this case, we decreased the probability to reach age 100 to 1 % in monocultures. For spruce, this was the case in all planning units.

Based on the unconditional survival probabilities, we derived hazard rates as conditional probability $h(t)$ that a stand that has already reached a given age $(t-10)$ is disturbed within a 10-year simulation period [3;5],

$$h(t) = \frac{S(t-10) - S(t)}{S(t-10)}. \qquad (A6)$$

These hazard rates (Fig. A5) can be directly applied in the stochastic Monte-Carlo simulation as probabilities of stand-level disturbances in a simulation period [6].

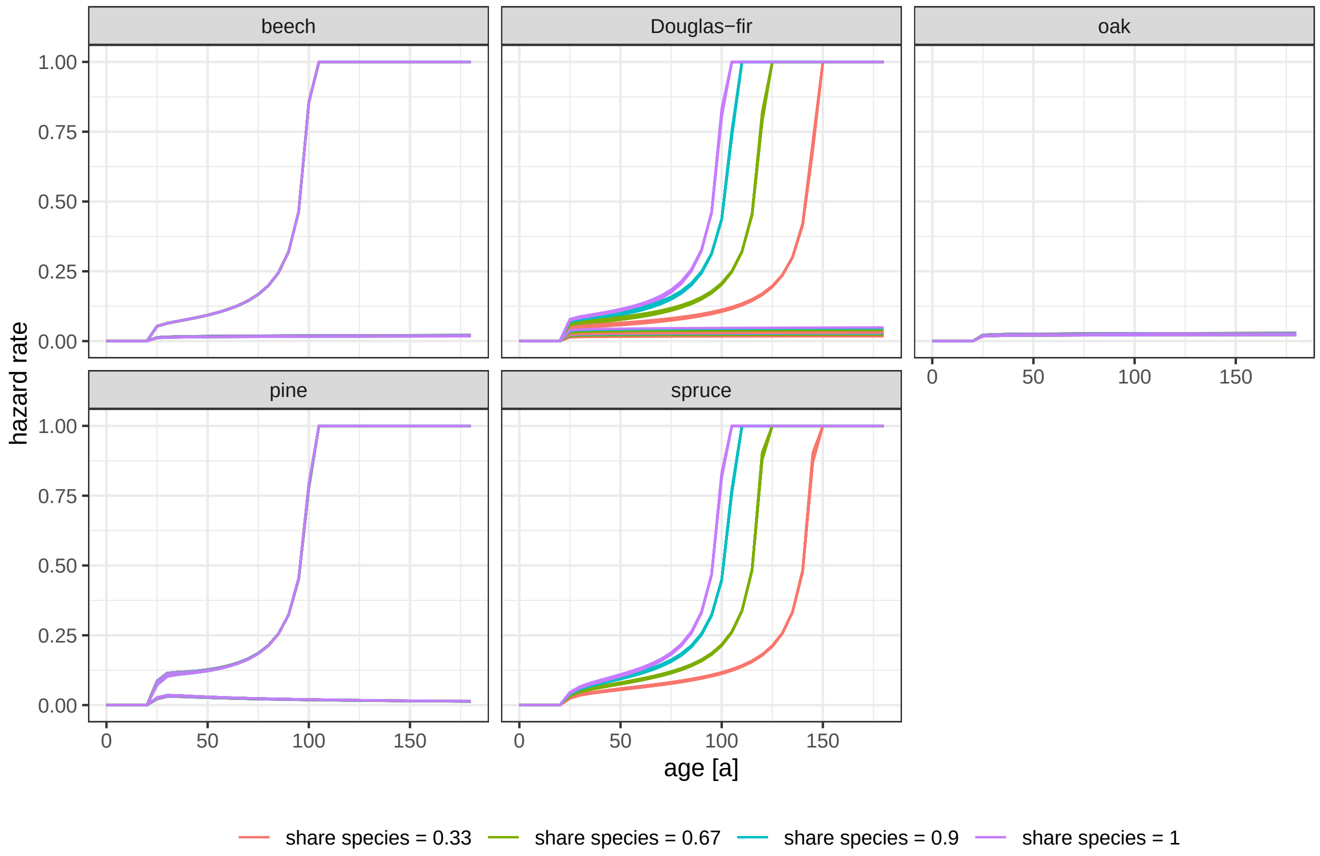

Figure A5. Hazard rates of the species (panels) over age based on Brandl *et al.*[1]. The different shares of the respective species in the stand (colors) illustrate the stabilizing effect of mixture in the case of Douglas-fir and spruce[1]. The hazard rates shown here are based only on the stand-level disturbances and do not include the additional disturbance probabilities due to extreme weather events implemented in our Monte-Carlo simulation. The multiple lines in each panel represent the 24 planning units. Large differences between these lines (e.g., in the case of beech) indicate that in some of the planning units, the projected climate variables were out of the range of these variables in the original European data set used to fit the models[1]. In this case, we decreased the probability to reach age 100 to 1 % in monocultures. For spruce, this was the case in all planning units.

With the survival time models by Brandl *et al.*[1], we applied an up-to-date survival time analysis that accounts for bioclimatic variables as well as a stabilizing effect of species mixture. The statistical models were fitted based on a pan-European data set, however, the data set covered only the time period from 2010-2017. For instance, risks due to drought periods as observed 2018-2020 in Germany, may thus be underestimated. The model further assumes linear effects of bioclimatic conditions and species mixture. In our simulations, admixing 10 % of a broadleaved species to spruce or Douglas-fir thus already results in slightly higher survival probabilities. The survival time models do not account for the identity of the admixed species. We decided to apply a conservative approach; only admixing a broadleaved species to conifers had a stabilizing effect. We did not account for possible stabilizing effects of conifer mixtures. For a more detailed discussion of the survival time models and the underlying assumptions, we refer the reader to the original publications[1;6].

A1.4.2 Poisson point process for extreme weather events

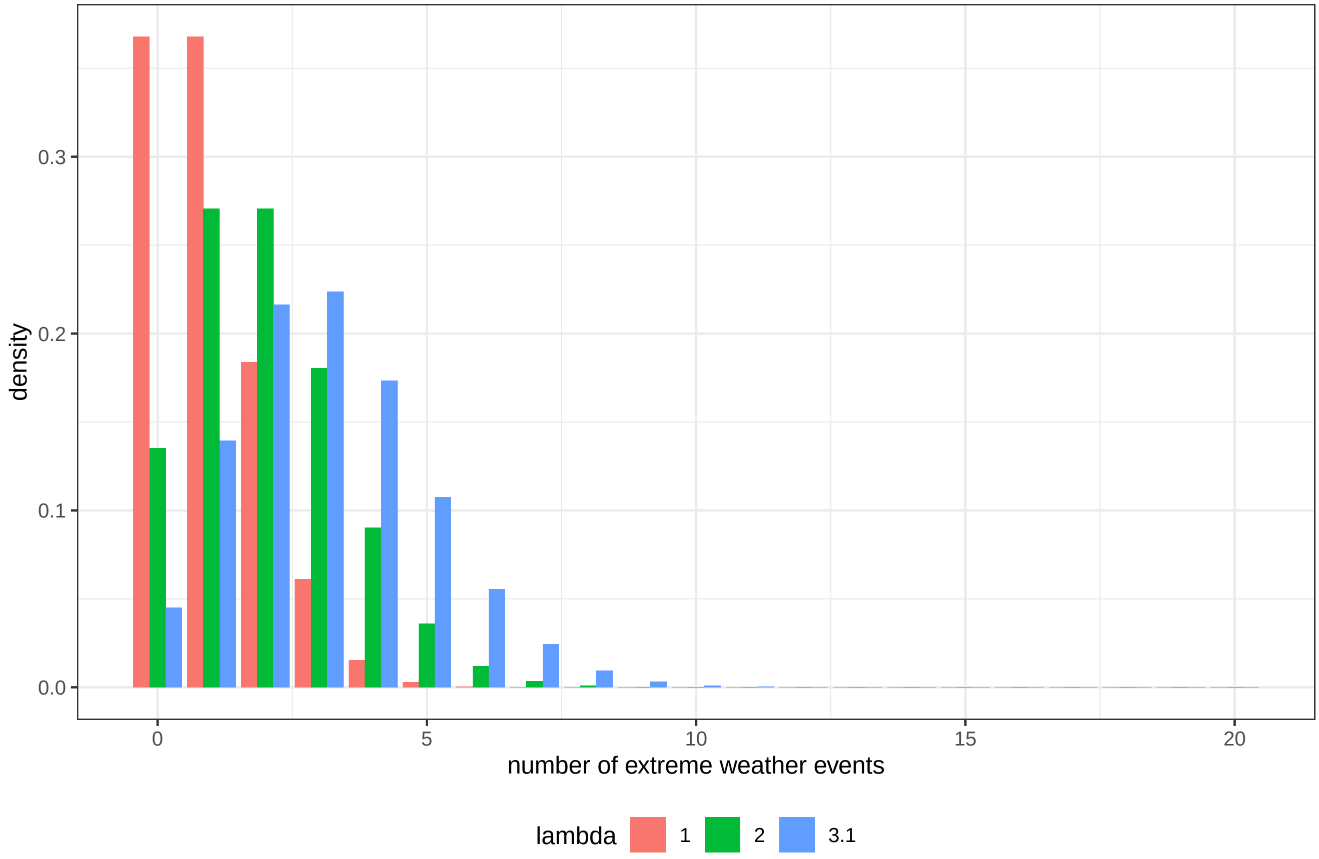

Figure A6. Density of the number of extreme weather events per 10-year period in the study region for different assumptions on the expected number of events λ.

A1.4.3 Extreme-event hazard rates

In the case of an extreme weather event, we assumed a considerable increase in hazard rates. These hazard rates should represent the stability ranking of different tree species as well as that mixed stands of some species are assumed to be more stable than monocultures[1]. We translated the hazard rates derived for stand-level disturbances $h_{standlevel}$ to extreme-event hazard rates $h_{extreme}$ using a logistic model allowing us to define a minimum h_{min} and maximum hazard rate h_{max}:

$$h_{extreme} = \frac{h_{max} - h_{min}}{1 + e^{-8 \cdot h_{standlevel} + 2}} + h_{min}. \tag{A7}$$

The resulting relationship between stand-level and extreme-event hazard rates for $h_{min} = 0.7$ and $h_{max} = 0.99$ is illustrated in Fig. A7. The resulting hazard rates in case of extreme weather events are illustrated in Fig. A8.

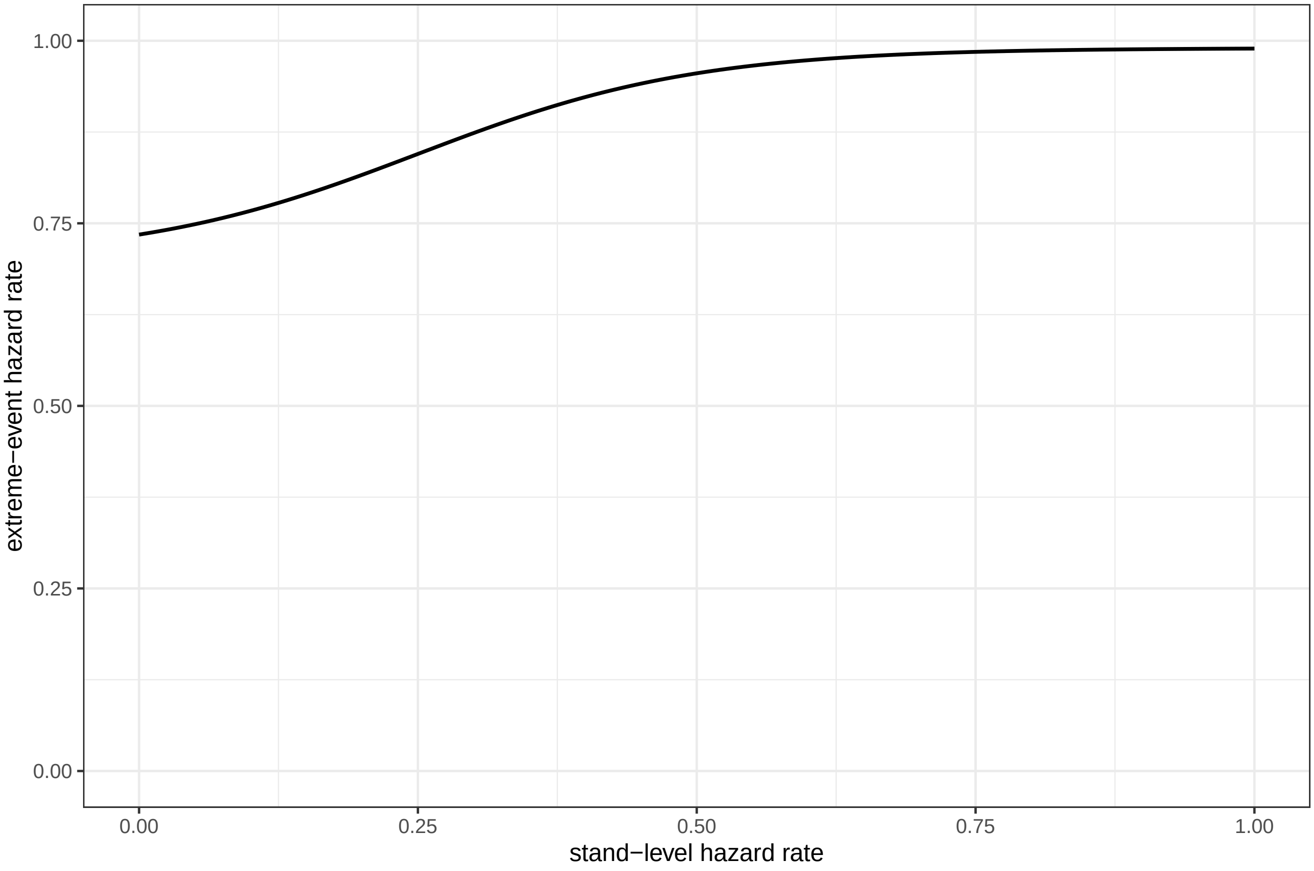

Figure A7. Logistic function translating the hazard rates for stand-level disturbances according to Brandl *et al.*[1] (calculated from the survival functions in Fig. A4) into hazard rates in the case of an extreme weather event. This implements a high probability of disturbance for all stands in the influence zone of an extreme weather event, while accounting for differences in the resistance dependent on species identity and species mixture. However, these differences are smaller than in the original survival functions by Brandl *et al.*[1].

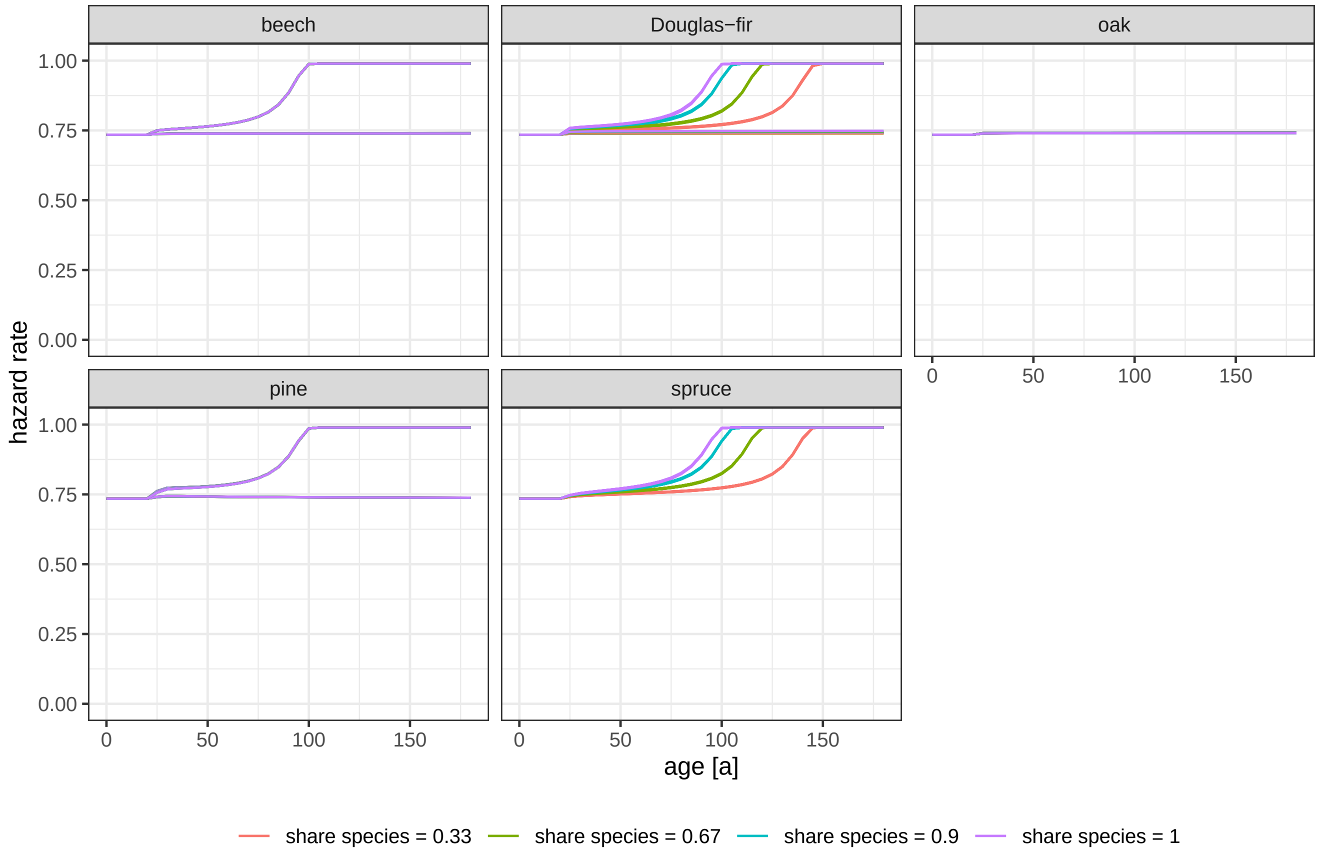

Figure A8. Hazard rates of the species (panels) over age given that there is an extreme weather event at the stand's location. The different shares of the respective species in the stand (colors) illustrate the stabilizing effect of mixture in the case of Douglas-fir and spruce[1]. The hazard rates shown here are based only on the hazard rates for stand-level disturbances based on Brand *et al.*[1]. However, we rescaled them to values between 0.7 and 0.99 to implement high disturbance probabilities given an extreme weather event (see Eq. (A7)). The multiple lines in each panel represent the 24 planning units. Large differences between these lines (e.g., in the case of beech) indicate that in some of the planning units, the projected climate variables were out of the range of these variables in the original European data set used to fit the models[1]. In this case, we decreased the probability to reach age 100 (based on stand-level disturbances only) to 1 % in monocultures. For spruce, this was the case in all planning units.

A1.5 Economic valuation

A1.5.1 Wood revenues and harvest costs

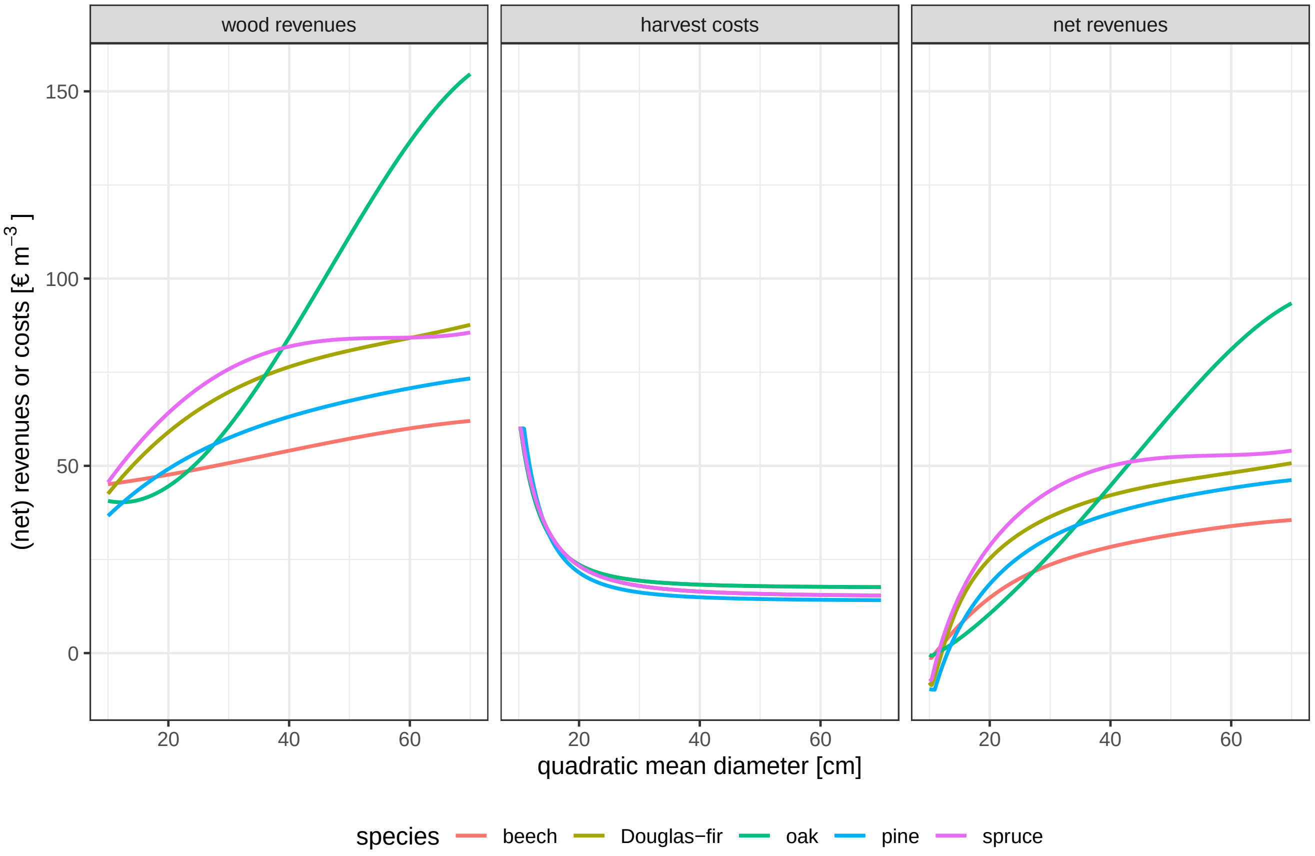

Figure A9. Wood revenues, harvest costs, and net revenues dependent on the tree species and quadratic mean diameter of the harvested trees. The values are derived using the `woodValuationDE` model[7]. The revenues refer to one cubic meter salable wood, the harvesting costs to one cubic meter skidded wood, and the net revenues to one cubic meter standing volume over bark.

A1.5.2 Planting costs

Table A8. Planting costs for the simulated species $[€\,ha^{-1}]$ dependent on the age of the previous stand, based on Möhring *et al.*[8]. Since establishing Douglas-fir is often not successful and replanting is required, we assumed a replanting of 50 %.

Age	Spruce	Douglas-fir	Pine	Oak	Beech
0	1599	5550	2524	8994	2710
10	3600	5550	5800	16500	8800
20	3600	5550	5800	16500	8800
30	3600	5550	5800	16500	8800
40	3600	5550	5800	16500	8800
50	3267	5391	5254	15249	7785
60	2933	5232	4708	13998	6770
70	2600	5073	4162	12747	5755
80	2266	4914	3616	11496	4740
90	1933	4755	3070	10245	3725
100	1599	4596	2524	8994	2710
110	1599	4596	2524	8994	2710
120	1599	4596	2524	8994	2710
130	1599	4596	2524	8994	2710
140	1599	4596	2524	8994	2710
150	1599	4596	2524	8994	2710
160	1599	4596	2524	8994	2710
170	1599	4596	2524	8994	2710
180	1599	4596	2524	8994	2710

A1.5.3 External market fluctuations

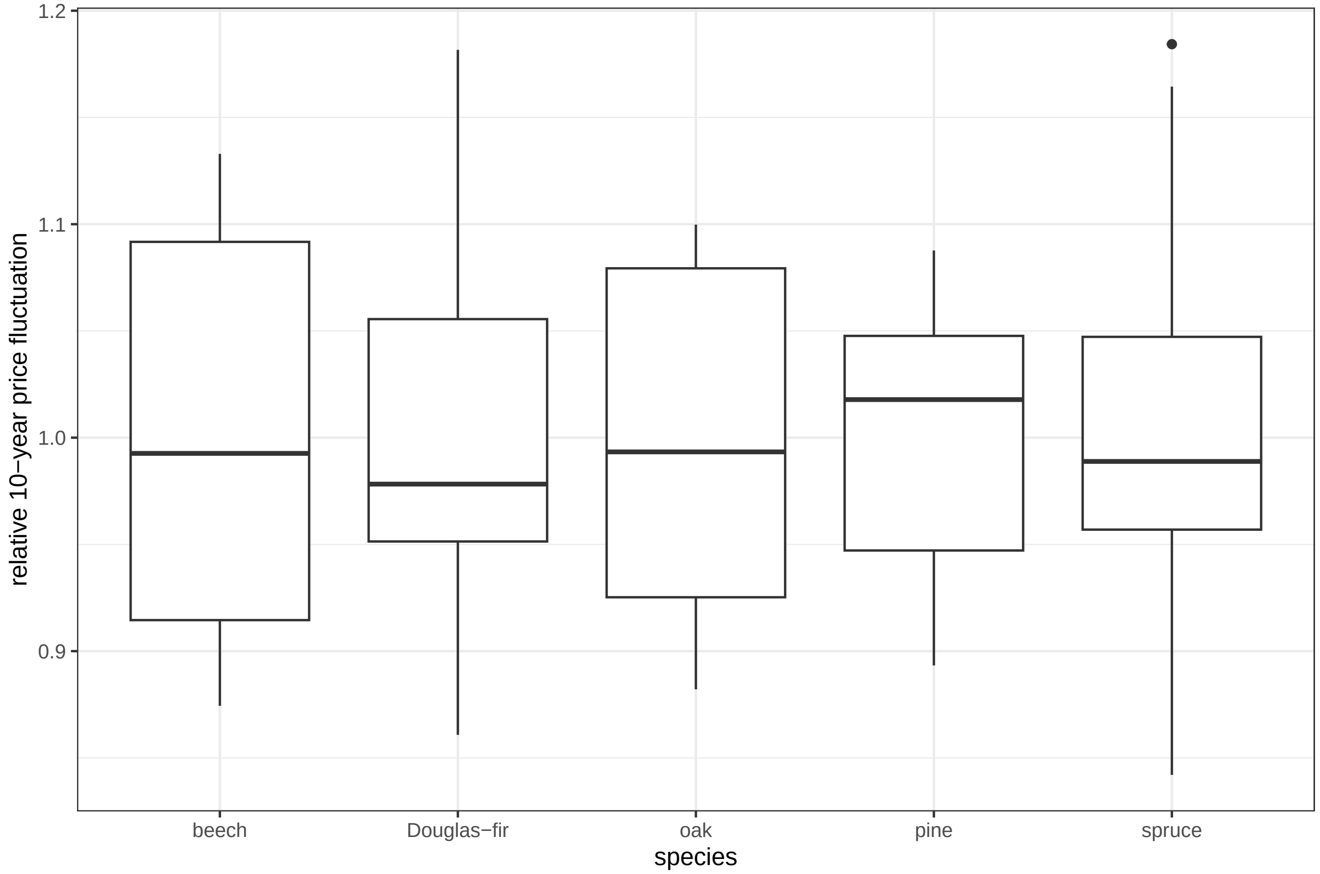

Figure A10. Boxplots of market prices of the 5 species used in the bootstrapping process implementing external fluctuations of 10-year moving averages of market prices[9]. The underlying data set is based on historic prices of the Bavarian state forests, Germany.

Table A9. Correlations of market prices of the 5 species used in the bootstrapping process implementing external fluctuations of market prices[9]. The underlying data set adapted to 10-year periods are historic prices of the Bavarian state forests, Germany.

	Spruce	Beech	Douglas-fir	Oak	Pine
Spruce	1.00	-0.62	0.95	0.67	0.13
Beech	-0.62	1.00	-0.71	-0.99	-0.48
Douglas-fir	0.95	-0.71	1.00	0.74	0.39
Oak	0.67	-0.99	0.74	1.00	0.43
Pine	0.13	-0.48	0.39	0.43	1.00

A2 Supplementary results

A2.1 Diversity under larger extreme weather events

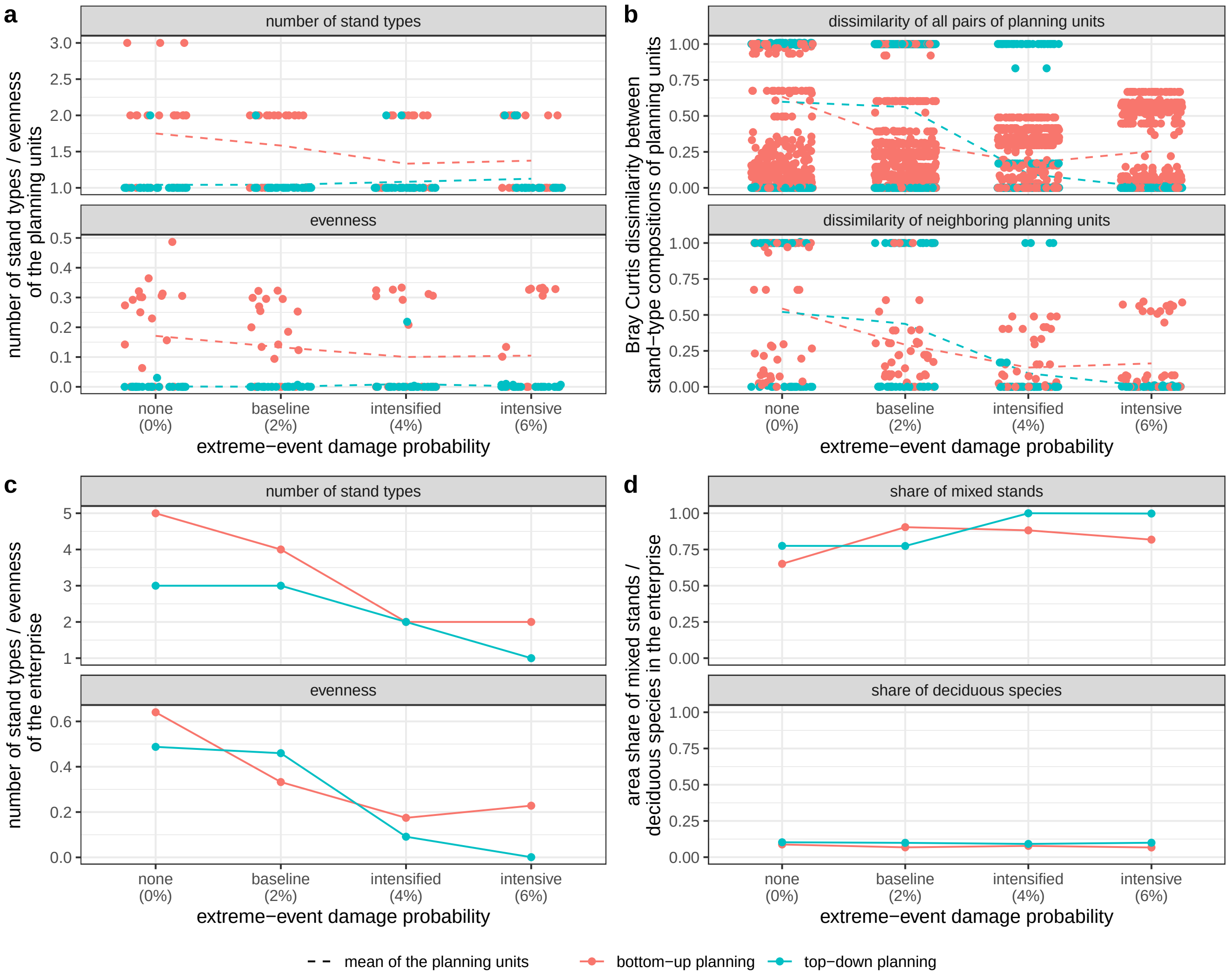

Figure A11. α- (a) β- (b) γ- (c), structural and functional diversity (d) of the optimal stand-type allocation comparing bottom-up and top-down planning under extreme-event scenarios defined by a damage probability (horizontal axis). Here, we show the results for increasing damage probabilities due to larger instead of more events as presented in the main text. Each point in (a) represents one of the 24 planning units, in (b) one pair of planning units. (c) and (d) refer to the entire enterprise.

A2.2 Optimal stand-type composition for risk-neutral decision makers

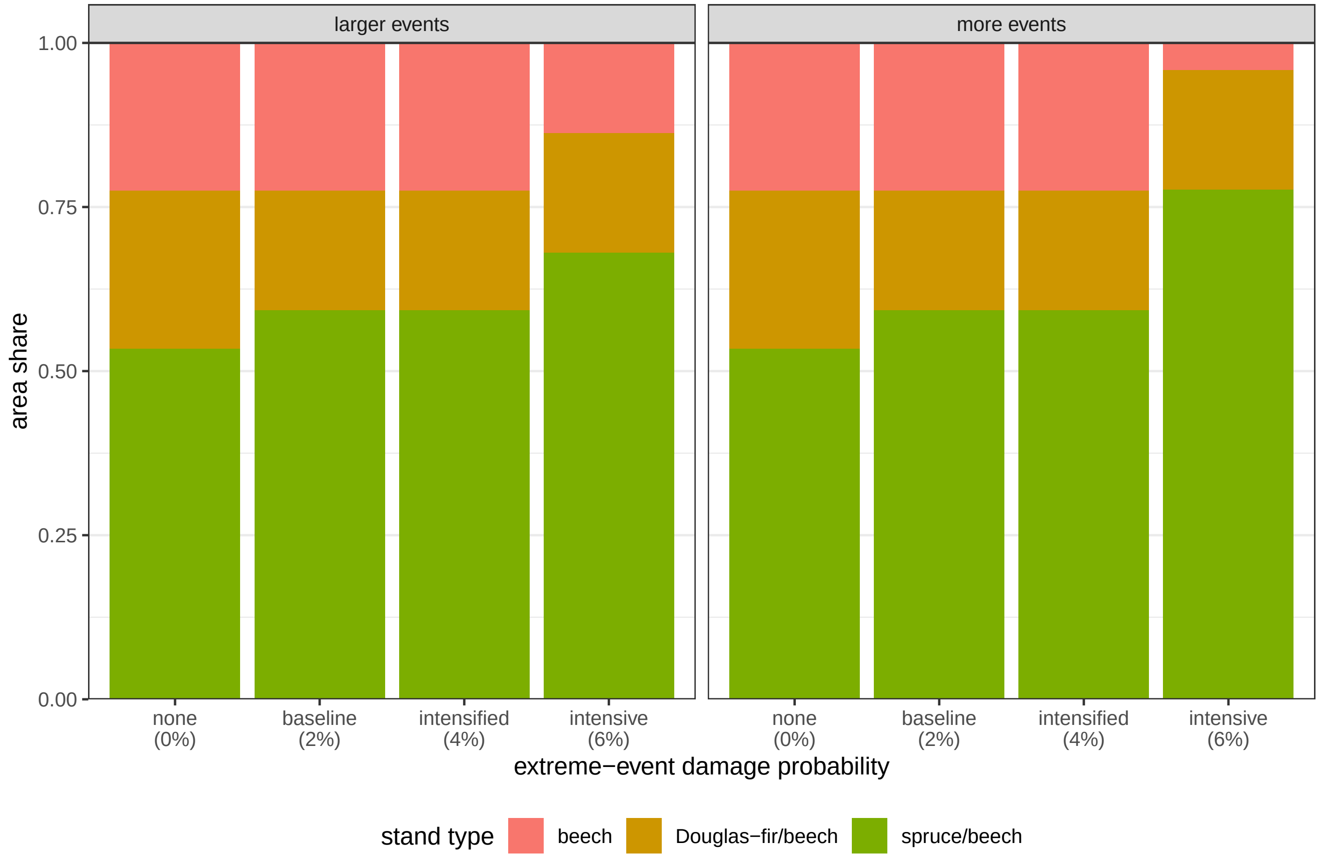

Figure A12. Optimized stand-type composition of the forest enterprise reflecting γ-diversity assuming a risk-neutral decision maker who seeks to maximize the expected return rather than *CVaR*. Optimal compositions for top-down and bottom-up planning are identical under risk-neutral decision making. We show the results for different extreme-event scenarios, defined by a probability of stand damage within 10 years (horizontal axis) and an increase in the size or number of events (panel columns).

A2.3 Spatial heterogeneity and stand-type allocation

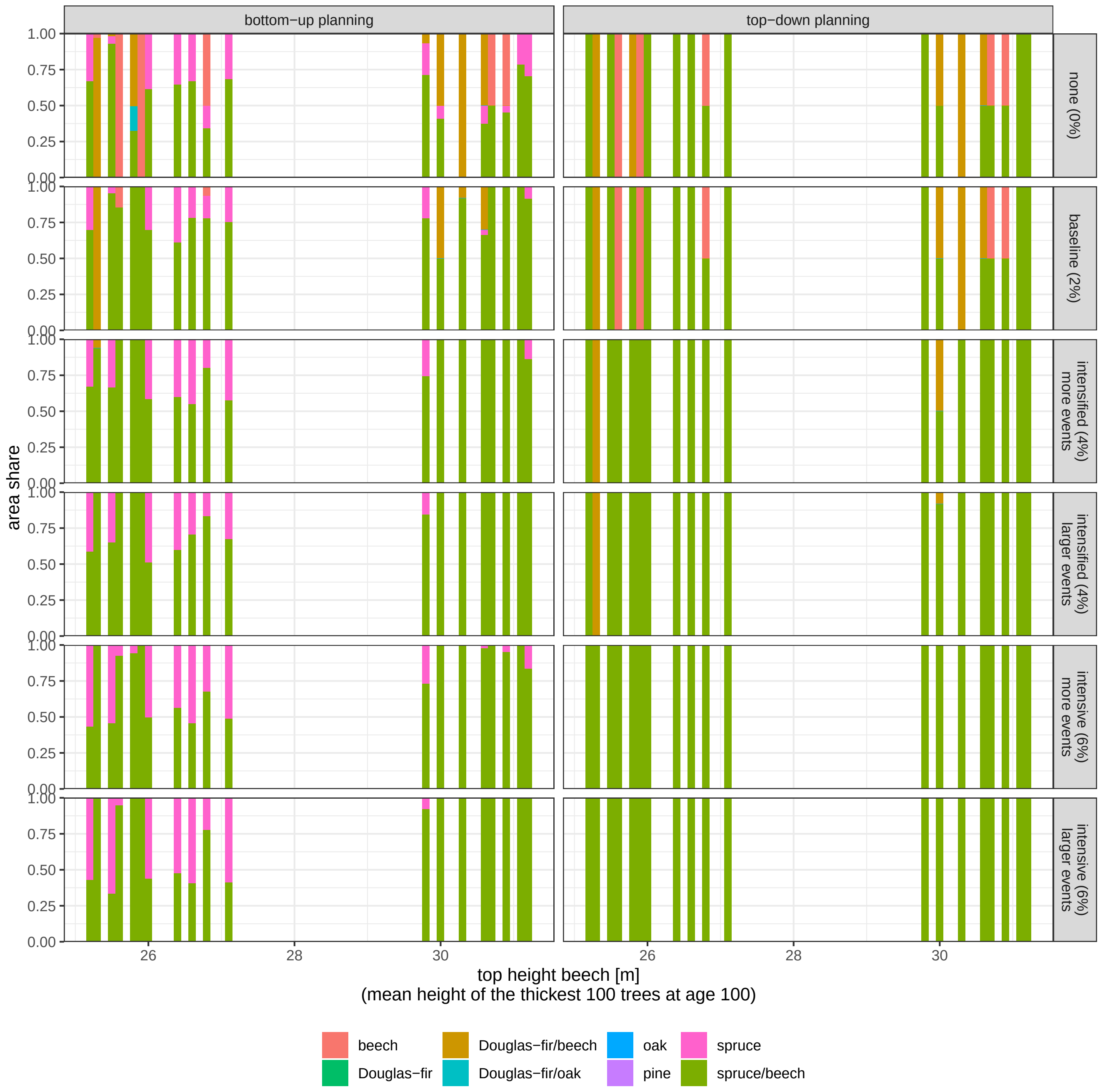

Figure A13. Optimal stand-type composition of the planning units over the planning unit's site productivity, expressed as top height of beech (horizontal axis). The stand-type compositions are shown for different planning perspectives (panel columns) and extreme-event scenarios (panel rows).

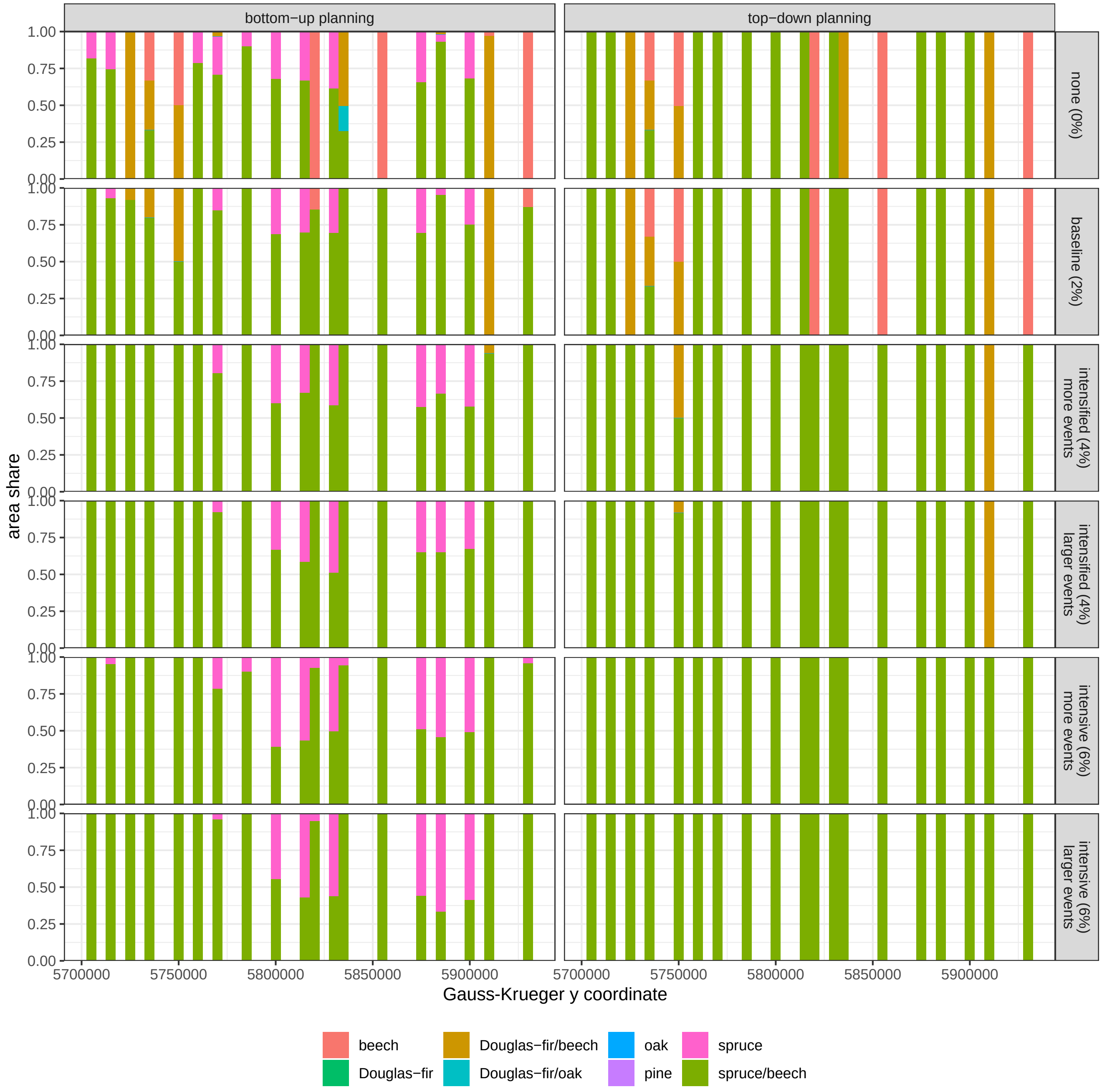

Figure A14. Optimal stand-type composition of the planning units over the planning unit's latitude (center coordinate, horizontal axis). The stand-type compositions are shown for different planning perspectives (panel columns) and extreme-event scenarios (panel rows).

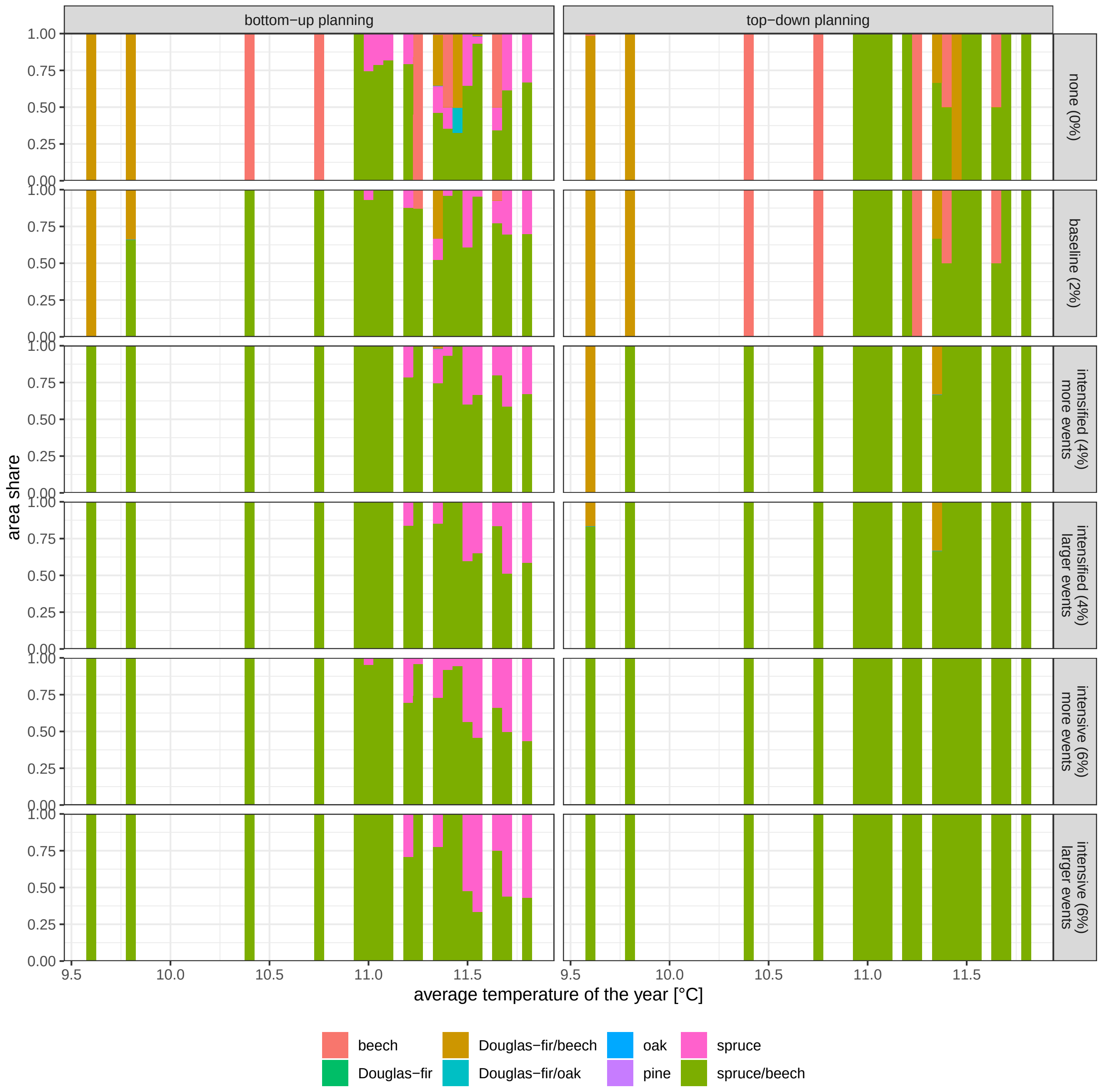

Figure A15. Optimal stand-type composition of the planning units over the planning unit's average temperature of the year (horizontal axis). The stand-type compositions are shown for different planning perspectives (panel columns) and extreme-event scenarios (panel rows).

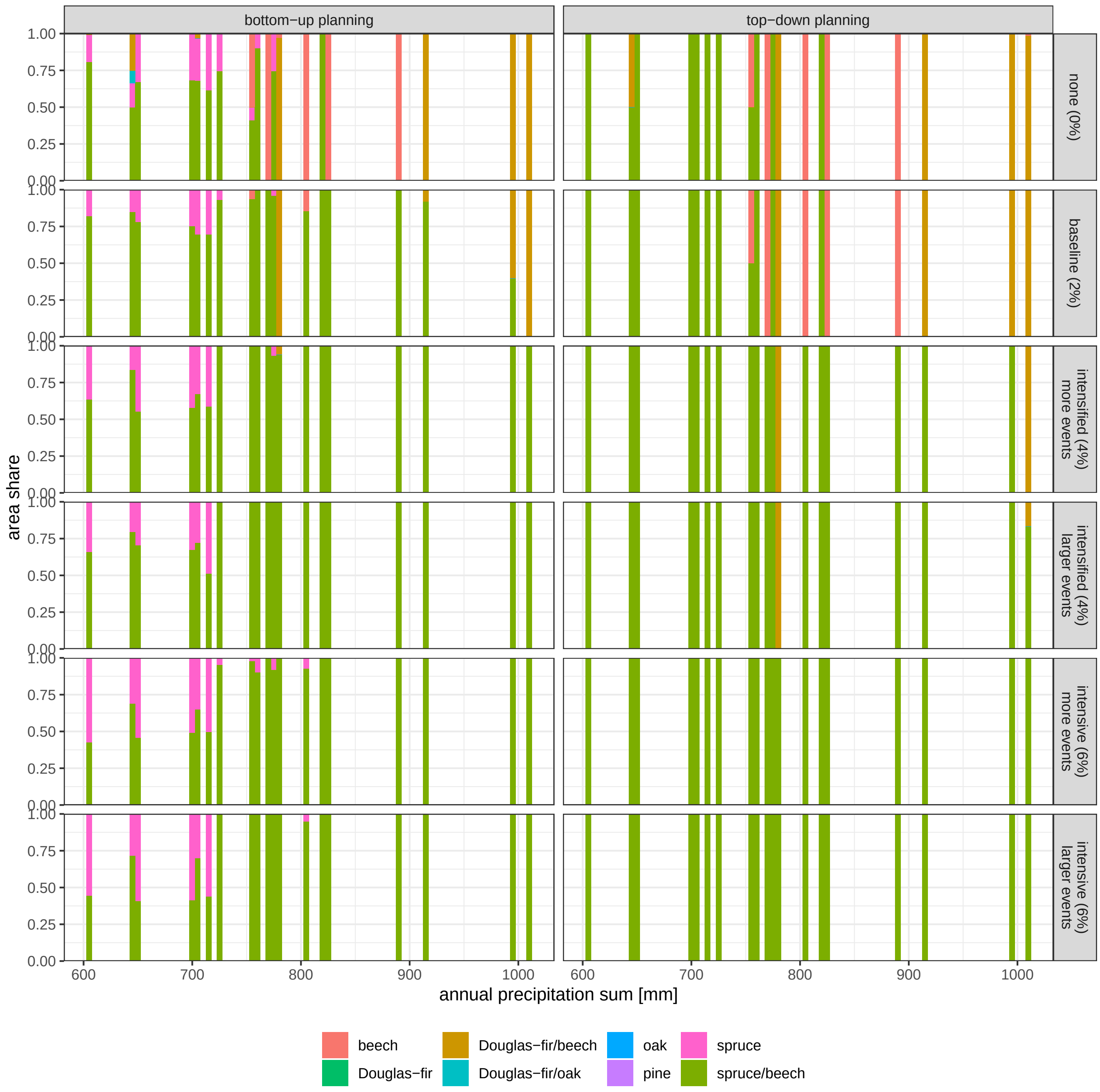

Figure A16. Optimal stand-type composition of the planning units over the planning unit's annual precipitation sum (horizontal axis). The stand-type compositions are shown for different planning perspectives (panel columns) and extreme-event scenarios (panel rows).

A2.4 Economic performance under larger events

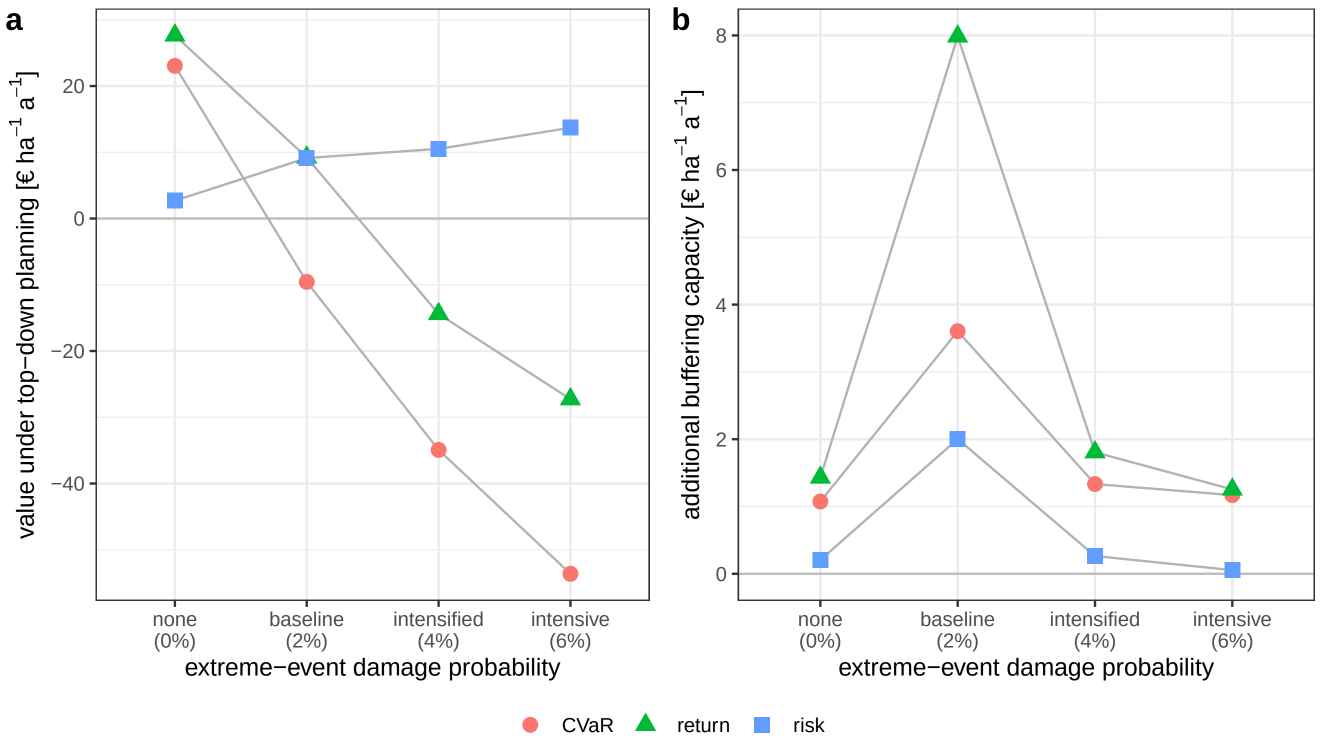

Figure A17. Economic performance of the forest enterprise under the optimized stand-type allocations from the top-down perspective (a) and additional buffering capacity of top-down planning, i.e., top-down value minus bottom-up value (b). *CVaR*, the Conditional Value at Risk (10 %-quantile) is the maximized objective function, return refers to the expected annuity (annual economic return) and risk to the standard deviation of the annuity in a Monte-Carlo simulation with 10 000 repetitions. The performance is shown for different extreme-event scenarios defined by a damage probability (horizontal axis) referring to an increase in the size of events rather than an increase in the number of events as presented in the main text.

A2.5 Economic performance of the planning units

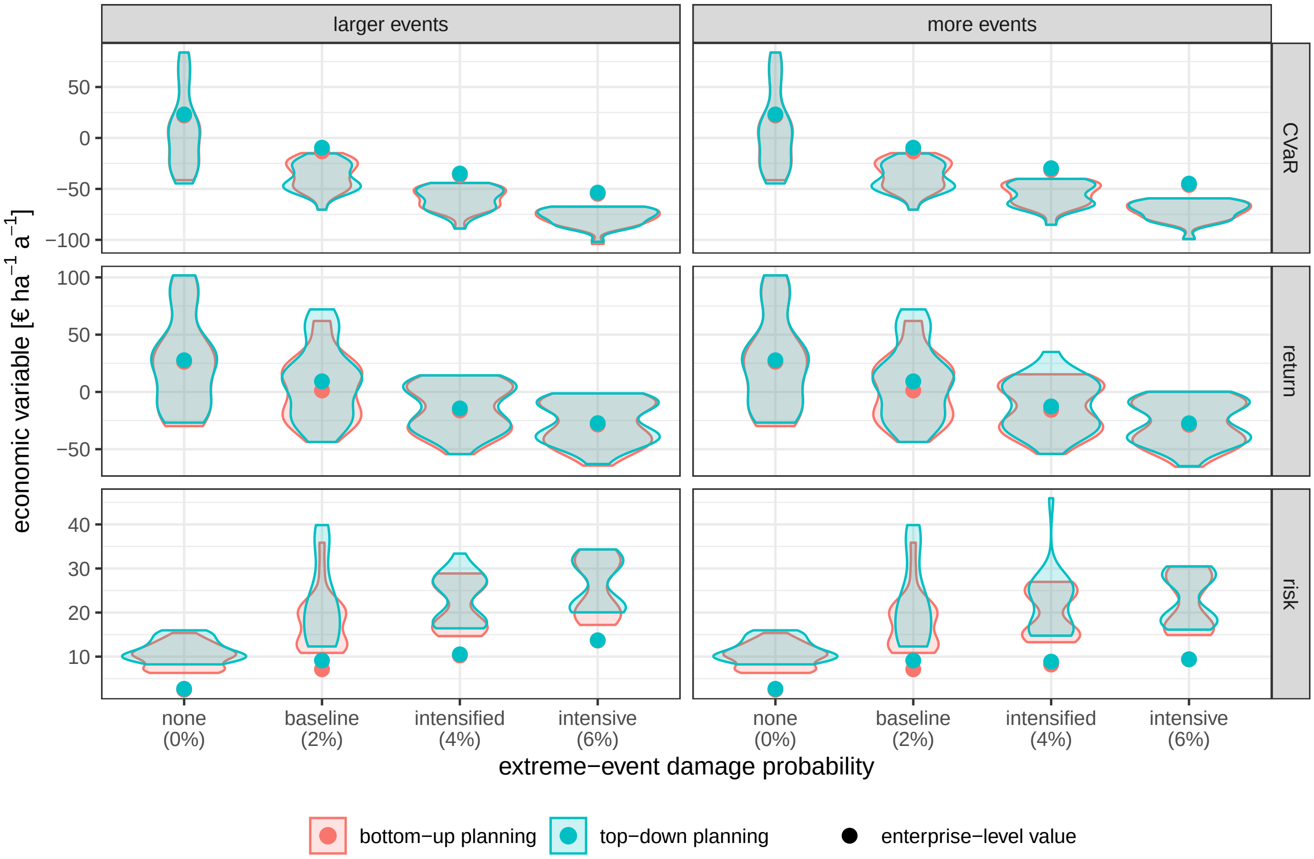

Figure A18. Densities of the economic performance of the single planning units dependent on the planning perspective and extreme-event scenario. CVaR: Conditional Value at Risk (10 %-quantile), return as expected value of the annuity distribution and risk as standard deviation of the annuity distribution. The violin diagrams show the density based on the 24 planning units. The points give the respective values for the entire enterprise.

A2.6 Joint annuity distribution at the enterprise level

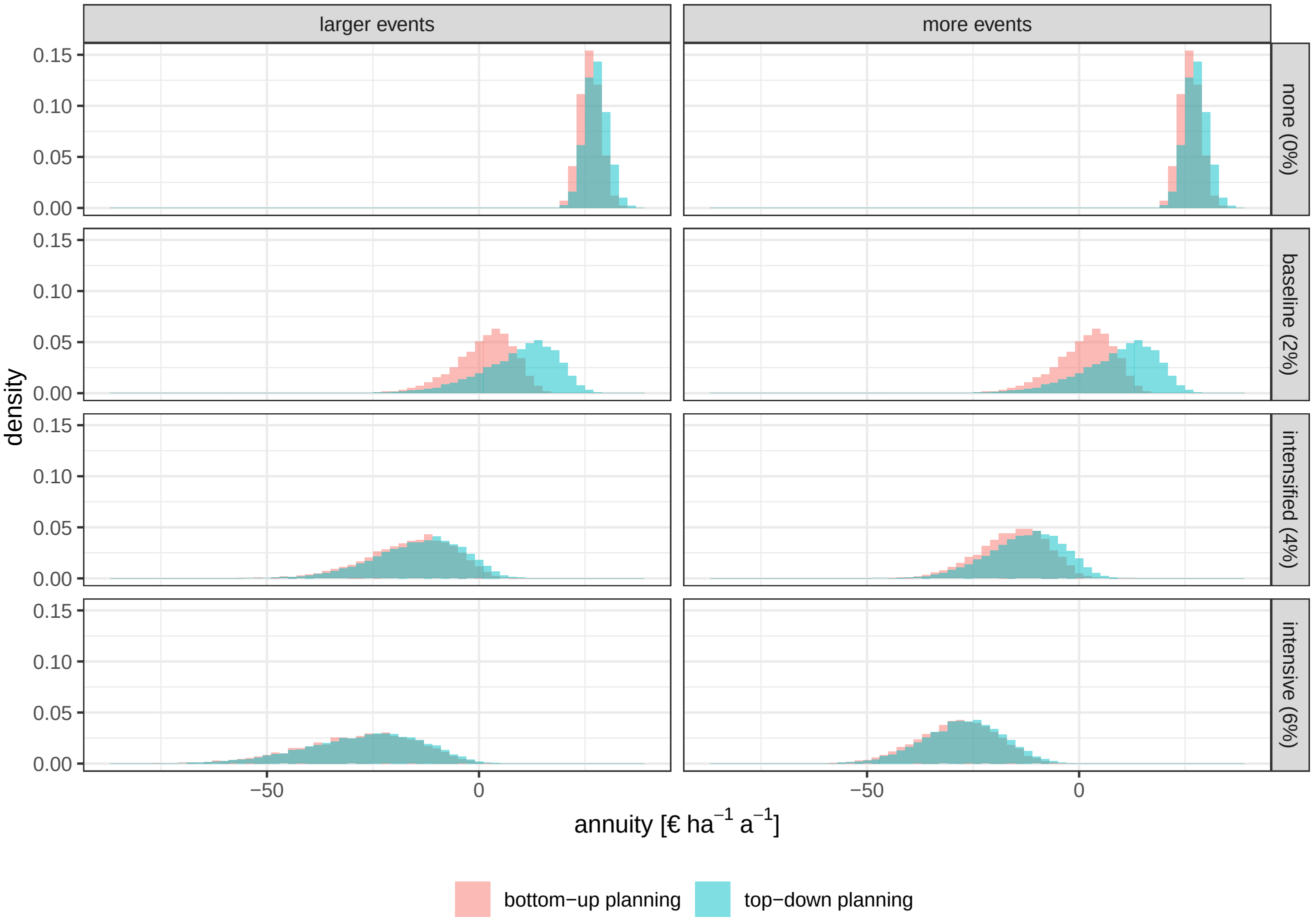

Figure A19. Densities of annual economic return (annuity) based on the 10 000 repetitions of the Monte-Carlo simulation. The densities refer to the joint distribution of the optimal stand-type allocation at the enterprise level. They depend on the planning perspective (colors) and scenarios of future extreme-event patterns with an increasing damage probability (panel rows) due to larger or more events (panel columns).

A2.7 Annuity distributions of the stand types

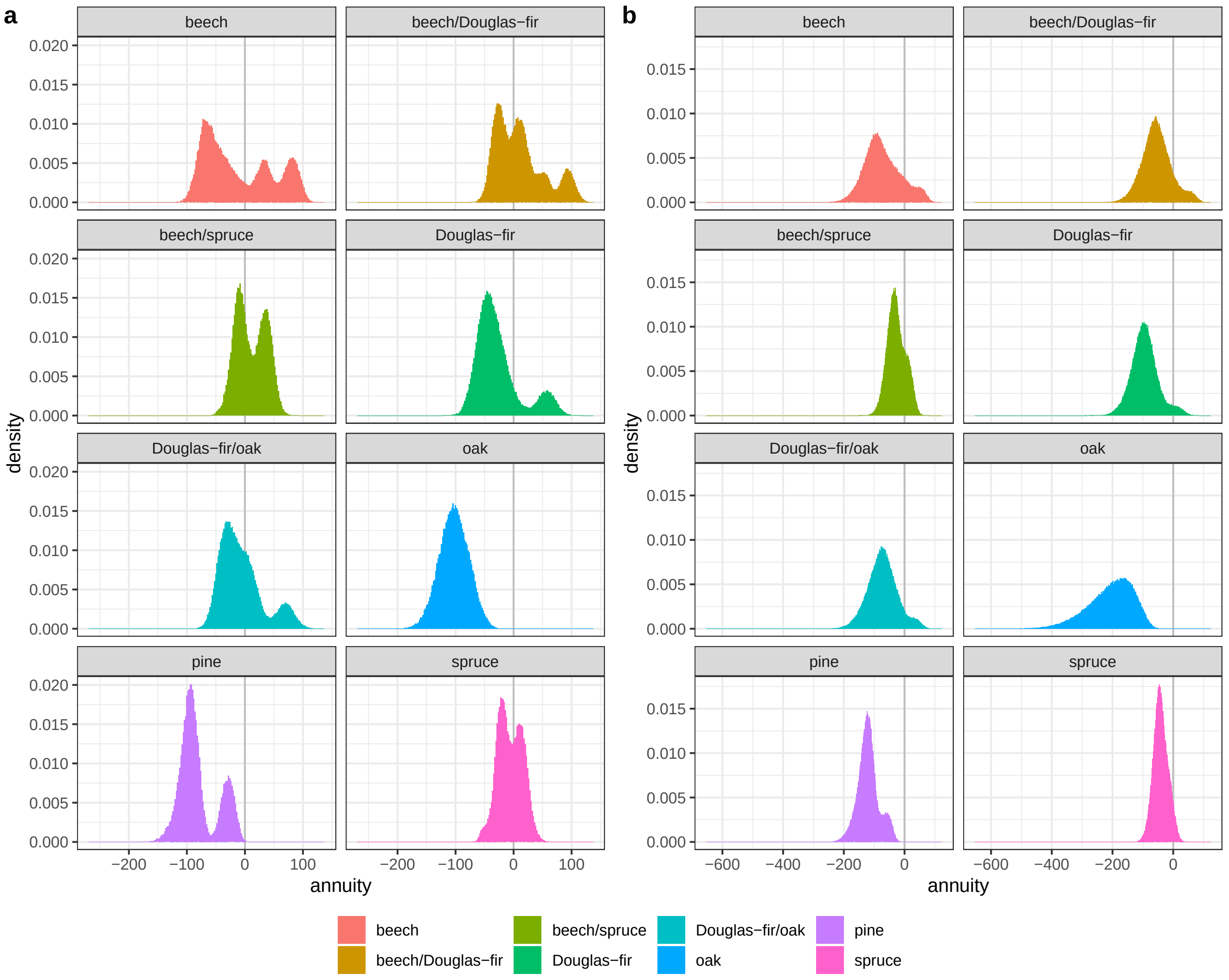

Figure A20. Densities of annual economic return (annuity) of the stand types based on the 10 000 repetitions of the Monte-Carlo simulation and combining the annuities of all planning units; for the extreme-event scenarios *none (0%)* (a) and *intensive (6%) - more events* (b).

A2.8 Characteristics of the forest enterprise under the optimal stand-type allocations

A2.8.1 Additional scenarios to Figure 6

top-down planning and larger events

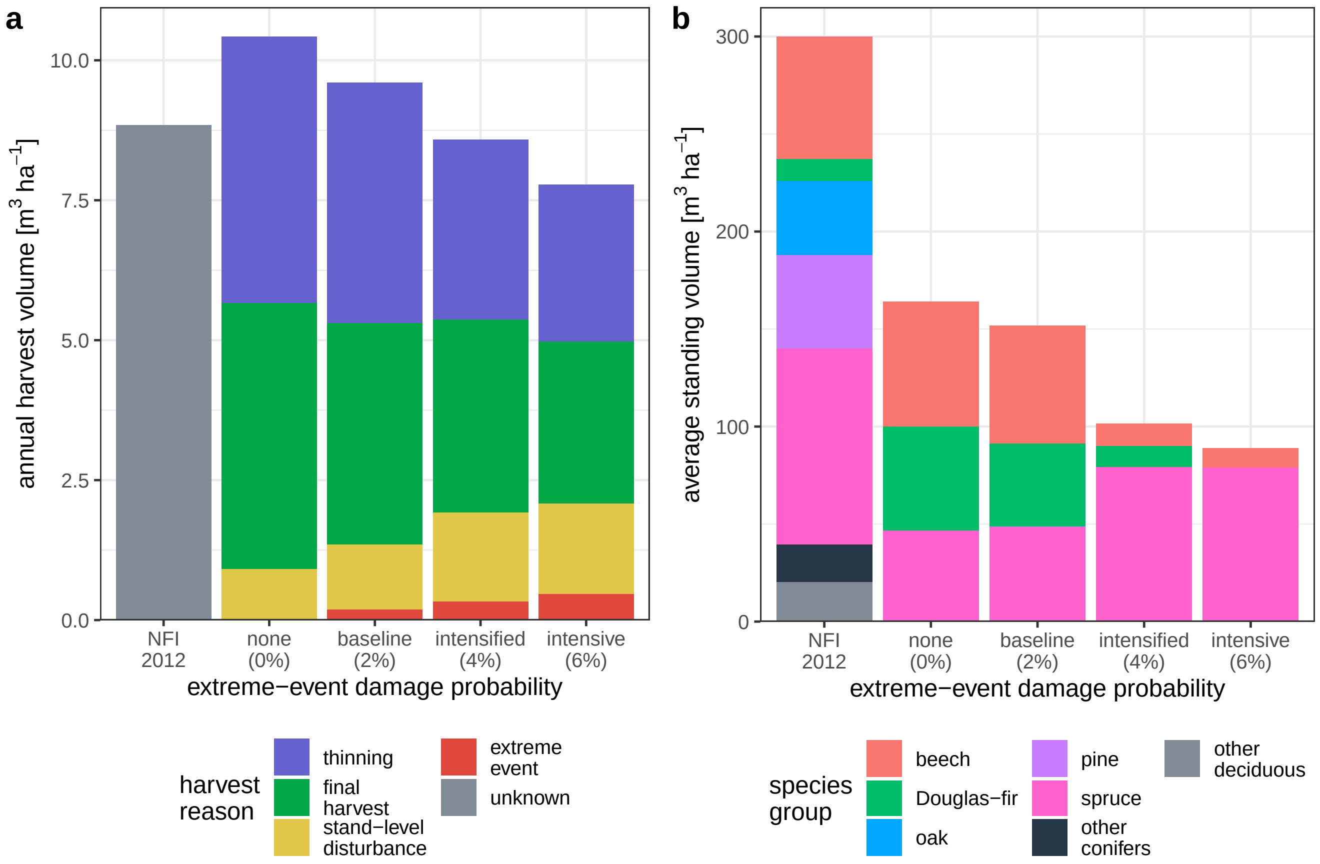

Figure A21. Consequences of the likely stand-type allocation under the risk-averse economic objective function for the region's forests. Comparison of the average of the simulated annual harvest volume (a) and standing volume (b) to the German NFI 2012 [10] (left bars). The values refer to top-down planning and increasing damage probabilities due to an increase in the size of extreme weather events (horizontal axis).

bottom-up planning and more events

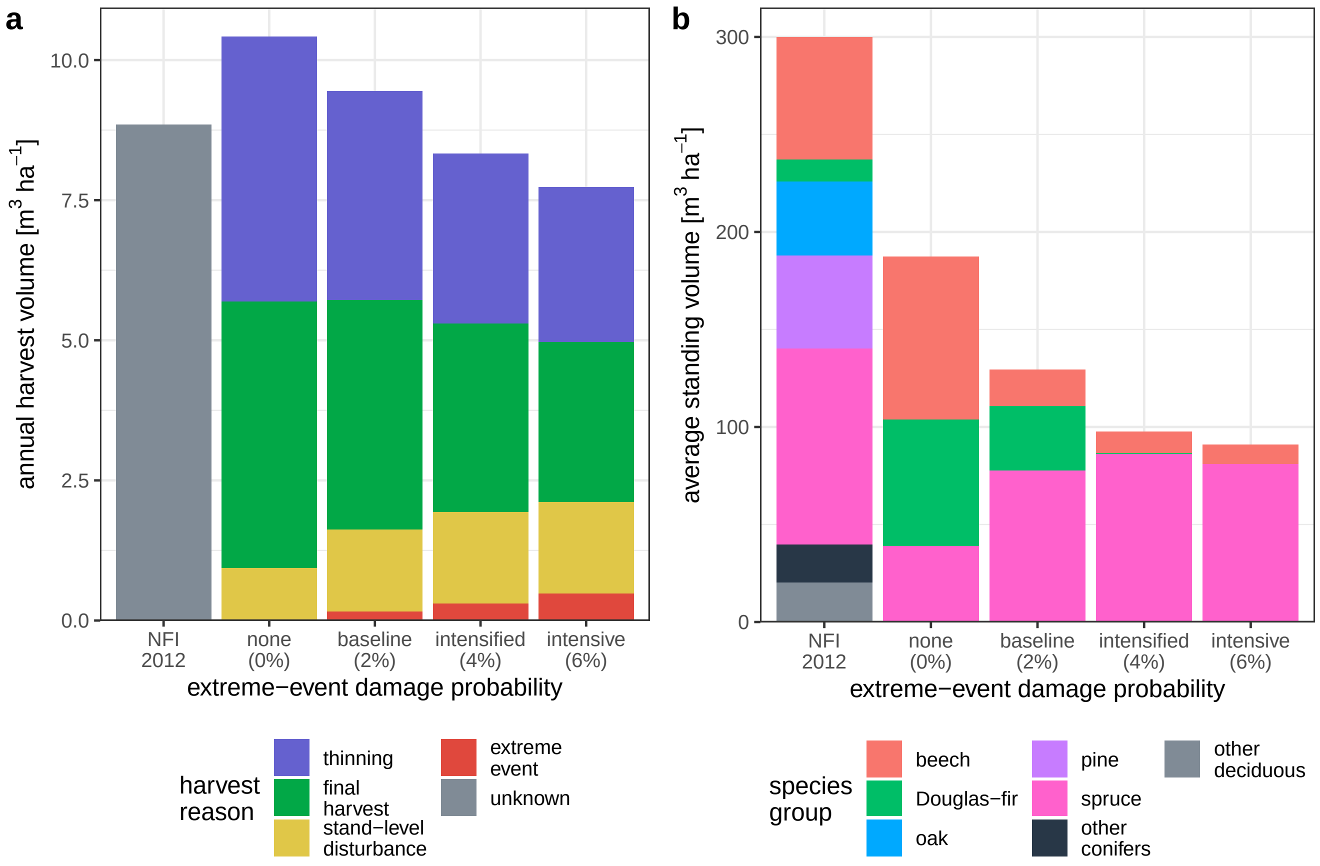

Figure A22. Consequences of the likely stand-type allocation under the risk-averse economic objective function for the region's forests. Comparison of the average of the simulated annual harvest volume (a) and standing volume (b) to the German NFI 2012 [10] (left bars). The values refer to bottom-up planning and increasing damage probabilities due to an increase in the number of extreme weather events (horizontal axis).

bottom-up planning and larger events

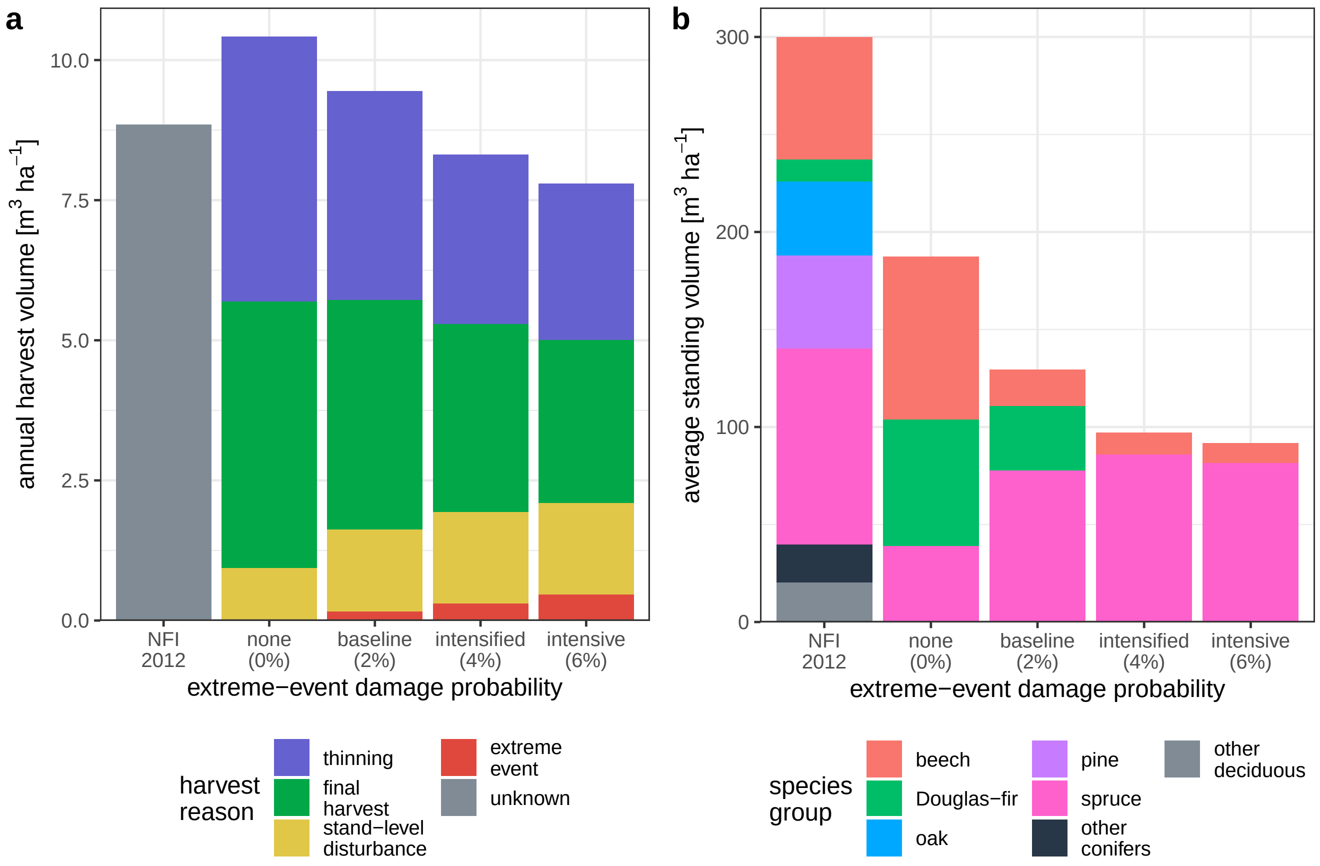

Figure A23. Consequences of the likely stand-type allocation under the risk-averse economic objective function for the region's forests. Comparison of the average of the simulated annual harvest volume (a) and standing volume (b) to the German NFI 2012 [10] (left bars). The values refer to bottom-up planning and increasing damage probabilities due to an increase in the size of extreme weather events (horizontal axis).

A2.8.2 age structure

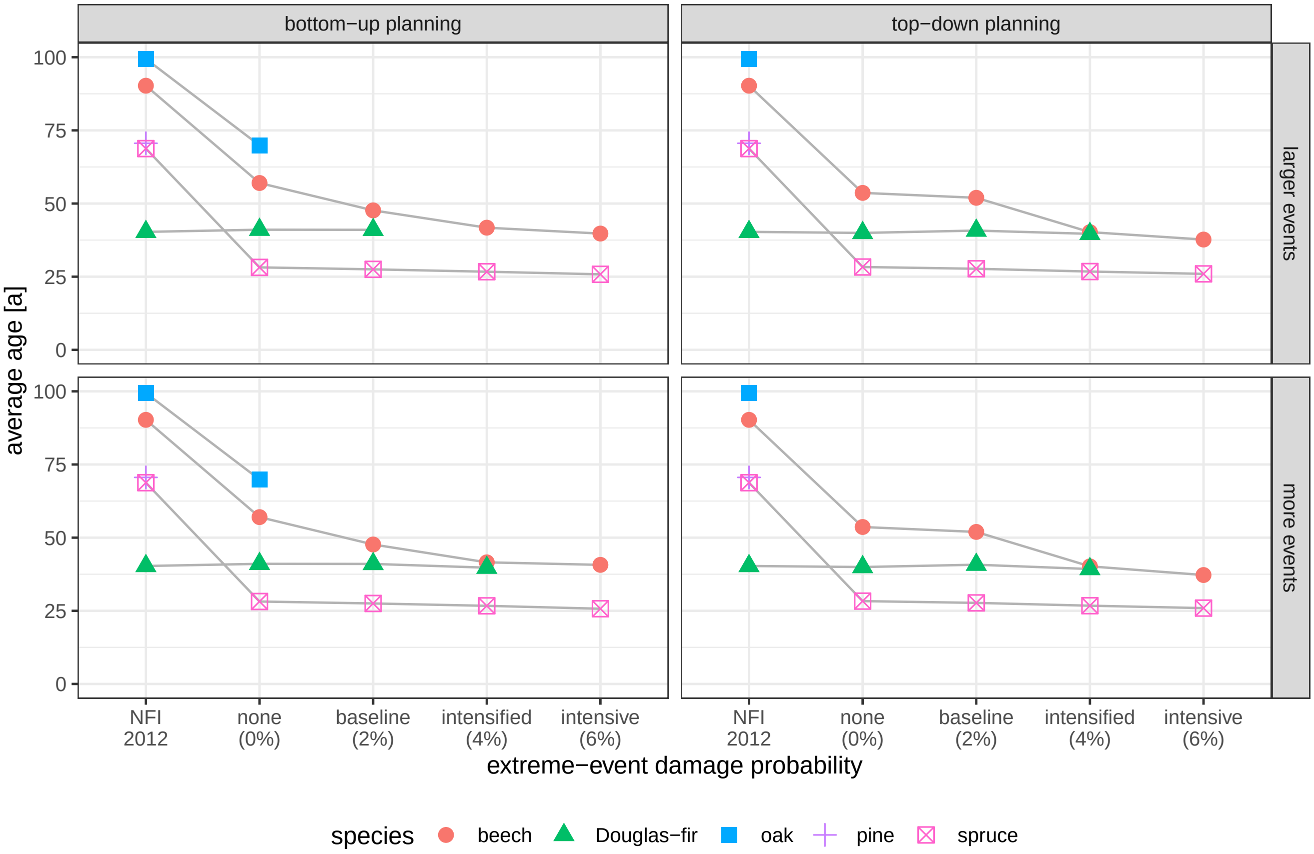

Figure A24. Average age of the tree species in the forest enterprise under the economically optimal stand-type allocation and their dependence on the planning perspective (panel columns) and extreme-event scenario (horizontal axis and panel rows), and comparison of the simulation-optimization results with the German NFI 2012[10].

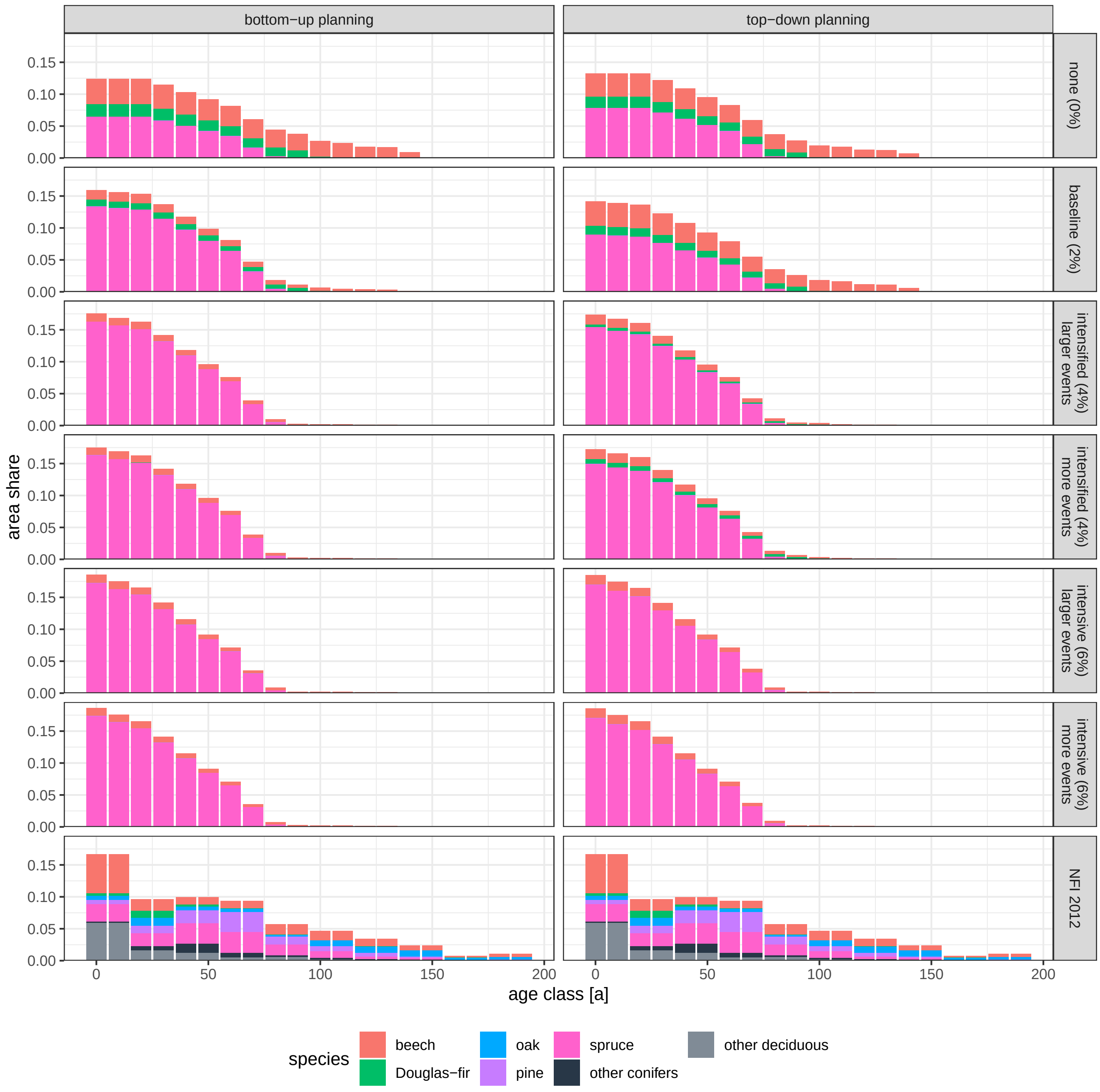

Figure A25. Area shares of the age classes per species at the enterprise level under the economically optimal stand-type allocation and their dependence on the planning perspective (panel columns) and extreme-event scenario (horizontal axis and panel rows), and comparison of the simulation-optimization results with the German NFI 2012 [10]. It should be noted that the area shares of age classes and species in the NFI were estimated with two different inventory methods. Regeneration is surveyed using concentric circular plots, while trees with a stem diameter of more than 7 cm are surveyed using angle count sampling. Since these concentric plots are covered by trees > 7 cm in multi-layered stands, the area shares sum up to more than 100 %. The area share above 100 % is the area share of regenerated stands under mature forests.

A2.8.3 Harvested and disturbed area

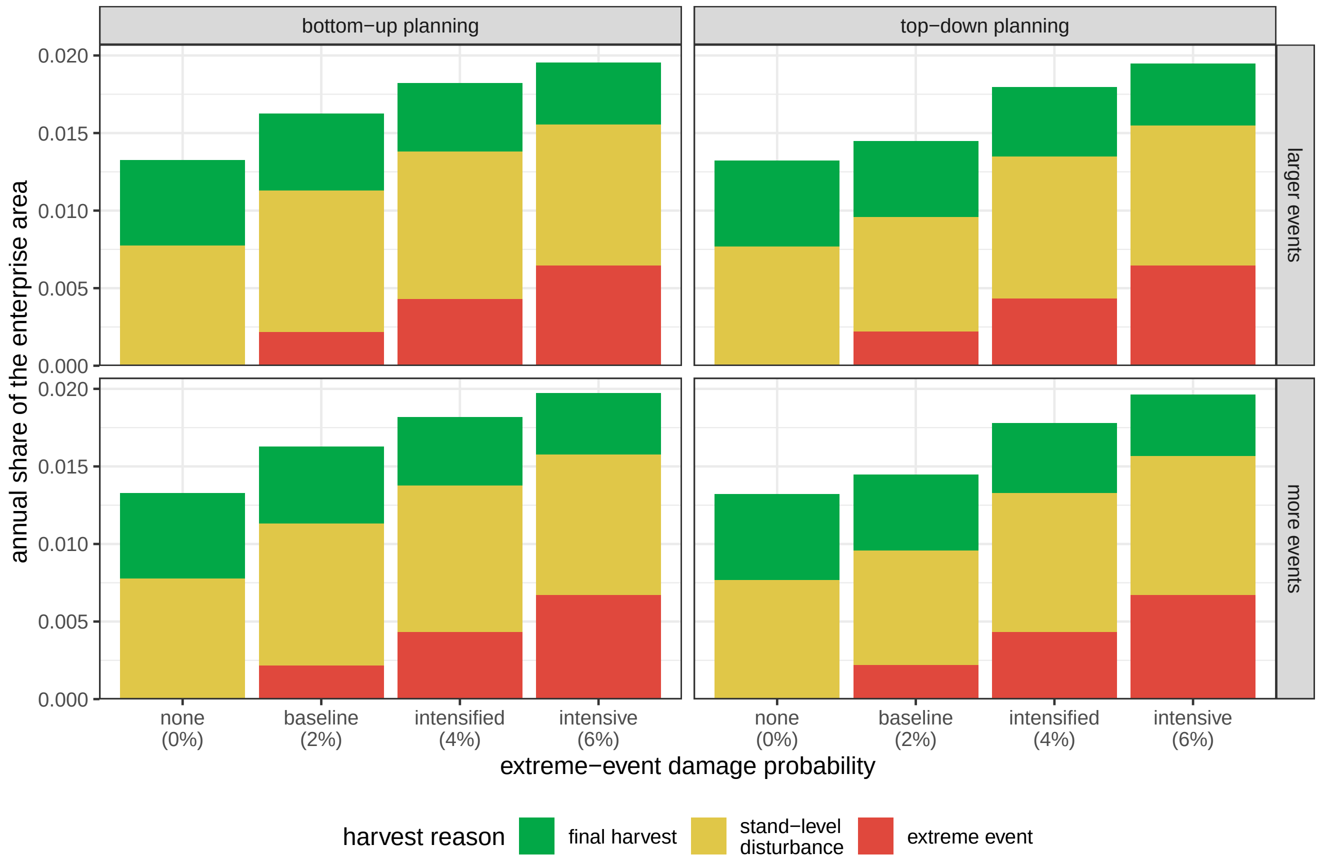

Figure A26. Annual share of the enterprise area that is harvested or disturbed under the economically optimal stand-type allocation and its dependence on the planning perspective (panel columns) and extreme-event scenario (horizontal axis and panel rows). (Such data was not available from the German NFI.)

A2.8.4 Mean diameter

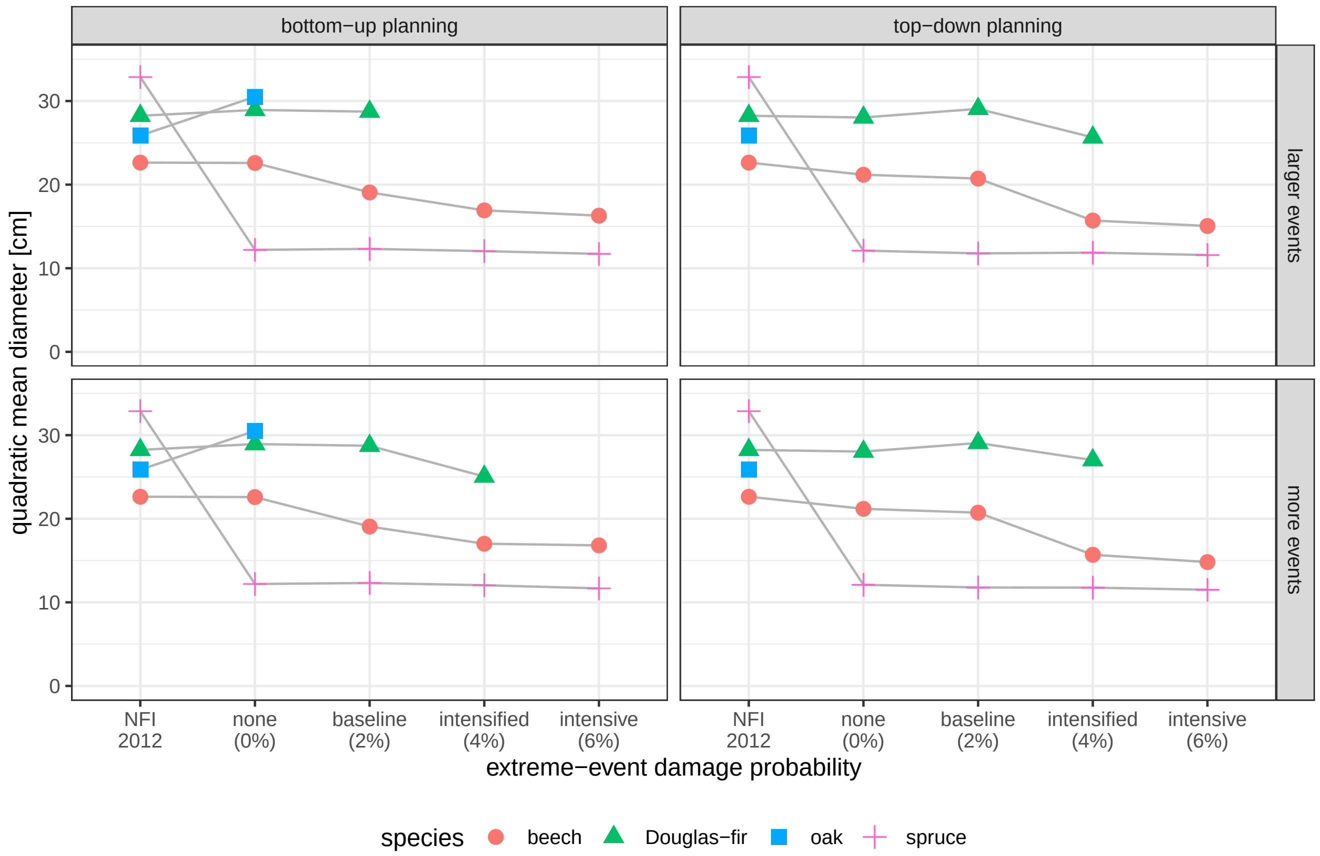

Figure A27. Quadratic mean diameters at breast height of the tree species in the forest enterprise under the economically optimal stand-type allocation and their dependence on the planning perspective (panel columns) and extreme-event scenario (horizontal axis and panel rows), and comparison of the simulation-optimization results with the German NFI 2012[10].

A2.8.5 Annual harvest volume

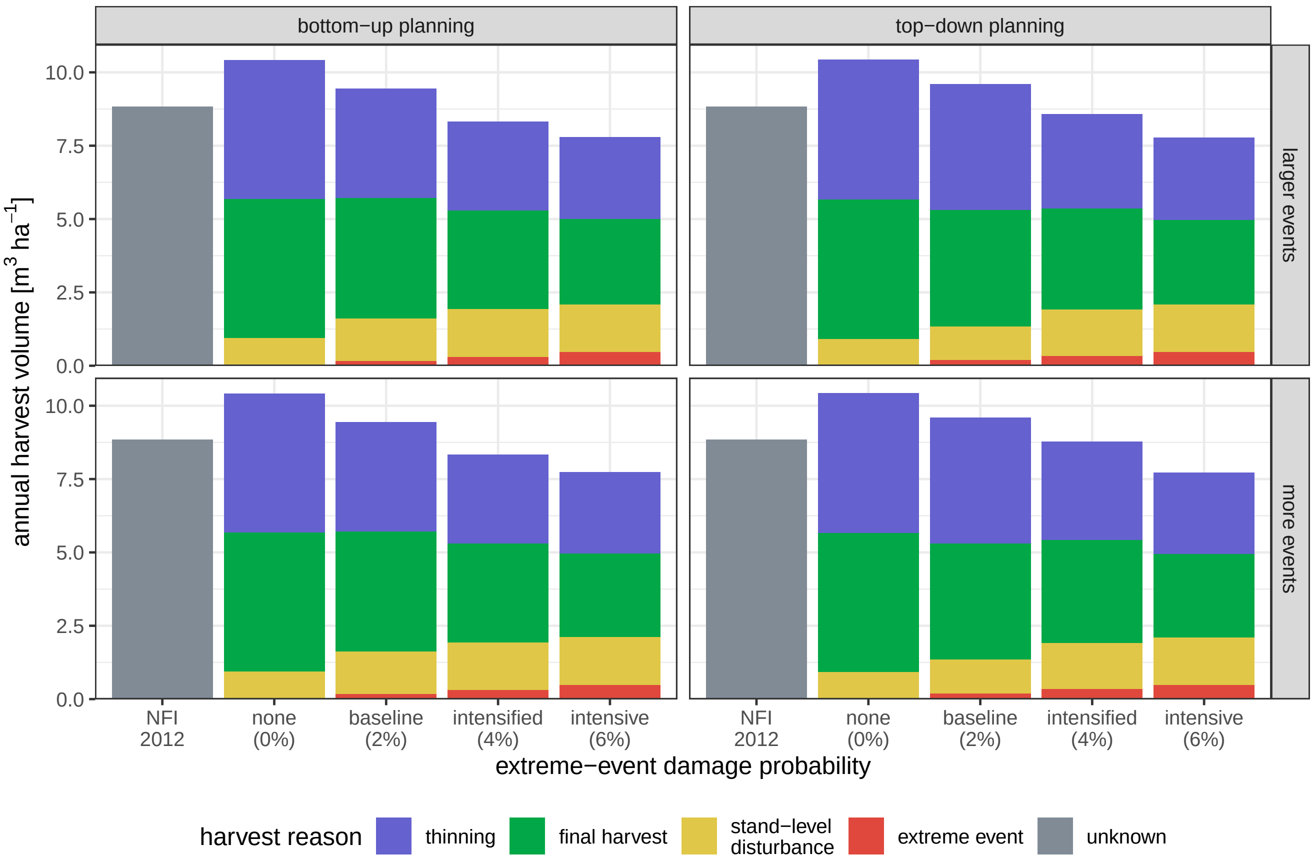

Figure A28. Average annual harvest volume in the forest enterprise under the economically optimal stand-type allocation and its dependence on the planning perspective (panel columns) and extreme-event scenario (horizontal axis and panel rows), and comparison of the simulation-optimization results with the German NFI 2012[10].

A2.8.6 Species diversity

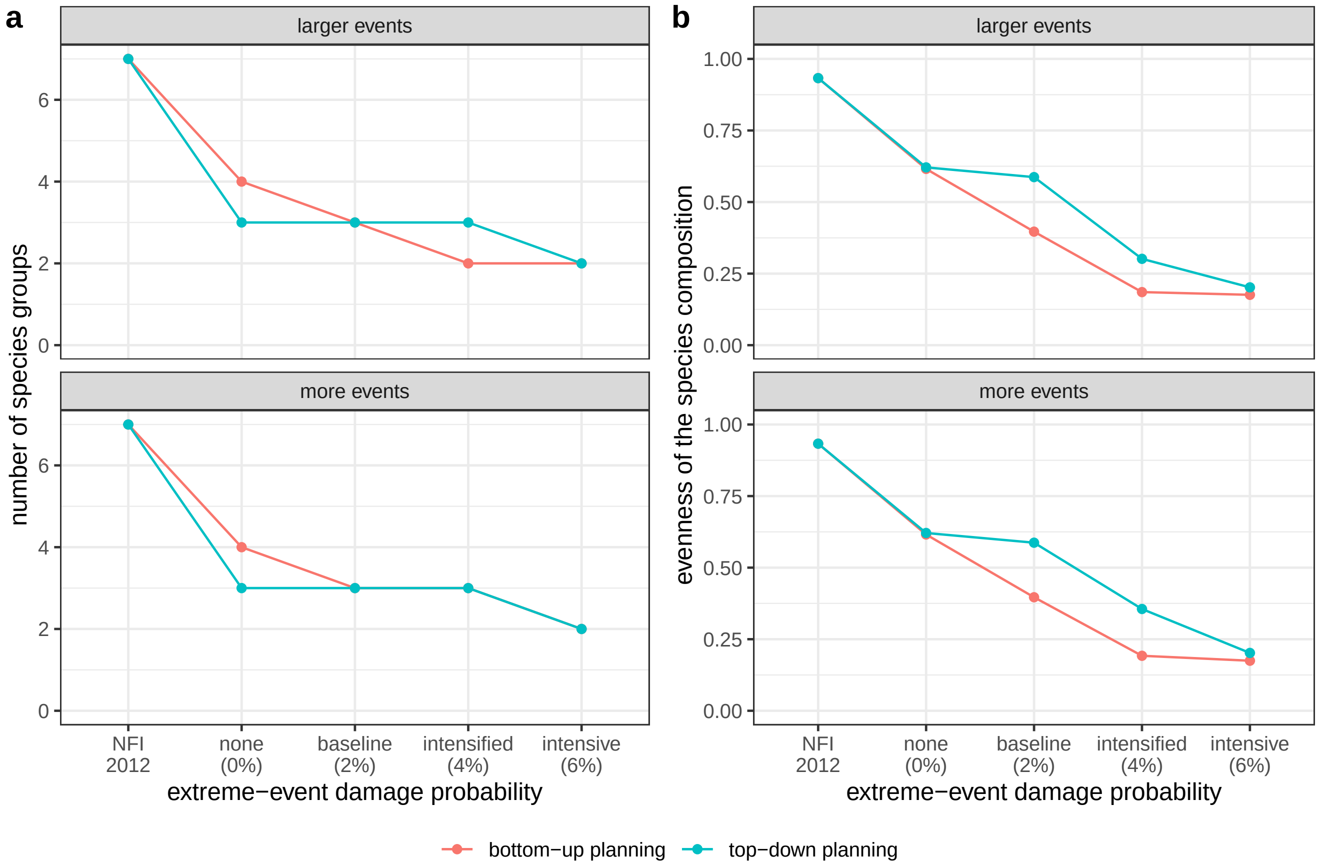

Figure A29. Diversity of tree-species groups at the enterprise level in terms of their number (a) and evenness of their composition (b) under the economically optimal stand-type allocation and their dependence on the planning perspective (colors) and extreme-event scenario (horizontal axis and panels), and comparison of the simulation-optimization results with the German NFI 2012[10].

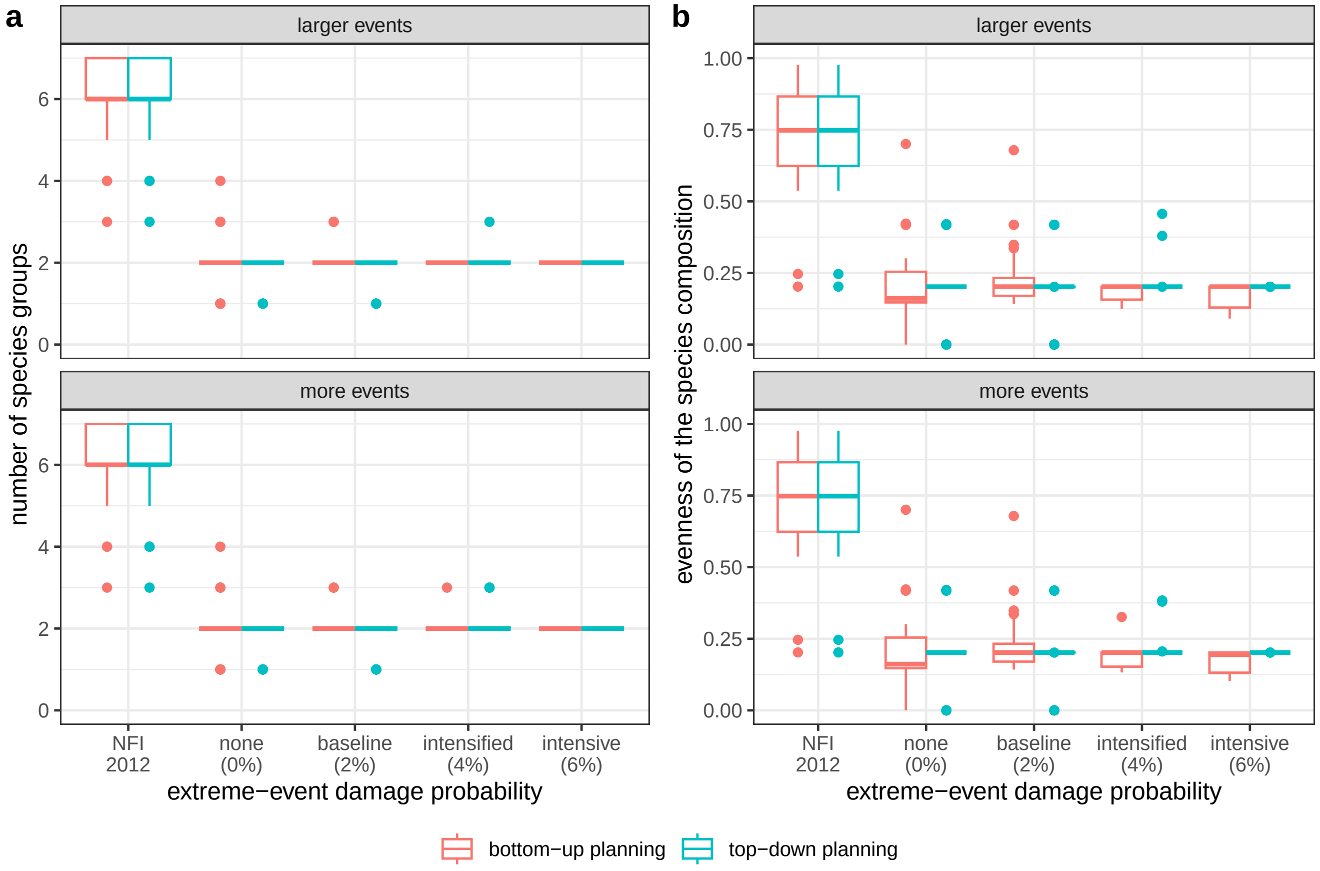

Figure A30. Diversity of tree-species groups in the 24 individual planning units of enterprise in terms of the number of species groups (a) and evenness of their composition (b) under the economically optimal stand-type allocation and their dependence on the planning perspective (colors) and extreme-event scenario (horizontal axis and panels), and comparison of the simulation-optimization results with the German NFI 2012 [10].

A2.9 Sensitivity analysis

A2.9.1 Minimum hazard rate due to extreme weather events

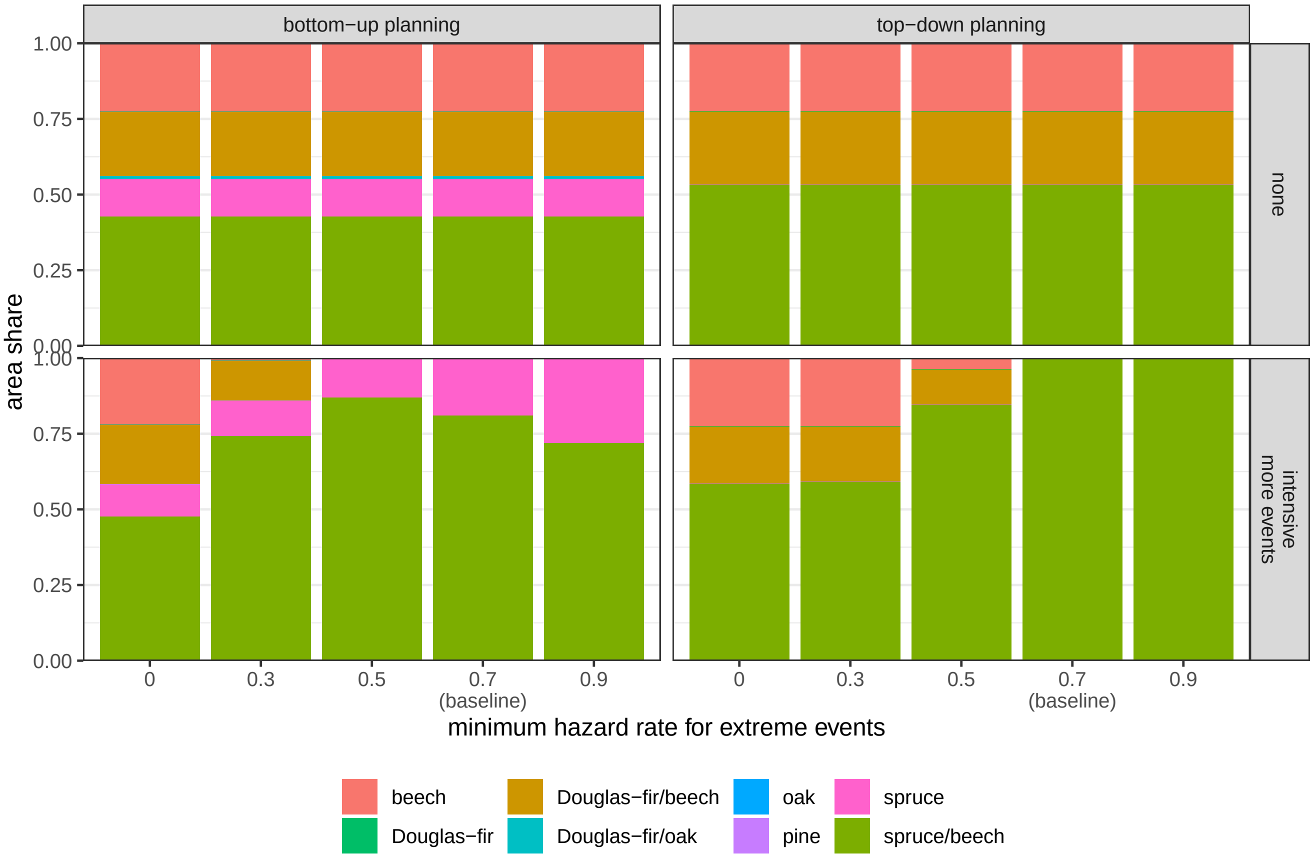

Figure A31. Optimal stand-type composition of the forest enterprise (γ-diversity) for different planning perspectives (panel columns) and two extreme-event scenarios (panel rows) dependent on the minimum hazard rate (h_{min} in Eq. (A7)), i.e., minimum probability of damage to the forest stand, in case of an extreme weather event at this location.

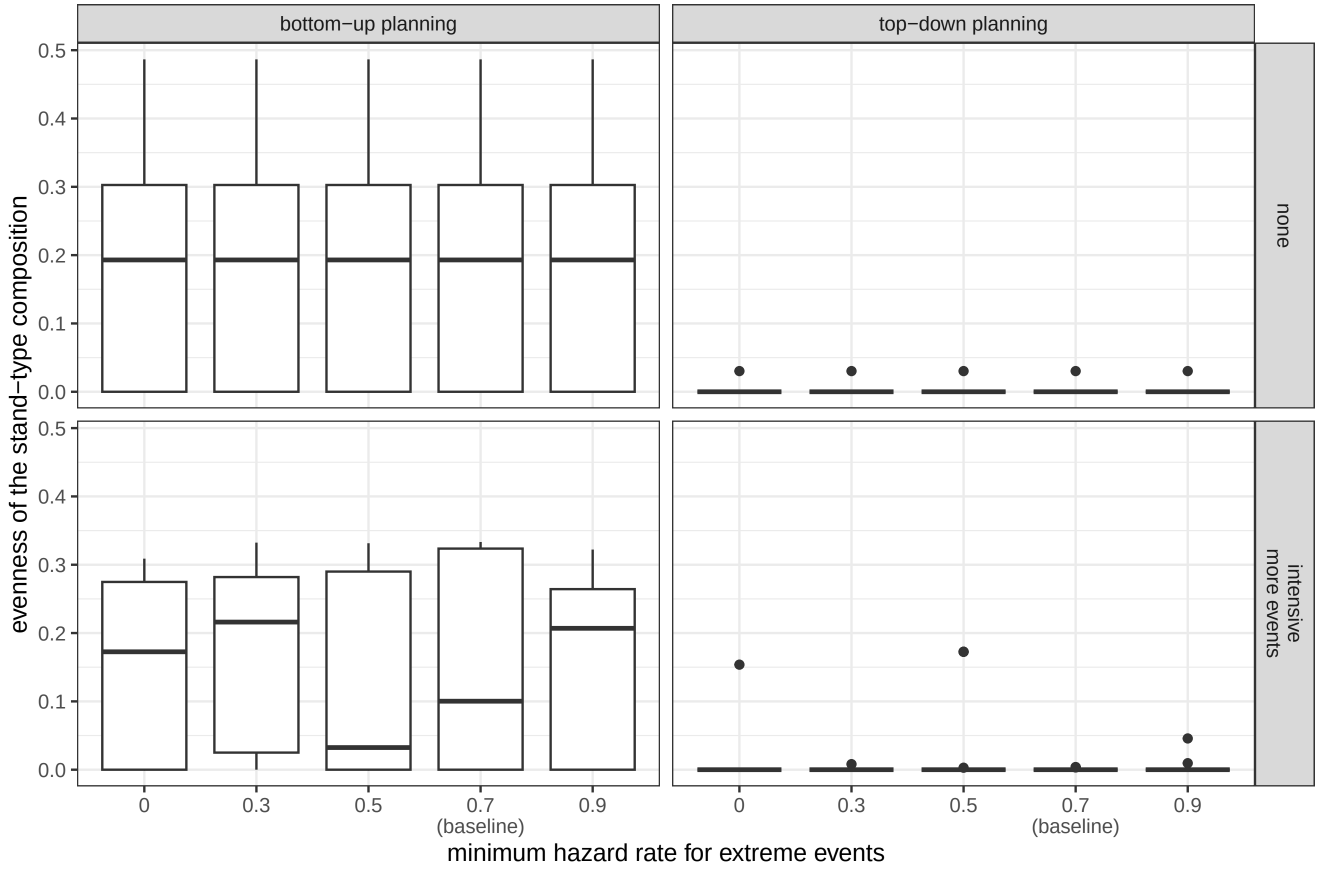

Figure A32. Evenness of the optimal stand-type composition of the forest enterprise (γ-diversity) for different planning perspectives (panel columns) and two extreme-event scenarios (panel rows) dependent on the minimum hazard rate (h_{min} in Eq. (A7)), i.e., minimum probability of damage to the forest stand, in case of an extreme weather event at this location.

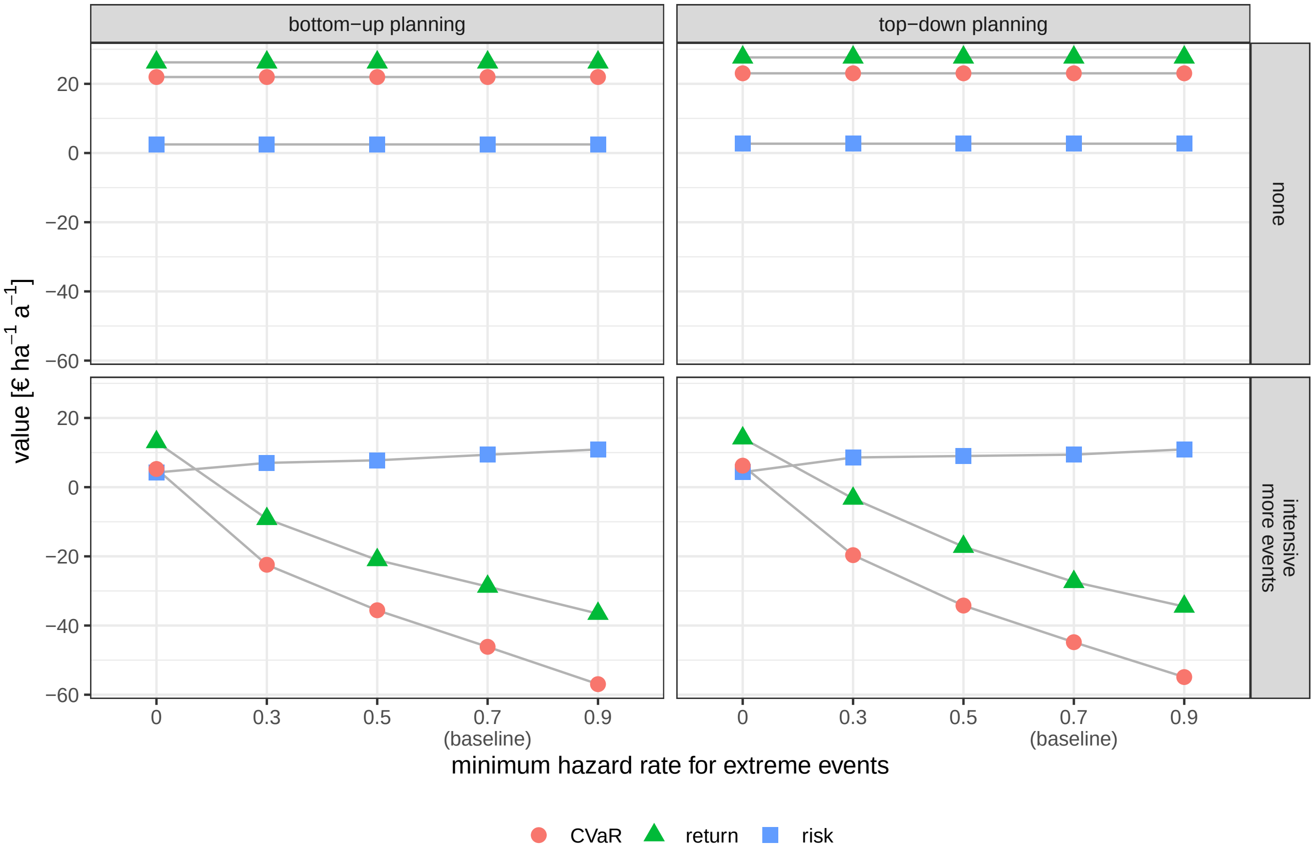

Figure A33. Economic variables of the forest enterprise under the optimal stand-type composition for different planning perspectives (panel columns) and two extreme-event scenarios (panel rows) dependent on the minimum hazard rate (h_{min} in Eq. (A7)), i.e., minimum probability of damage to the forest stand, in case of an extreme weather event at this location. CVaR: Conditional Value at Risk (10 %-quantile), return: expected annuity, risk: standard deviation of the annuity.

A2.9.2 Maximum hazard rate due to extreme weather events

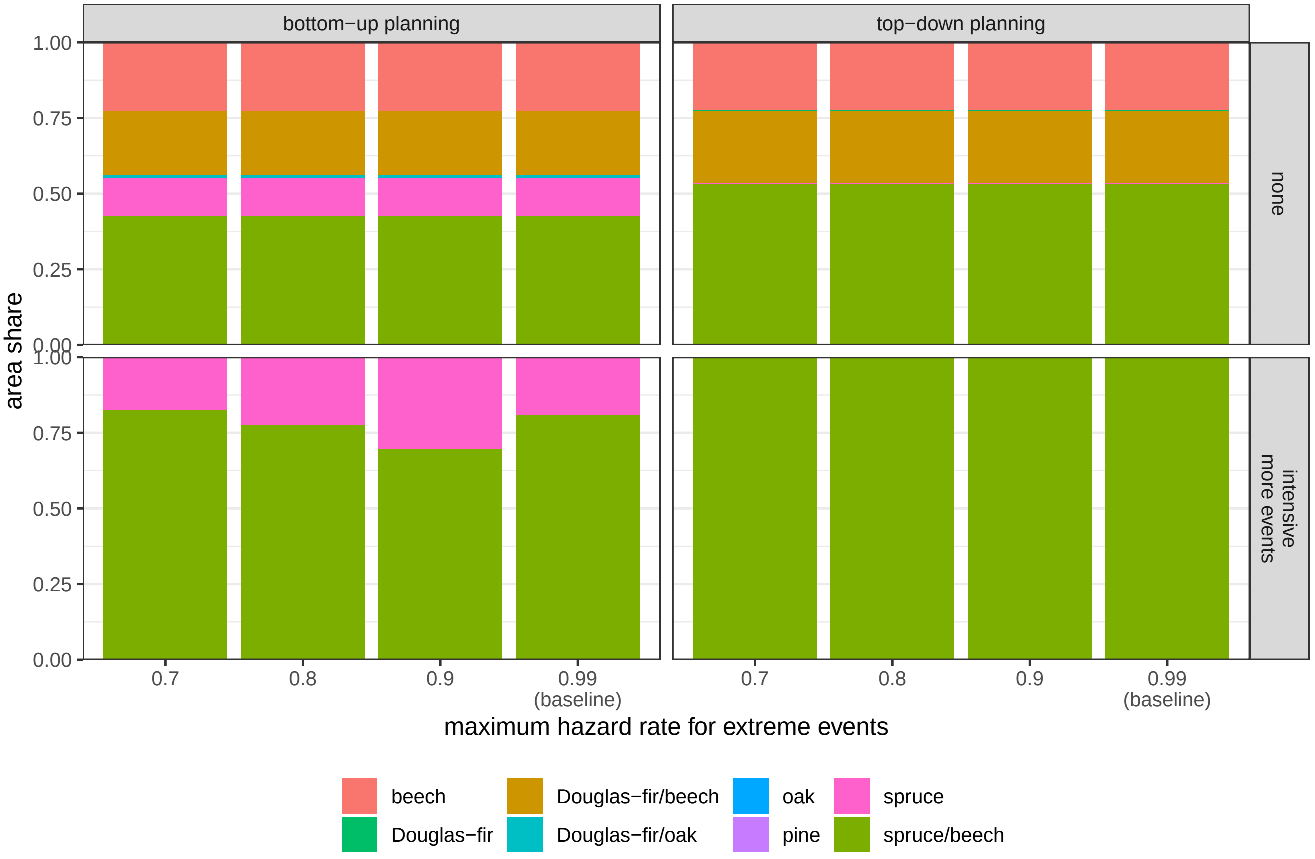

Figure A34. Optimal stand-type composition of the forest enterprise (γ-diversity) for different planning perspectives (panel columns) and two extreme-event scenarios (panel rows) dependent on the maximum hazard rate (h_{max} in Eq. (A7)), i.e., maximum probability of damage to the forest stand, in case of an extreme weather event at this location.

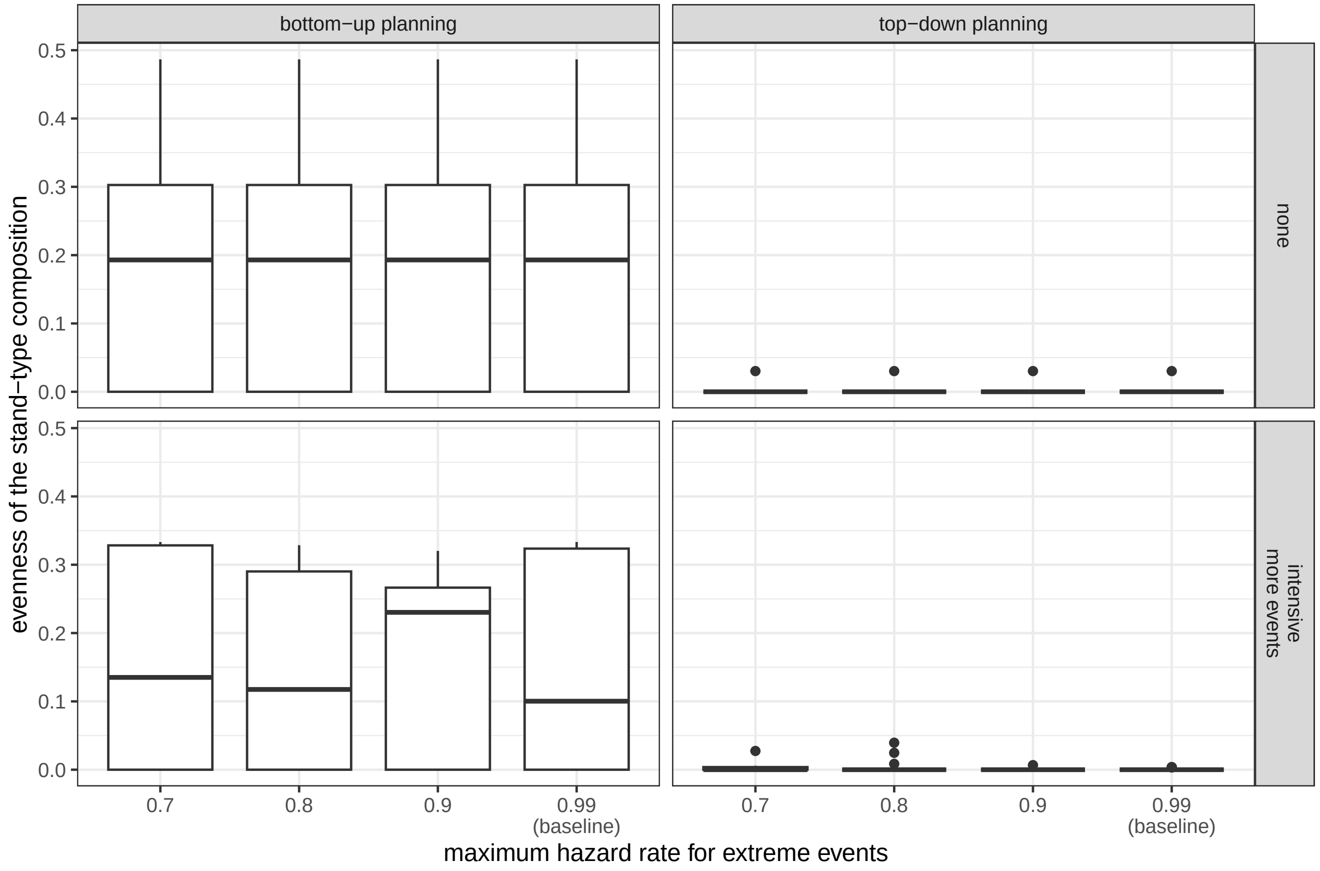

Figure A35. Evenness of the optimal stand-type composition of the forest enterprise (γ-diversity) for different planning perspectives (panel columns) and two extreme-event scenarios (panel rows) dependent on the maximum hazard rate (h_{max} in Eq. (A7)), i.e., maximum probability of damage to the forest stand, in case of an extreme weather event at this location.

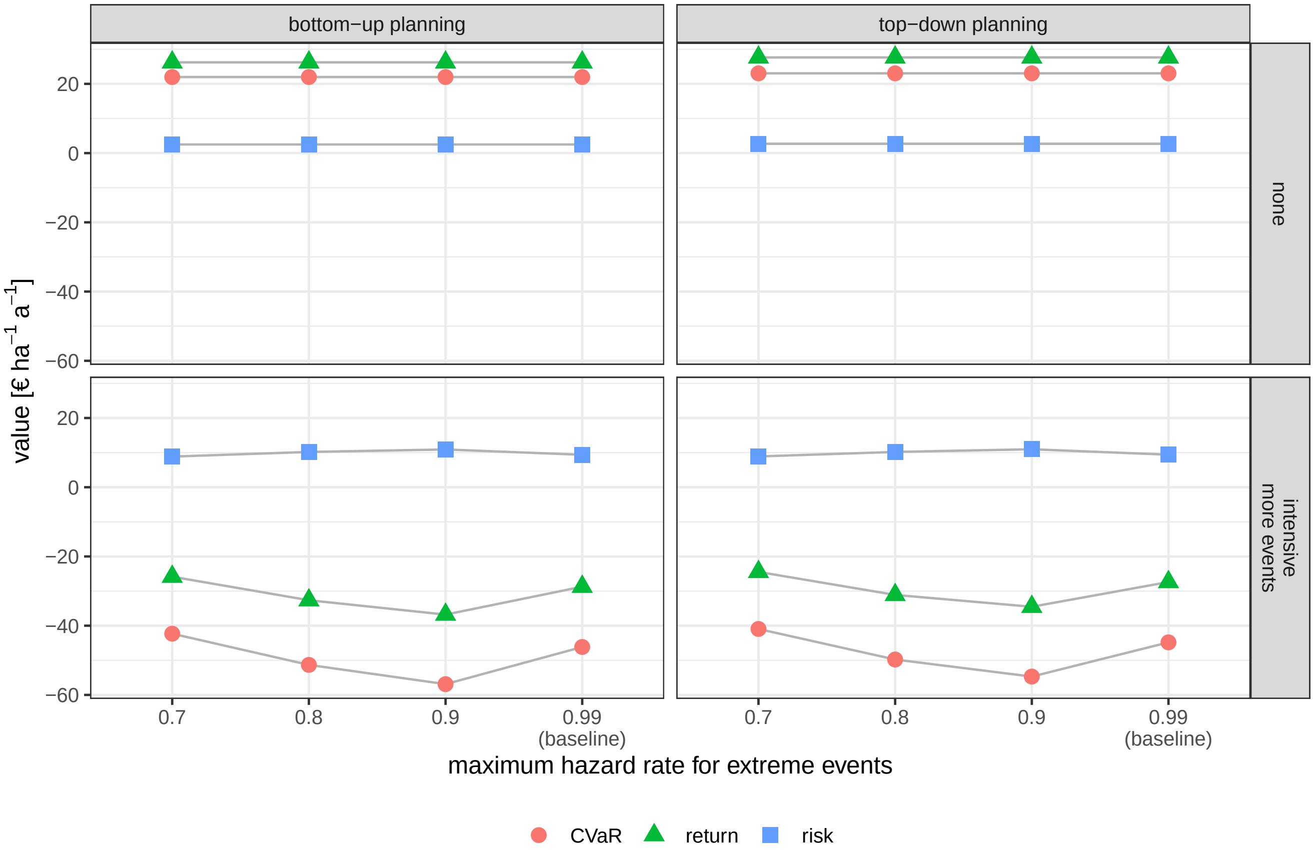

Figure A36. Economic variables of the forest enterprise under the optimal stand-type composition for different planning perspectives (panel columns) and two extreme-event scenarios (panel rows) dependent on the maximum hazard rate (h_{max} in Eq. (A7)), i.e., maximum probability of damage to the forest stand, in case of an extreme weather event at this location. CVaR: Conditional Value at Risk (10 %-quantile), return: expected annuity, risk: standard deviation of the annuity.

A2.9.3 Planting costs

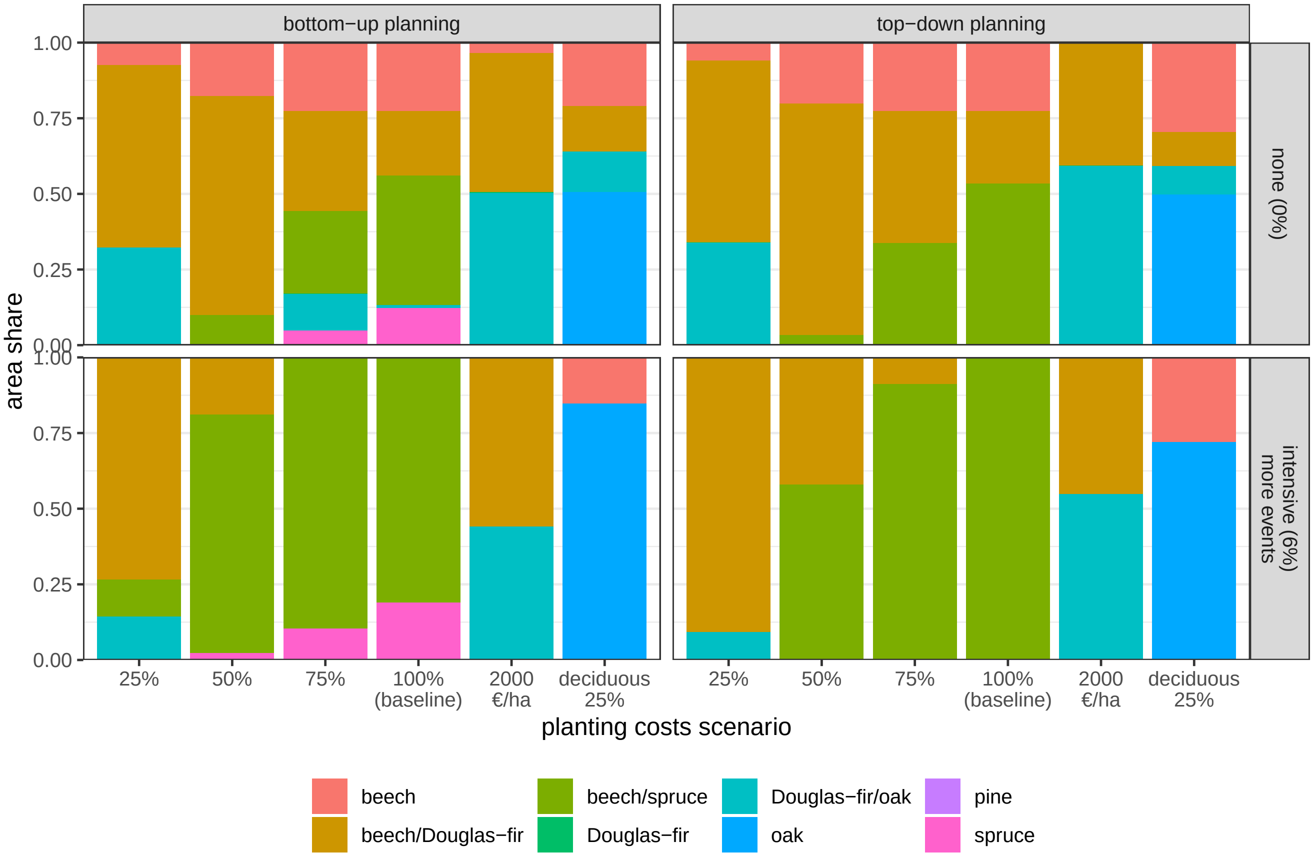

Figure A37. Optimal stand-type composition of the forest enterprise (γ-diversity) for different planning perspectives (panel columns) and two extreme-event scenarios (panel rows) dependent on different scenarios for the planting cost. The planting costs were reduced to 25 %, 50 % and 75 % as compared to the baseline scenario (Tab. A8); in 'deciduous: 25 %' we reduced only the planting costs of deciduous species, reflecting a typical type of funding policy in the study region; in the last scenario, we used the same planting costs for all species (2000 € ha^{-1}).

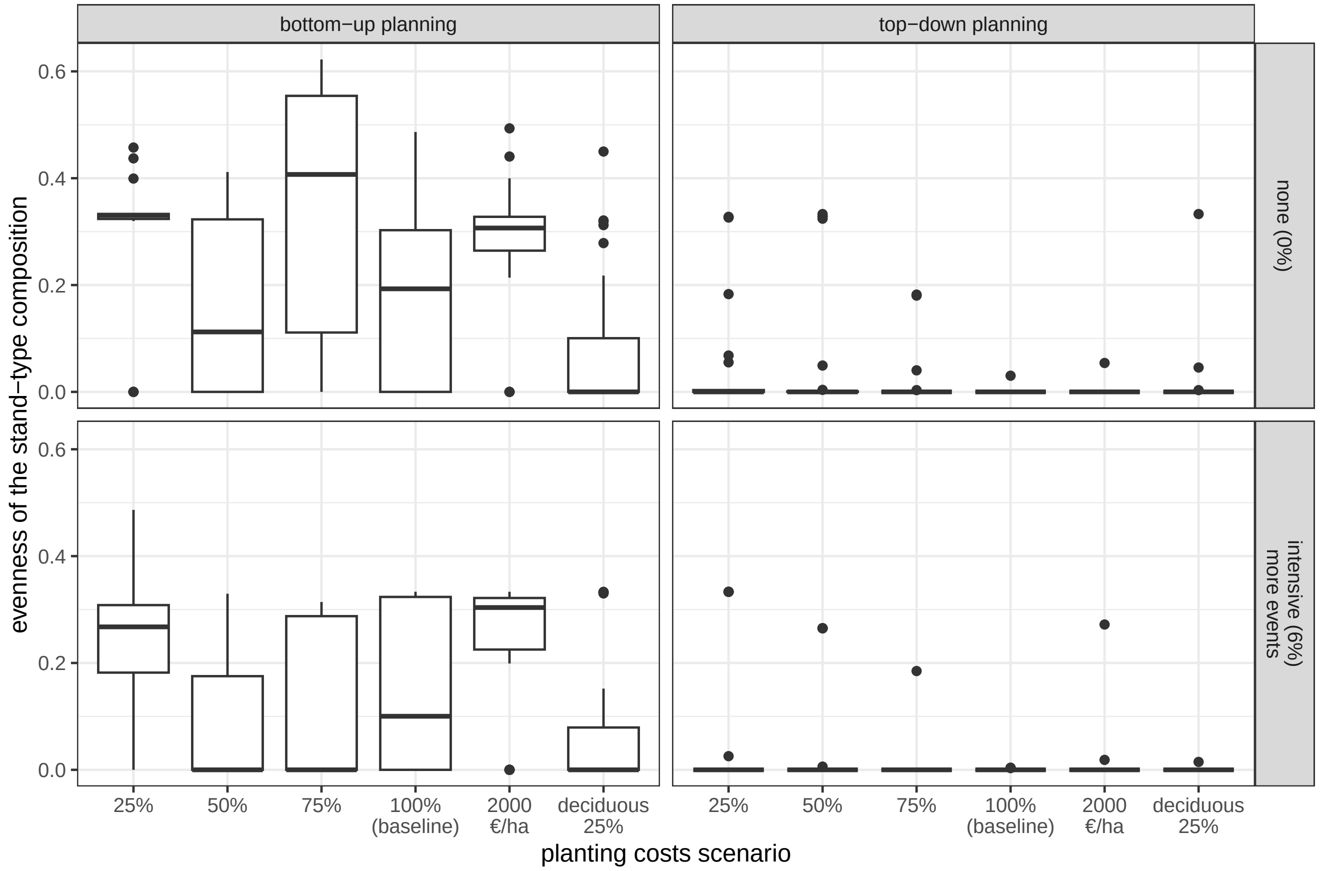

Figure A38. Evenness of the optimal stand-type composition of the forest enterprise (γ-diversity) for different planning perspectives (panel columns) and two extreme-event scenarios (panel rows) dependent on different scenarios for the planting cost. The planting costs were reduced to 25 %, 50 % and 75 % as compared to the baseline scenario (Tab. A8); in 'deciduous: 25 %' we reduced only the planting costs of deciduous species, reflecting a typical type of funding policy in the study region; in the last scenario, we used the same planting costs for all species (2000 € ha^{-1}).

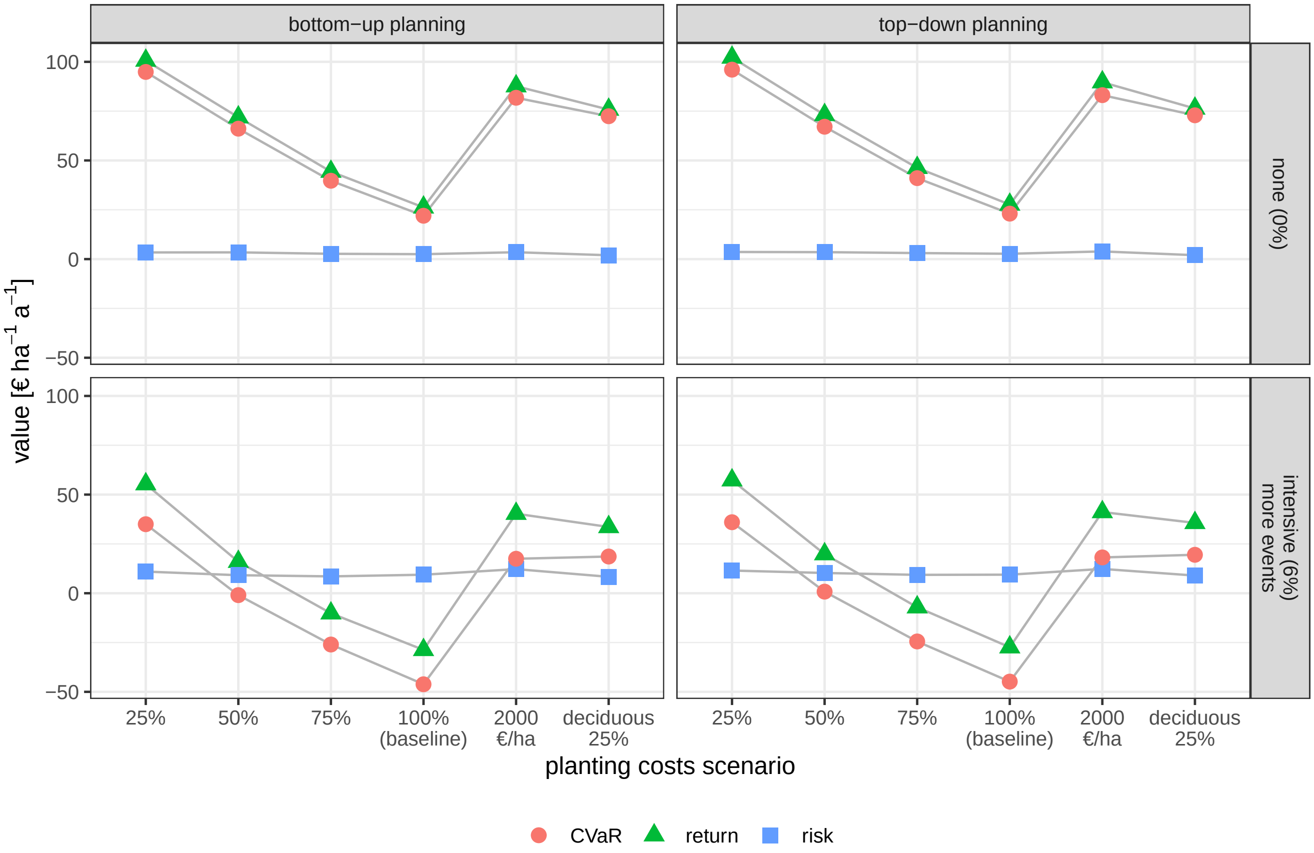

Figure A39. Economic variables of the forest enterprise under the optimal stand-type composition for different planning perspectives (panel columns) and two extreme-event scenarios (panel rows) dependent on different scenarios for the planting cost. The planting costs were reduced to 25 %, 50 % and 75 % as compared to the baseline scenario (Tab. A8); in 'deciduous: 25 %' we reduced only the planting costs of deciduous species, reflecting a typical type of funding policy in the study region; in the last scenario, we used the same planting costs for all species (2000 € ha^{-1}). CVaR: Conditional Value at Risk (10 %-quantile), return: expected annuity, risk: standard deviation of the annuity.

A2.9.4 Interest rate

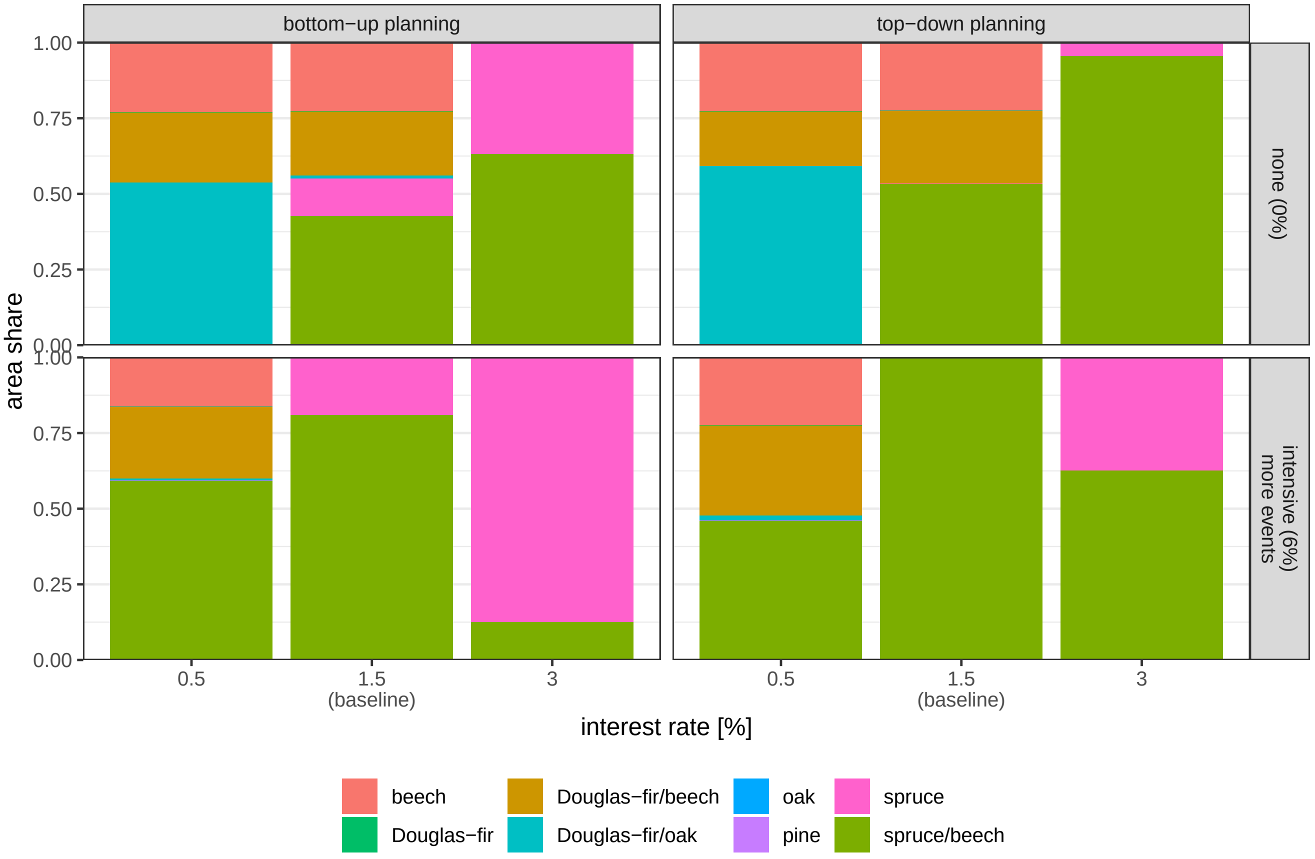

Figure A40. Optimal stand-type composition of the forest enterprise (γ-diversity) for different planning perspectives (panel columns) and two extreme-event scenarios (panel rows) dependent on the interest rate applied in the calculation of the economic indicators.

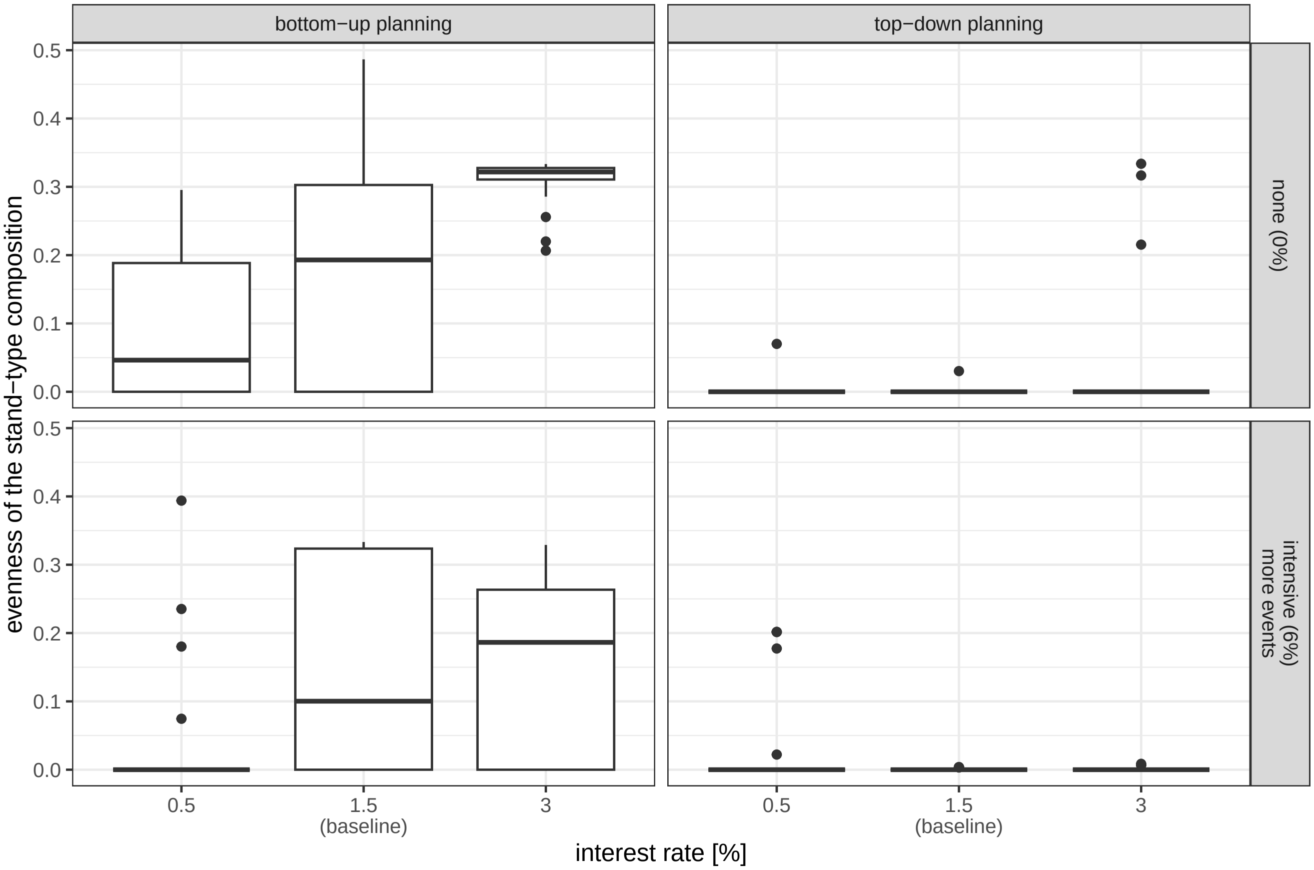

Figure A41. Evenness of the optimal stand-type composition of the forest enterprise (γ-diversity) for different planning perspectives (panel columns) and two extreme-event scenarios (panel rows) dependent on the interest rate applied in the calculation of the economic indicators.

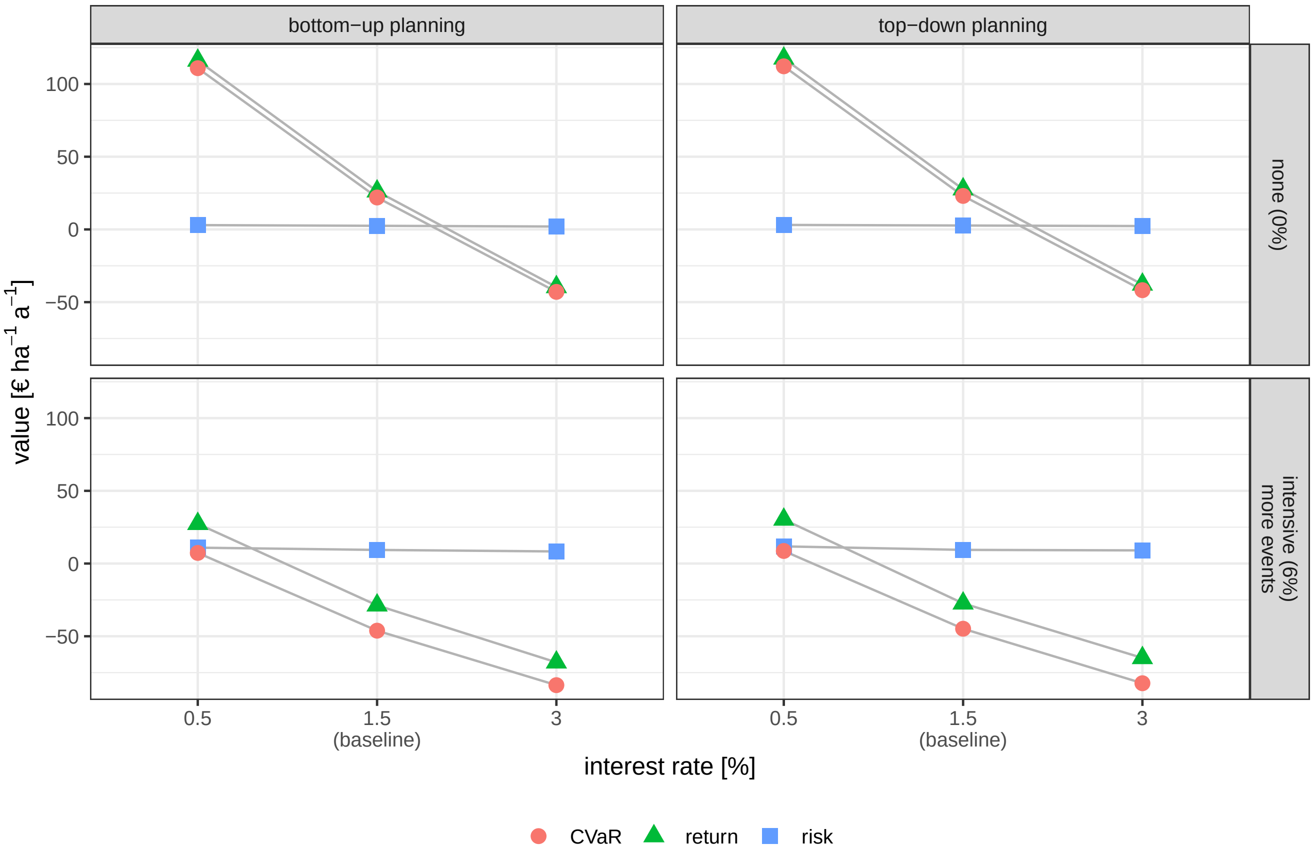

Figure A42. Economic variables of the forest enterprise under the optimal stand-type composition for different planning perspectives (panel columns) and two extreme-event scenarios (panel rows) dependent on the interest rate applied in the calculation of the economic indicators. CVaR: Conditional Value at Risk (10 %-quantile), return: expected annuity, risk: standard deviation of the annuity.

A2.9.5 Internal supply effect on wood prices

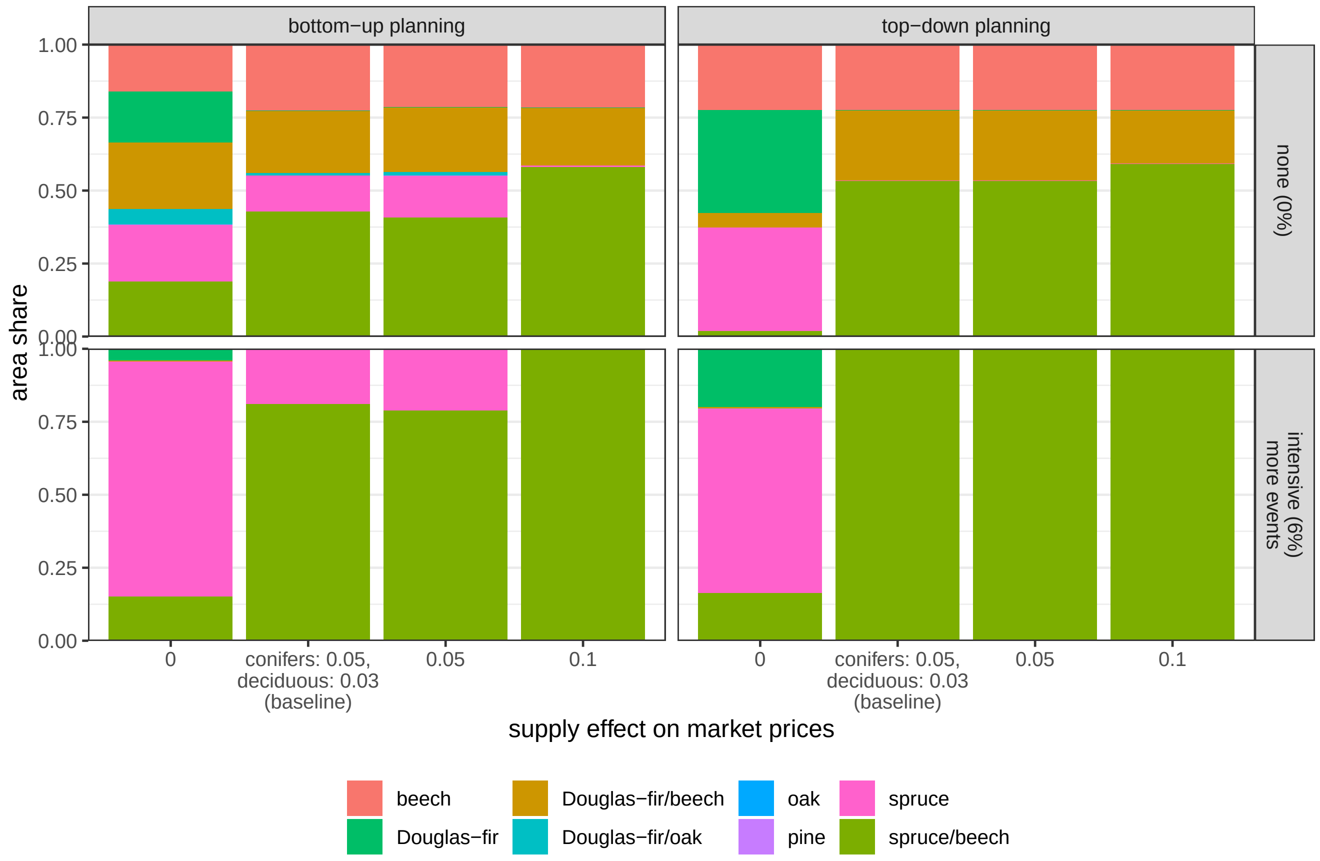

Figure A43. Optimal stand-type composition of the forest enterprise (γ-diversity) for different planning perspectives (panel columns) and two extreme-event scenarios (panel rows) dependent on the price reduction on the regional market due to a higher wood supply (see Eq. (11), (12)).

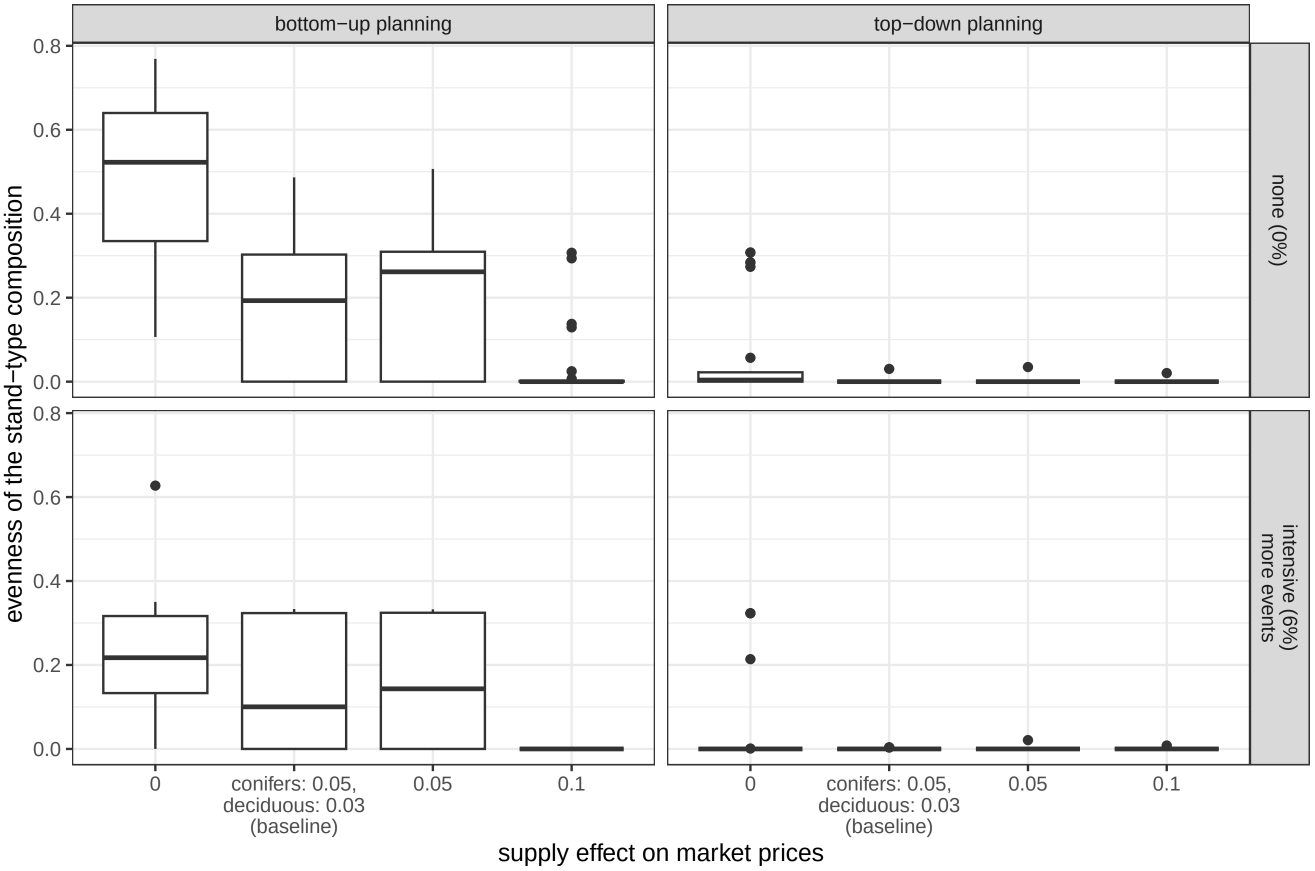

Figure A44. Evenness of the optimal stand-type composition of the forest enterprise (γ-diversity) for different planning perspectives (panel columns) and two extreme-event scenarios (panel rows) dependent on the price reduction on the regional market due to a higher wood supply (see Eq. (11), (12)).

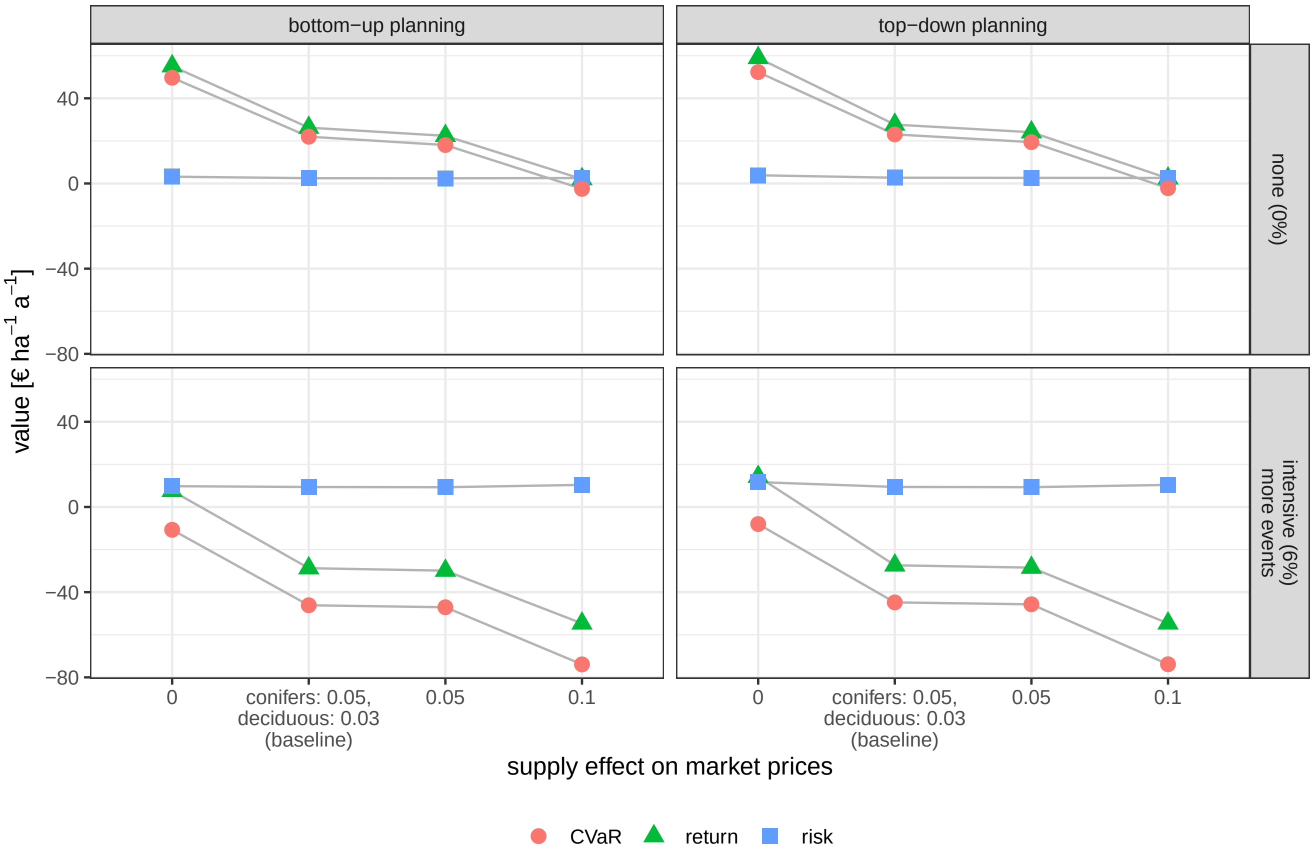

Figure A45. Economic variables of the forest enterprise under the optimal stand-type composition for different planning perspectives (panel columns) and two extreme-event scenarios (panel rows) dependent on the price reduction on the regional market due to a higher wood supply (see Eq. (11), (12)). CVaR: Conditional Value at Risk (10 %-quantile), return: expected annuity, risk: standard deviation of the annuity.

A2.9.6 Reduction in wood revenues due to quality losses of damaged wood

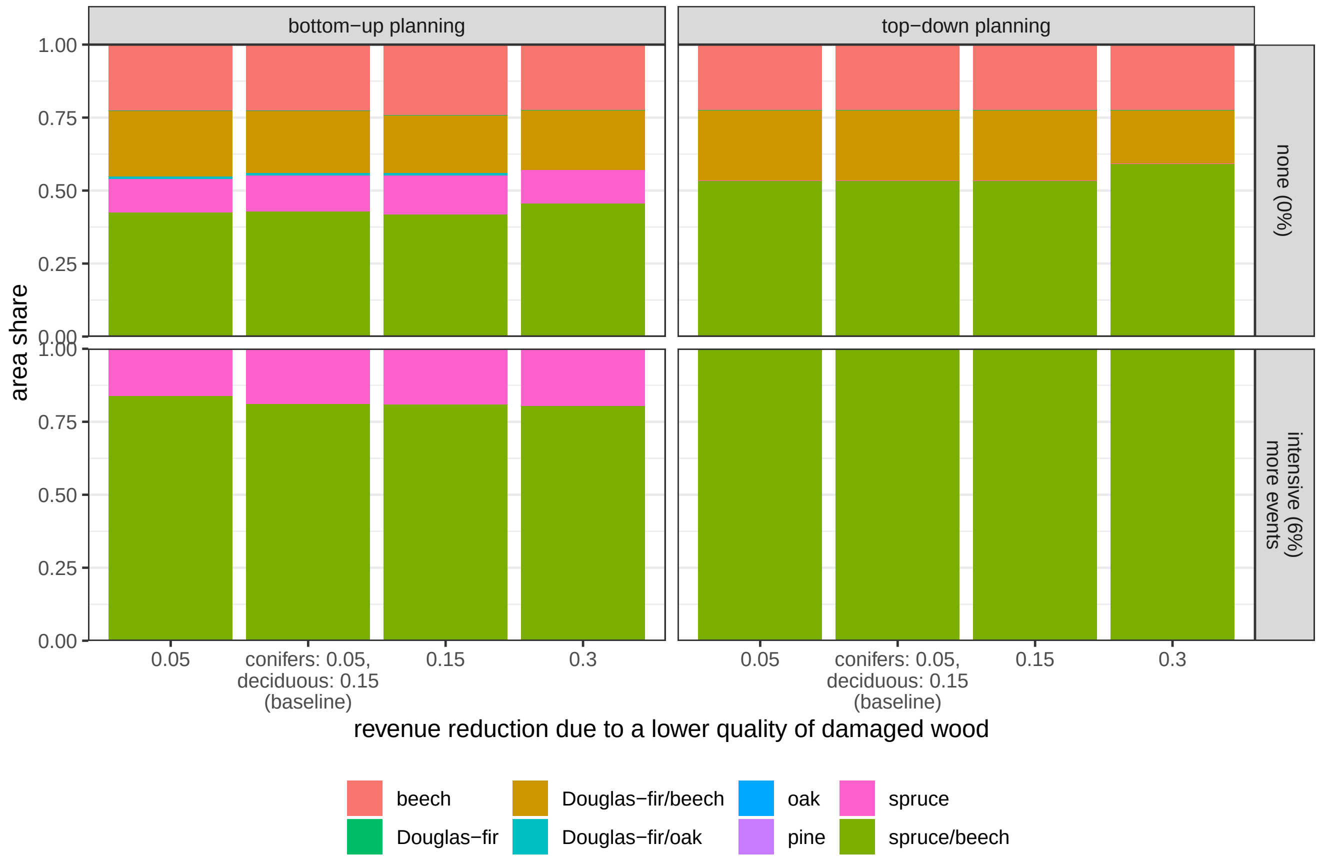

Figure A46. Optimal stand-type composition of the forest enterprise (γ-diversity) for different planning perspectives (panel columns) and two extreme-event scenarios (panel rows) dependent on the relative reduction in wood revenues for damaged wood.

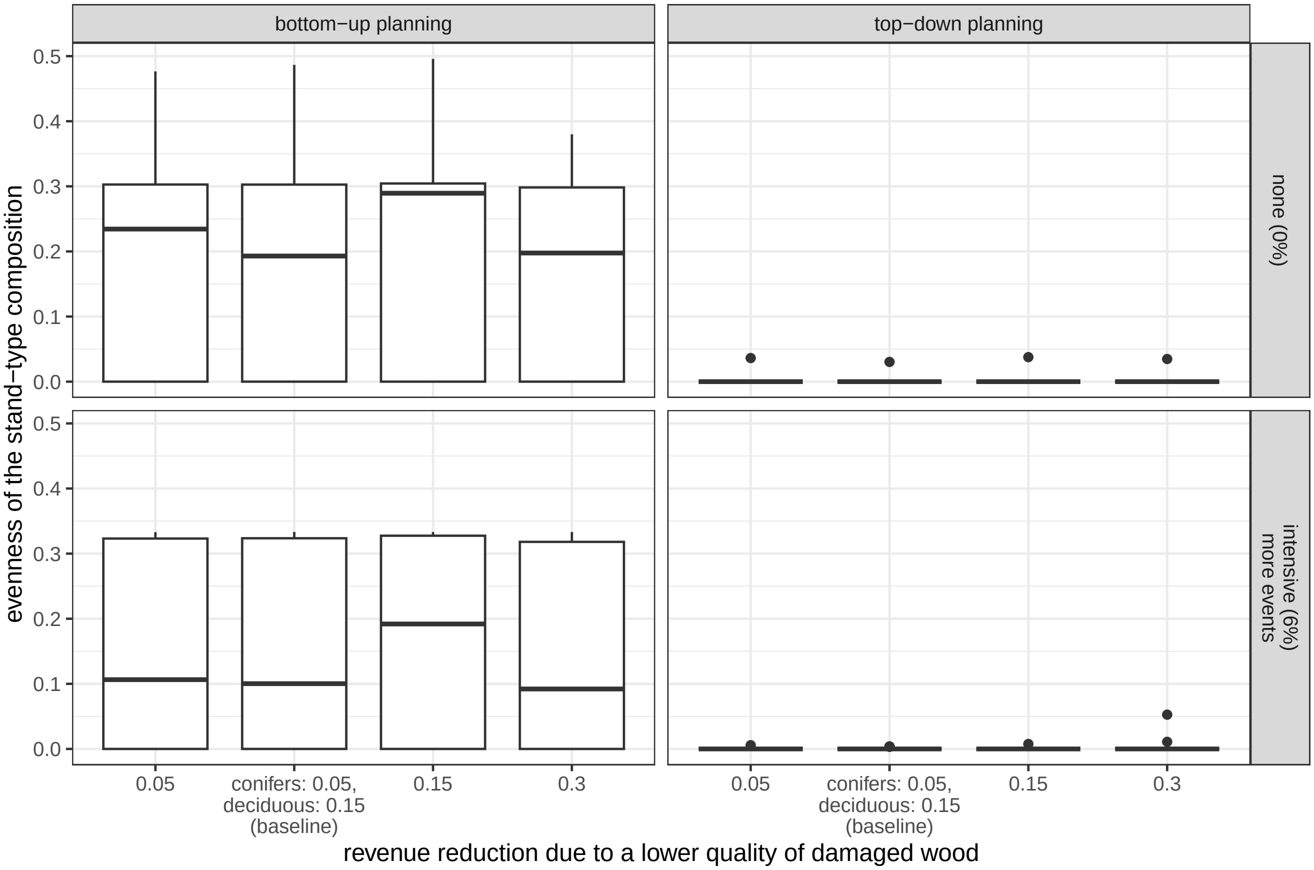

Figure A47. Evenness of the optimal stand-type composition of the forest enterprise (γ-diversity) for different planning perspectives (panel columns) and two extreme-event scenarios (panel rows) dependent on the relative reduction in wood revenues for damaged wood.

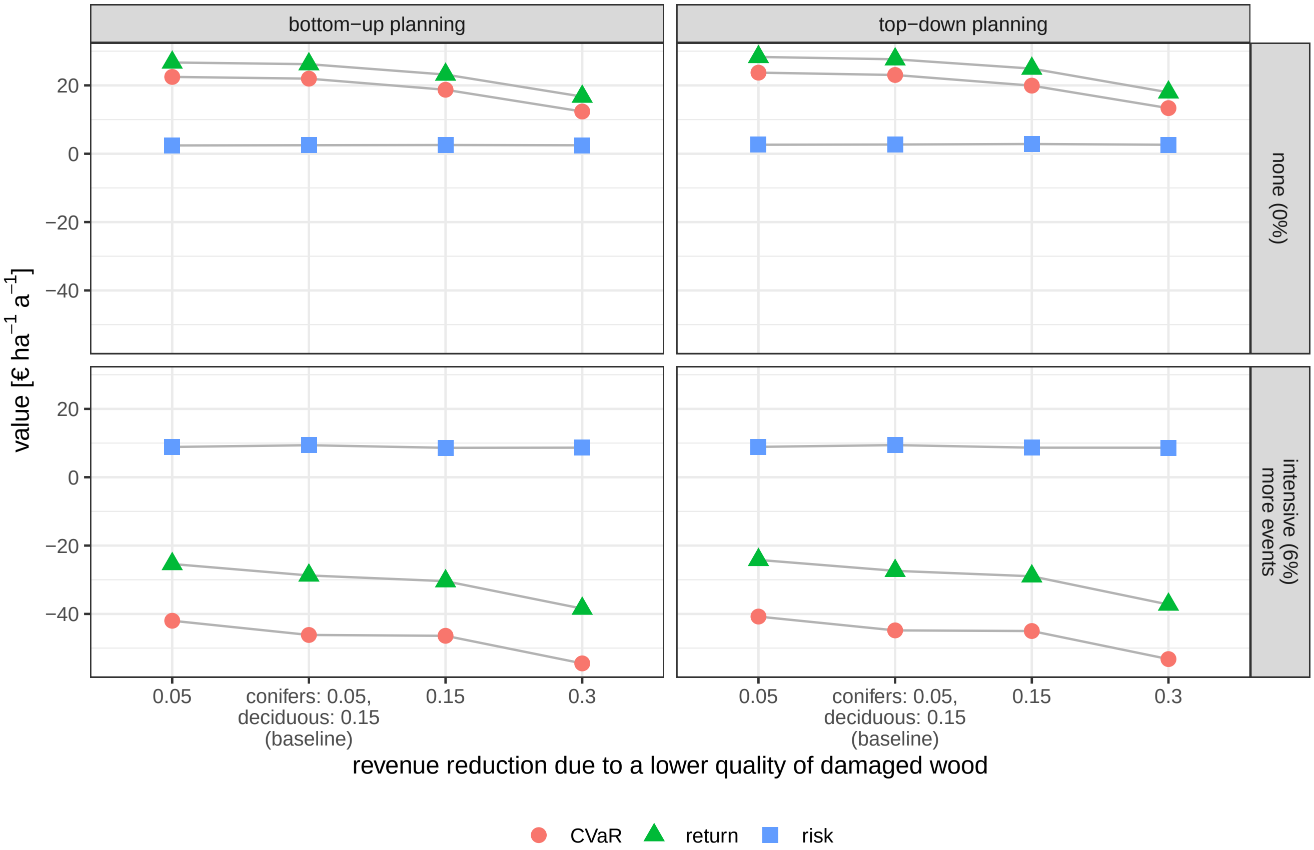

Figure A48. Economic variables of the forest enterprise under the optimal stand-type composition for different planning perspectives (panel columns) and two extreme-event scenarios (panel rows) dependent on the relative reduction in wood revenues for damaged wood. CVaR: Conditional Value at Risk (10 %-quantile), return: expected annuity, risk: standard deviation of the annuity.

A2.9.7 Quantile CVaR

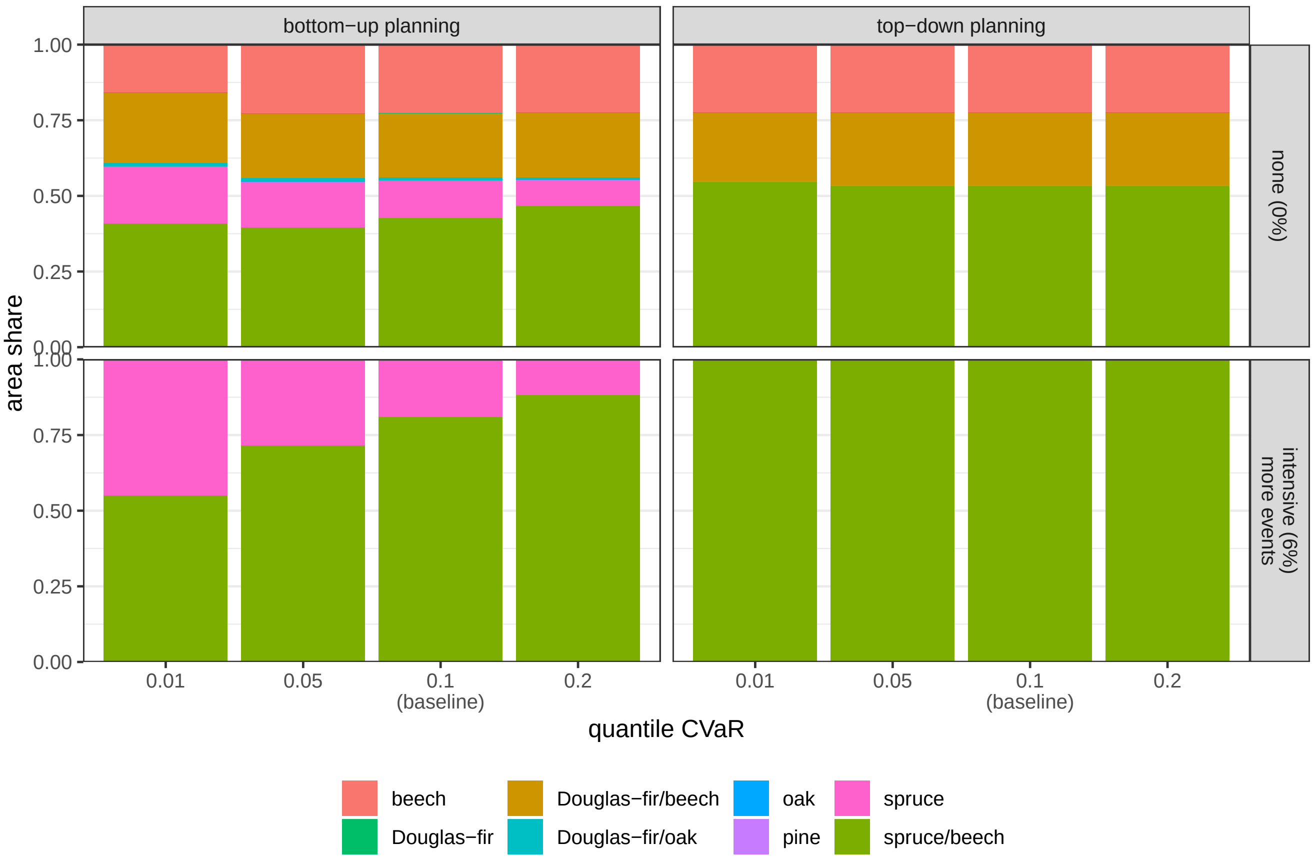

Figure A49. Optimal stand-type composition of the forest enterprise (γ-diversity) for different planning perspectives (panel columns) and two extreme-event scenarios (panel rows) dependent on the quantile applied in the *CVaR* calculation, i.e., the decision-maker's attitude towards risks.

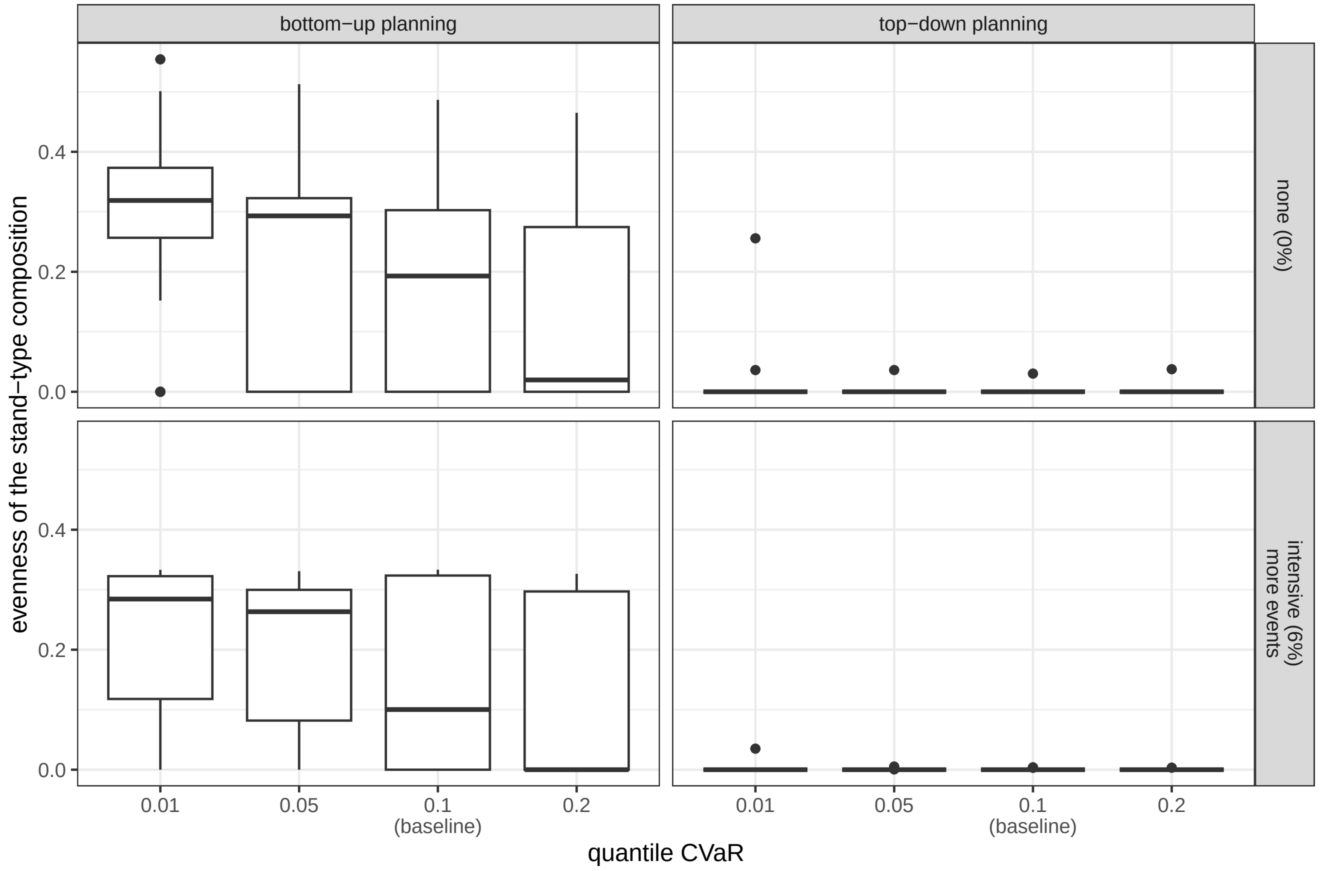

Figure A50. Evenness of the optimal stand-type composition of the forest enterprise (γ-diversity) for different planning perspectives (panel columns) and two extreme-event scenarios (panel rows) dependent on the quantile applied in the *CVaR* calculation, i.e., the decision-maker's attitude towards risks.

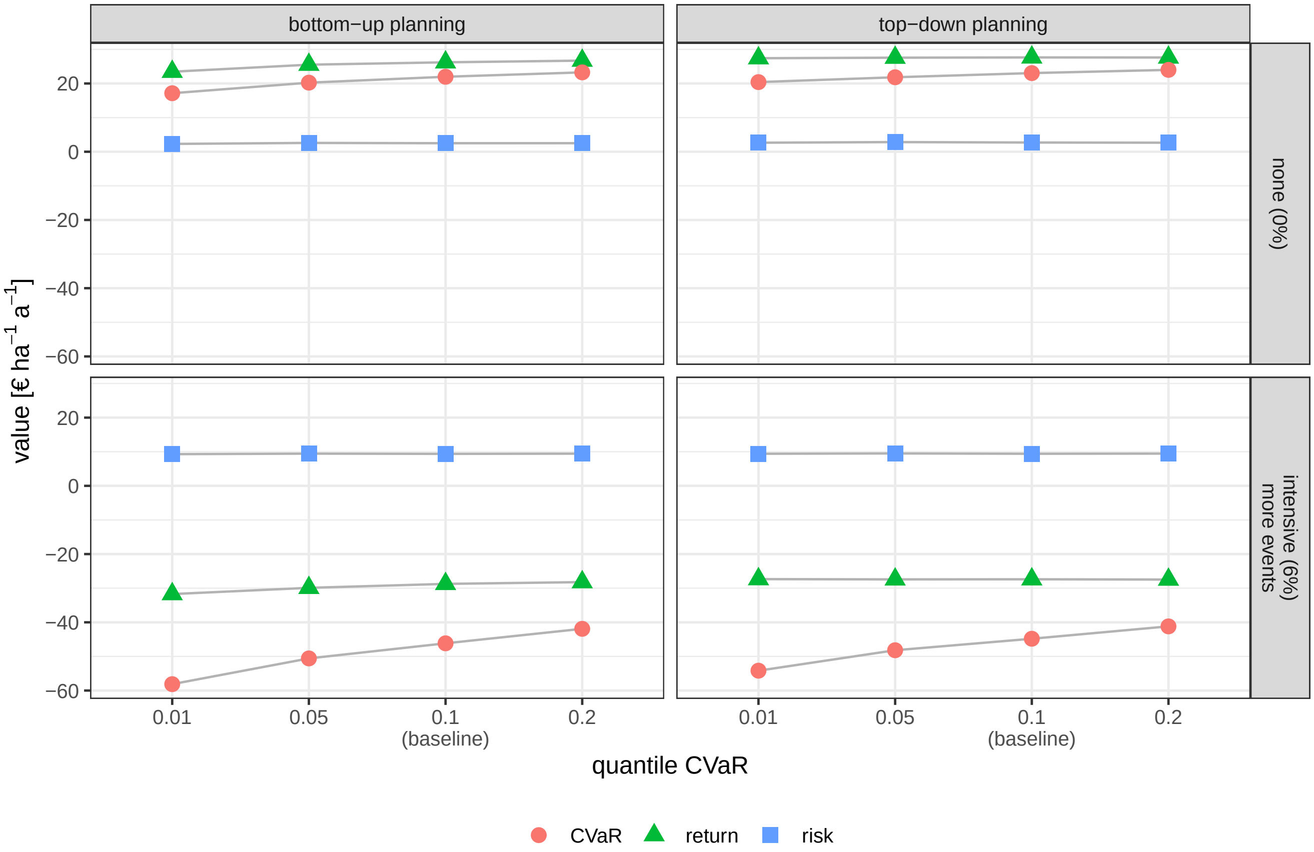

Figure A51. Economic variables of the forest enterprise under the optimal stand-type composition for different planning perspectives (panel columns) and two extreme-event scenarios (panel rows) dependent on the quantile applied in the *CVaR* calculation, i.e., the decision-maker's attitude towards risks. CVaR: Conditional Value at Risk, return: expected annuity, risk: standard deviation of the annuity.

A2.9.8 S_{100} *for extrapolated survival functions*

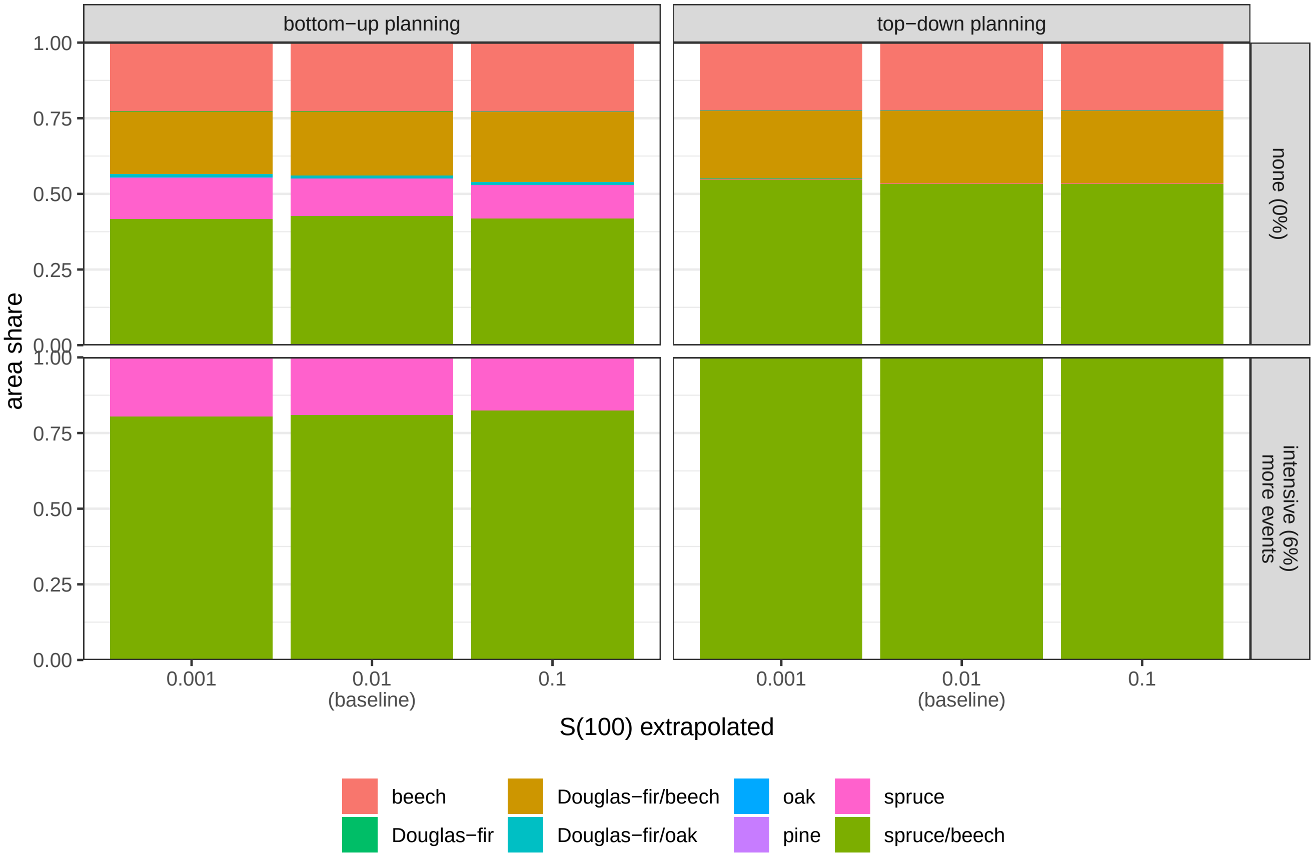

Figure A52. Optimal stand-type composition of the forest enterprise (γ-diversity) for different planning perspectives (panel columns) and two extreme-event scenarios (panel rows) dependent on the assumed probability of a stand to reach the age 100 (S_{100}) if the climate scenario predicted warmer and/or dryer climatic conditions at the stand's location than in the data of the respective tree species used to fit the survival functions of Brandl *et al.* [1].

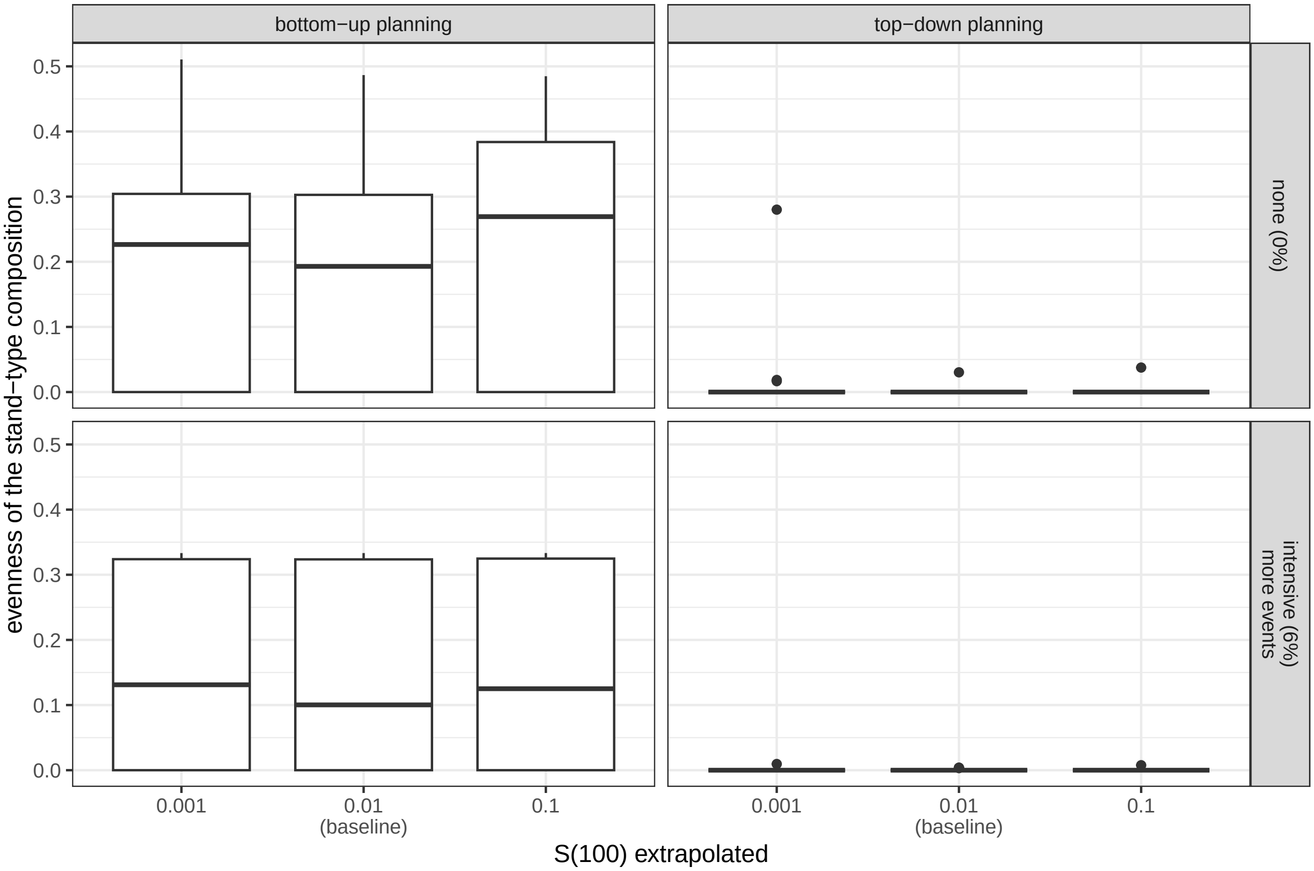

Figure A53. Evenness of the optimal stand-type composition of the forest enterprise (γ-diversity) for different planning perspectives (panel columns) and two extreme-event scenarios (panel rows) dependent on the assumed probability of a stand to reach the age 100 (S_{100}) if the climate scenario predicted warmer and/or dryer climatic conditions at the stand's location than in the data of the respective tree species used to fit the survival functions of Brandl *et al.* [1].

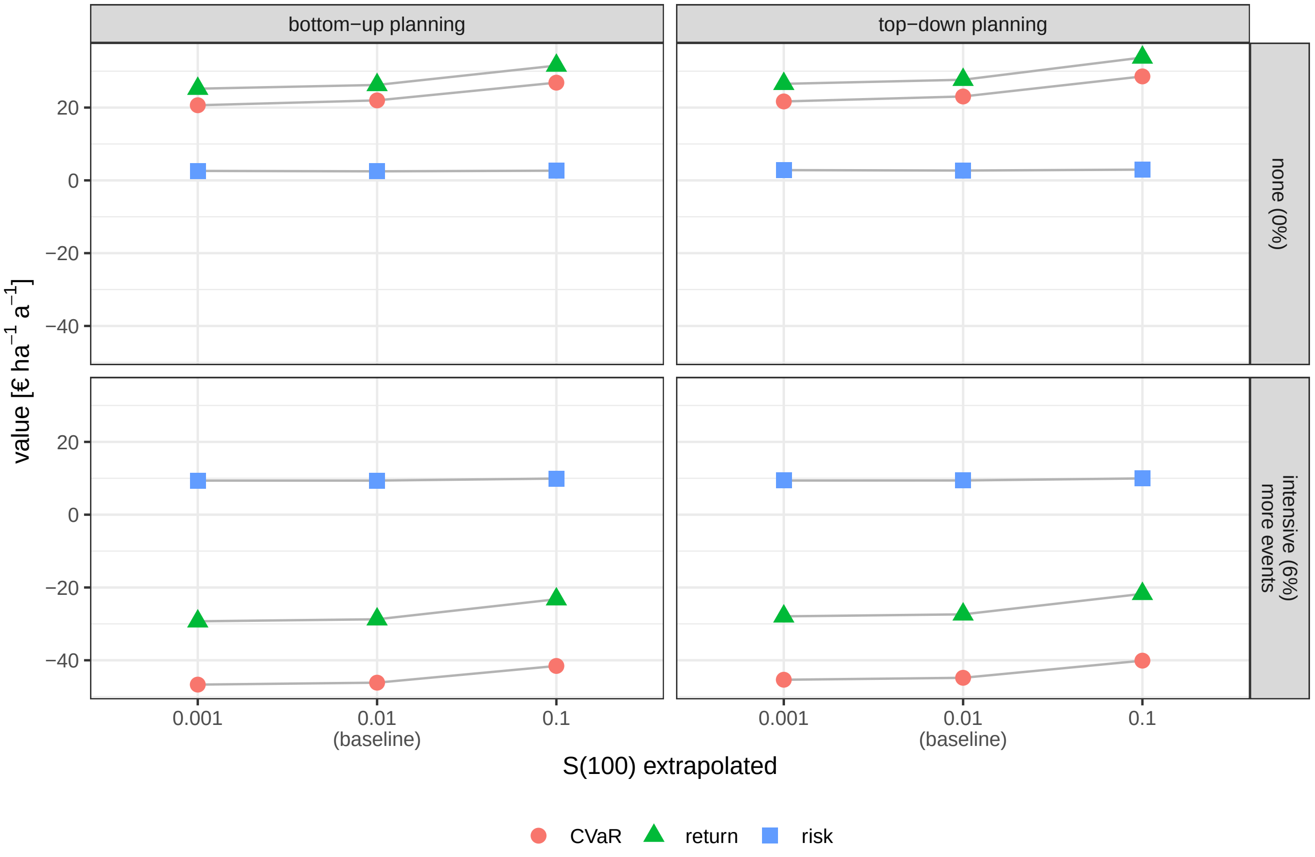

Figure A54. Economic variables of the forest enterprise under the optimal stand-type composition for different planning perspectives (panel columns) and two extreme-event scenarios (panel rows) dependent on the assumed probability of a stand to reach the age 100 (S_{100}) if the climate scenario predicted warmer and/or dryer climatic conditions at the stand's location than in the data of the respective tree species used to fit the survival functions of Brandl *et al.* [1]. CVaR: Conditional Value at Risk (10 %-quantile), return: expected annuity, risk: standard deviation of the annuity.

References

1. Brandl, S., Paul, C., Knoke, T. & Falk, W. The influence of climate and management on survival probability for Germany's most important tree species. *For. Ecol. Manag.* **458**, 117652, DOI: 10.1016/j.foreco.2019.117652 (2020).

2. Möllmann, T. B. & Möhring, B. A practical way to integrate risk in forest management decisions. *Ann. For. Sci.* **74**, 75, DOI: 10.1007/s13595-017-0670-x (2017).

3. Staupendahl, K. & Möhring, B. Integrating natural risks into silvicultural decision models: A survival function approach. *For. Policy Econ.* **13**, 496–502, DOI: 10.1016/j.forpol.2011.05.007 (2011).

4. Hijmans, R. J., Cameron, S. E., Parra, J. L., Jones, P. G. & Jarvis, A. Very high resolution interpolated climate surfaces for global land areas. *Int. J. Clim.* **25**, 1965–1978, DOI: 10.1002/joc.1276 (2005).

5. Staupendahl, K. Modellierung der Überlebenswahrscheinlichkeit von Waldbeständen mithilfe der neu parametrisierten Weibull-Funktion [Modelling the Survival Probability of Forest Stands Using the Parameterised Weibull Function]. *Forstarchiv* **82**, 10–19 (2011).

6. Paul, C. *et al.* Climate change and mixed forests: How do altered survival probabilities impact economically desirable species proportions of Norway spruce and European beech? *Ann. For. Sci.* **76**, 14, DOI: 10.1007/s13595-018-0793-8 (2019).

7. Fuchs, J. M. *et al.* woodValuationDE: A consistent framework for calculating stumpage values in Germany (technical note). *Allg. Forst- Jagdztg.* **193**, 16–29, DOI: 10.23765/afjz0002090 (2023).

8. Möhring, B. *et al.* Was kosten zunehmende Risiken im Wald? Konzept zur Quantifizierung von klimawandelbedingten Risikokosten bei der forstlichen Bewirtschaftung [What do increasing risks in the forest cost? Concept for quantifying climate change-related risk costs in forest management]. *Holz-Zentralblatt* **48**, 842–845 (2022).

9. Messerer, K., Pretzsch, H. & Knoke, T. A non-stochastic portfolio model for optimizing the transformation of an even-aged forest stand to continuous cover forestry when information about return fluctuation is incomplete. *Ann. For. Sci.* **74**, 2, DOI: 10.1007/s13595-017-0643-0 (2017).

10. Thünen-Institut. Dritte Bundeswaldinventur - Ergebnisdatenbank, https://bwi.info [Third National Forest Inventory in Germany - Results Database] (last access: 2023/06/27). Tech. Rep., Thünen-Institut, Braunschweig (2014).
